# Optimization of Antifungal 1,5-Diaryl-Pyrazole Acetyl-CoA Synthetase Inhibitors

**DOI:** 10.1021/acsomega.5c13157

**Published:** 2026-04-14

**Authors:** Jonah P. Propp, Jeffrey C. Ferreira, Parisa Enayati, Kathryn M. Alden, Drashti G. Daraji, Charles L. Lail, Michael E. Heene, Andrew J. Jezewski, Rohan Wakade, Damien Castor, Noelle S. Williams, Timothy J. Hagen, Damian J. Krysan

**Affiliations:** † Department of Pediatrics, Carver College of Medicine, 4083 University of Iowa, Iowa City, Iowa 52242, United States; ‡ Department of Chemistry and Biochemistry, 2848 Northern Illinois University, DeKalb, Illinois 60115, United States; § Department of Biochemistry, UT Southwestern Medical Center, Dallas, Texas 75390, United States; ∥ Department of Molecular Physiology and Biophysics, Carver College of Medicine, University of Iowa, Iowa City, Iowa 52242, United States

## Abstract

Acetyl-CoA Synthetases (ACS) have emerged as a potential drug target for the treatment of diseases ranging from fungal and parasitic infections to cancer and hyperlipidemia. While species-selective ACS inhibitors have been reported, broad-spectrum inhibition of ACS enzymes has only been achieved with nucleoside-derived, bisubstrate analogs such as alkyl esters of adenosine monophosphate (AMP). Previously, we identified the diaryl pyrazole AR-12 as a non-nucleoside inhibitor of *S. cerevisiae* ACS with broad-spectrum antifungal activity. In this study, we undertook a medicinal-chemistry approach to optimize the AR-12 scaffold with the goals of improving its pharmacology and activity against ACS enzymes from human fungal pathogens such as *Candida albicans*, *Cryptococcus neoformans*, and *Aspergillus fumigatus*. These efforts have led to the improved on-target activity for a set of analogs against multiple fungal enzymes. We identified key substituents that are critical determinants of the ACS inhibition displayed by this diaryl pyrazole class of inhibitors. Modeling and molecular dynamics calculations also indicate that these improved molecules likely bind in the same region of the ACS active site in which the alkyl-AMP esters bind. Finally, the most active diaryl pyrazole has improved mammalian cytotoxicity and pharmacology relative to AR-12. However, additional optimization of this class of ACS inhibitors will be needed to generate a molecule with efficacy in preclinical animal models.

## Graphical abstract

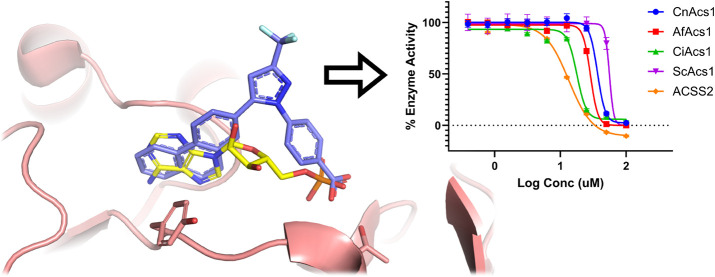

## Introduction

Human fungal infections cause a wide range of diseases, ranging from superficial mucosal and skin infections to bloodstream infections to deep organ infections of the brain, lung, and liver. Although the incidence and prevalence of human fungal infections are difficult to ascertain, recent estimates indicate that as many as 6.5 million people suffer from fungal infections each year.^1^ This anatomically diverse set of diseases is caused by an evolutionarily diverse number of fungal species. Despite the wide genomic distances between some of the most important human fungal pathogens, fungal pathogens are eukaryotes and are far more related to their human hosts than bacterial pathogens, for example. As such, identifying and exploiting drug targets that selectively affect fungal physiology without having toxic effects on the host remains one of the most difficult barriers to developing new therapies for human fungal infections.^2^

As one approach to circumventing this barrier, we have focused on a target enzyme that despite being highly conserved plays distinct roles in fungal and human cell physiology.^3–5^ Acetyl-Coenzyme A synthetase (ACS) converts acetate and Coenzyme A (CoA) to acetyl-CoA (AcCoA). AcCoA is a key molecule in biology with roles in central carbon metabolism, lipid biosynthesis, protein regulation, and gene expression.^4,5^ In mammals and some species of fungi, AcCoA is also synthesized by ATP-citrate lyase (ACL, refs 6 and 7). ACL functions in the cytosol and nucleus to convert citrate, which has been exported from the mitochondria, to AcCoA. Indeed, in humans and other mammals, the vast majority of cellular AcCoA is generated by ACL.^3^ Accordingly, mouse genetic experiments have shown that ACL is essential in mammals, while ACS is dispensable.^8,9^ Importantly, cancer cells have very different metabolisms compared to normal cells and demonstrate the Warburg effect.^10^ Part of this effect is the fact that AcCoA is generated through ACS in cancer cells with ACL providing a minority fraction.^3^ As such, ACS has emerged as an anticancer target and, indeed, an ACS inhibitor has entered early-stage clinical trials.^11^

*Candida spp*. and their non-Candida relatives (e.g., *Candidozyma auris* and *Nakesomyces glabratus*) are among the most common human fungal pathogens and cause both mucosal disease and life-threatening invasive disease.^12^ This set of human pathogens along with the model organism *Saccharomyces cerevisiae* are dependent on ACS for the production of AcCoA because their genomes lack ACL. Consistent with this, genetic data indicate that the ACS enzymes are essential in these fungi.^13,14^ Therefore, we have been interested in developing ACS inhibitors as mechanistically novel antifungal molecules. In prior work, we reported that the celecoxib-related molecule AR-12 (Figure 1) had broad-spectrum antifungal activity and inhibited *S. cerevisiae* ACS.^15,16^ However, AR-12 has been reported to have a wide range of biological activities, suggesting it interacts with multiple cellular targets.^17^ Consistent with this possibility, AR-12 shows a modestly high mammalian cell toxicity. In a phase I human trial as an anticancer agent,^18^ serum concentrations of AR-12 varied significantly from patient-to-patient indicating heterogeneous metabolism and a potential efficacy-limiting liability.

**Figure 1:**
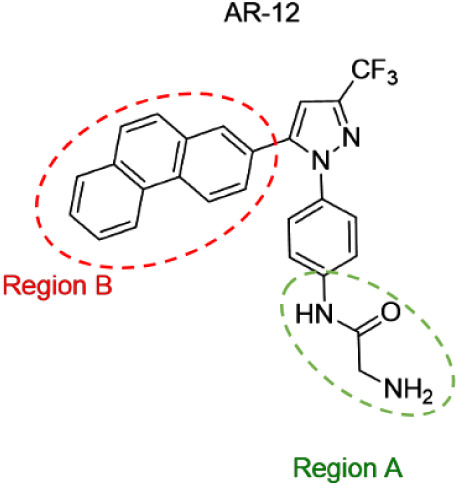
Chemical structure of AR-12 denoting the regions of focus for structure–activity optimization.

In other work, we have shown that ACS inhibitors show surprisingly high selectivity toward fungal ACS^19^ given the sequence and structural similarity among eukaryotic ACS enzymes.^20^ For example, we identified a cyclopropyl-isoxazole that inhibits *C. neoformans* Acs1 with *K*_i_ of 8 μM, while having essentially no activity against other fungal enzymes (IC_50_ > 100 μM, ref 19). Similarly, antiplasmodial ACS inhibitors have greatly reduced activity against fungal and human ACS enzymes.^19^ Consistent with this trend, AR-12 showed a poor spectrum of activity against other fungal ACS (*vide infra*), further suggesting that its antifungal activity may be driven by off-target effects.^19^

We hypothesized that the diarylpyrazole scaffold AR-12 might serve as a starting point for a medicinal chemistry-based optimization of on-target activity that would lead to molecules with a broad-spectrum ACS inhibition profile. Biochemical and structural data indicate that the highly selective ACS inhibitors targeting plasmodium ACS and CnAcs1 interact with the CoA binding pocket.^19^ We previously showed that AR-12 was competitive with ATP^15^ and we further hypothesized that molecules targeting the ATP/acetyl-AMP pocket of the enzyme may have a broader spectrum of activity. As described below, we have generated analogs that inhibit ACS enzymes from multiple species of human fungal pathogens as well as reduced toxicity and improved pharmacological properties in a mouse model.

## Results

### Acidic Groups at Region A Are Critical for ACS Inhibition by Diaryl Pyrazoles

While AR-12 showed potent in vitro activity against ScAcs1, we found that it showed poor and variable activity against the *C. albicans* Acs2 (20–50% inhibition at 50 μM with no calculable IC_50_ due to solubility issues). Since other chemical scaffolds have shown species-specific ACS inhibition, we undertook a medicinal chemistry exploration of the AR-12 scaffold with the initial goal of identifying diaryl-pyrazoles with activity against *C. albicans* Acs2 as well as other fungal ACS enzymes. We focused on two regions of the molecule indicated by regions A and B in Figure 1. First, we synthesized a set of compounds to explore the structure–activity relationship (SAR) of region A with a focus on testing the effect of ionized moieties at this position (Schemes 1–2). The phenanthrene ring in region B was maintained as a control. This set of AR-12 derivative compounds was screened against *Cryptococcus neoformans* CnAcs1 and *Candida albicans* CaAcs2 at 50 μM and 25 μM to expediently identify enzyme inhibitors for further characterization (Table 1).

**Scheme 1:**
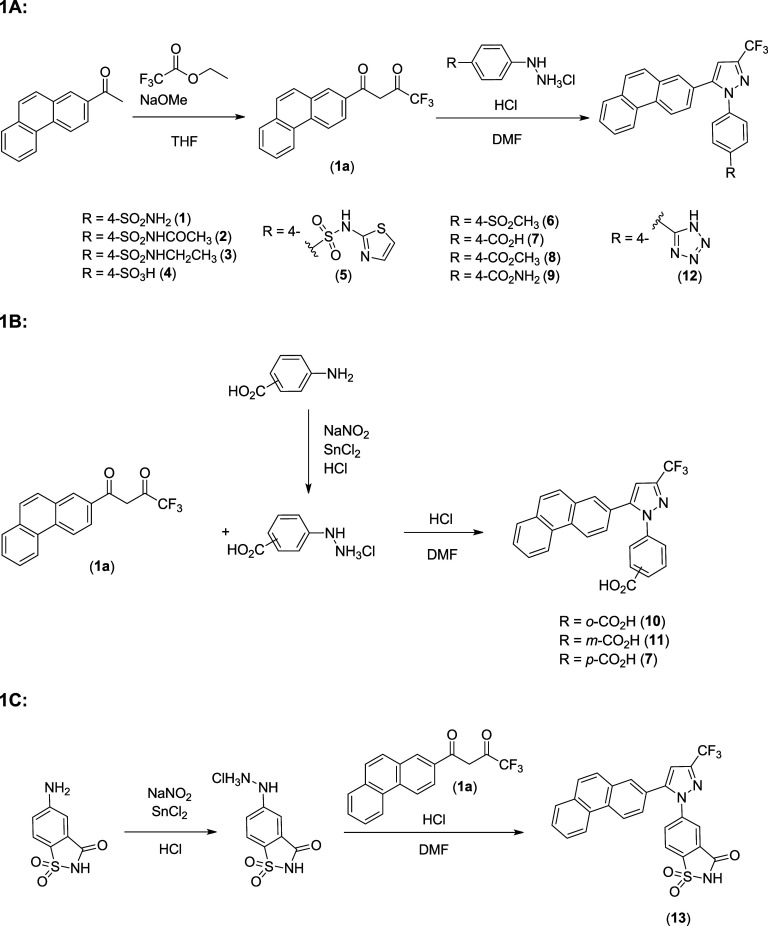
General Procedure for the Synthesis of Compounds **1**–**13**; 1A: Synthesis of Analogs with a Varied Substituents at the *p* Position of Region A; 1B: Synthesis of Carboxylic Acid Analogs at *o*, *m*, and *p* Positions of Region A; 1C: Synthesis of Analog with Saccharin Moiety at Region A

**Scheme 2:**
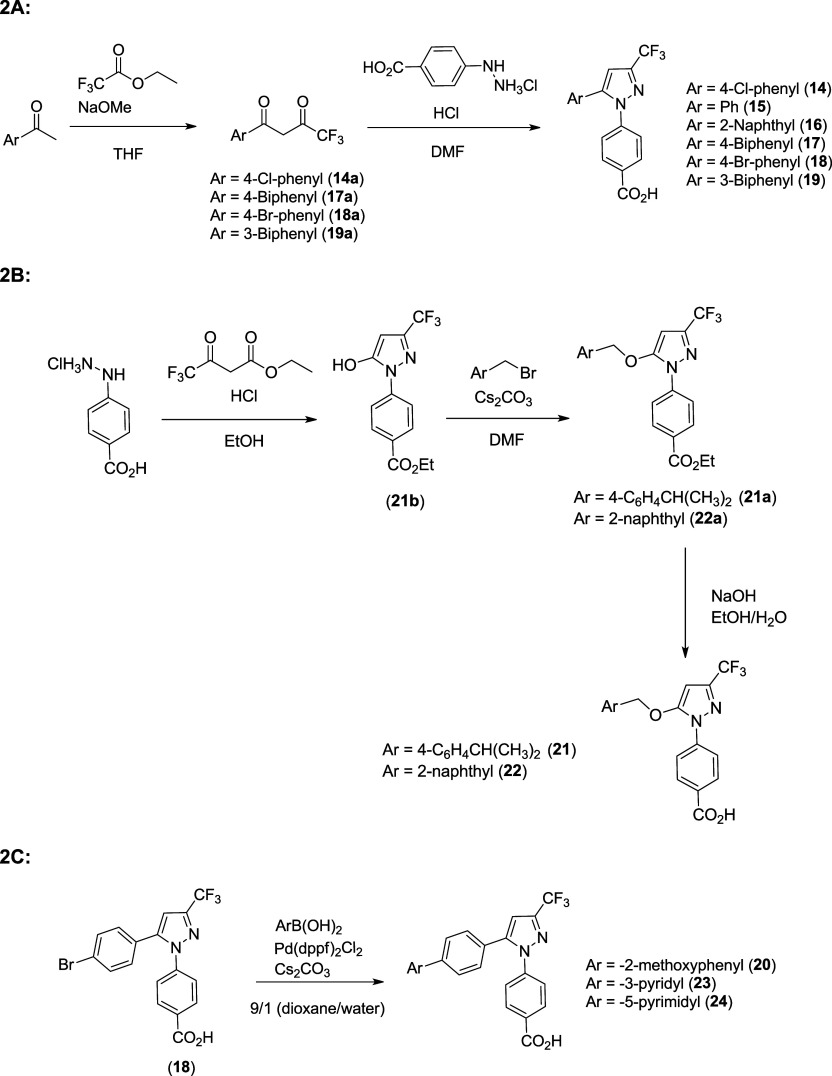
Synthetic Routes for the Phenanthrene Replacements (Compounds **14**–**24**); 2A: Synthesis of Analogs with Various Aryl Groups at Region B; 2B: Synthesis of Analogs with −CH_2_Ar Ether Linkages at Region B; 2C: Synthesis of Analogs with Extended Aryl Groups at Region B

**Table 1:**
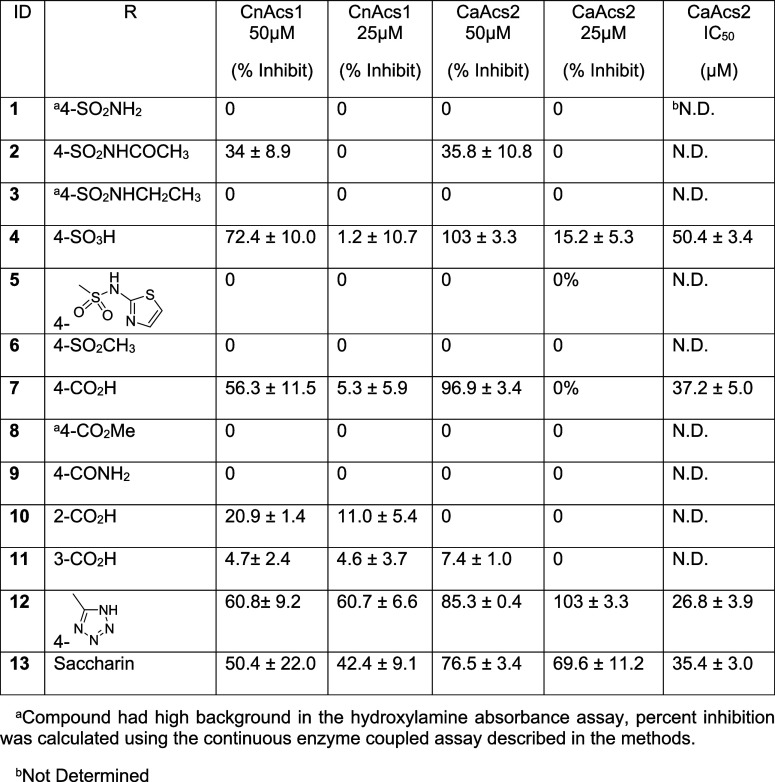
ACS Inhibition by Region A Analogs of AR-12

Two different assays of ACS activity were used to evaluate the compounds. First, a well-established end-point assay was used, in which the AcCoA reaction product is measured through its reaction with hydroxylamine to form the corresponding hydroxamic acid. The acetyl-hydroxamate is detected as an iron complex by absorbance.^15^ Some of the compounds generated high background at the wavelength required for this assay (540 nm). For high-background compounds, a continuous, coupled assay in which the pyrophosphate generated by the first step of the enzymatic reaction is converted to phosphate by pyrophosphatase and detected by its subsequent reaction with 7-methyl-6-thioguanosine (MeSG) mediated by purine nucleoside phosphorylase, which was previously optimized for these enzymes.^20^ The IC_50_ values were determined for molecules with >50% inhibition at 50 μM.

The general method employed for the preparation of 1,5-diarylpyrazoles **1**–**3**, illustrated in Scheme 1A, involves the condensation of a 1,3-dicarbonyl compound with an aryl hydrazine hydrochloride in the presence of an acid.^21^ The phenanthryl 1,3-diketone (**1a**) starting material was synthesized through a Claisen condensation of 2-acetylphenanthrene with ethyl trifluoroacetate in the presence of NaH to afford the product in quantitative yield. Purification involved only an acidic extraction in EtOAc. Various commercially available aryl hydrazine hydrochlorides were employed for the Knorr pyrazole synthesis in either refluxing EtOH or hot DMF to afford the products (**1**,**4**,**6**). The 4-sulfonamide analog (**1**) served as a common intermediate. Acylation of the sulfonamide was carried out with *N*-acetoxy succinimide in the presence of Cs_2_CO_3_ in THF to afford the product (**2**) in an excellent yield. Alkylation of the sulfonamide with bromoethane in K_2_CO_3_ in acetone afforded the singly ethylated product (**3**) in moderate yield. The hydrochloride hydrazines of the *N*-thiazole and tetrazole analogs (**5**,**12**) were not commercially available. Therefore, they were synthesized through a Sandmeyer-like process in which the diazonium ion, generated through NaNO_2_ in aqueous acid, was reduced through the addition of SnCl_2_. The hydrazine hydrochloride products were utilized without further purification in a Knorr pyrazole synthesis to afford the *N*-thiazole and tetrazole analogs (**5**,**12**) in good yield. Compound (**13**) was synthesized via an analogous synthesis according to Scheme 1C, using diazotization, reduction, and Knorr pyrazole synthesis starting with 5-aminosaccharin.

The carboxylic acid derivatives (**7**–**11**) were synthesized in a similar manner (Scheme 1A–B). Each hydrazine hydrochloride was obtained through the same Sandmeyer-like method as that shown in Scheme 1A. However, the Knorr pyrazole synthesis step was performed at an elevated temperature in DMF, rather than in refluxing EtOH, to avoid any unwanted Fischer esterification.

Replacement of the glycine moiety of AR-12 with the sulfonamide of DMC (**1**) dramatically reduced the activity. Placement of an acetyl group on the sulfonamide (**2**) improved activity marginally; the p*K*_a_ of acetyl sulfonamides is similar to that of a carboxylic acid (in the 3–4 range), suggesting that negative charge may be advantageous at this position. Consistent with this hypothesis, the sterically similar but electronically distinct ethyl-sulfonamide (**3**) showed no improvement in activity. Further supporting this hypothesis, the sulfonic acid derivative (**4**) was much more active and showed an IC_50_ of 50 μM, while the *N*-thiazoyl (**5**) and sulfone (**6**) containing molecules were completely inactive.
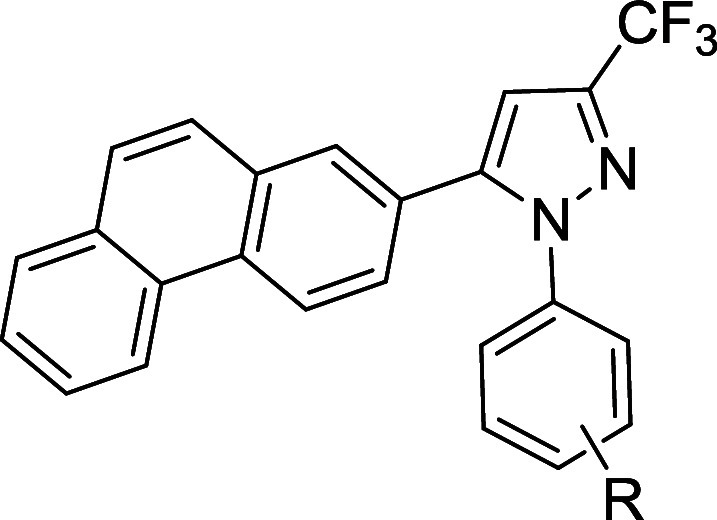


Next, we synthesized the 4-carboxylate derivative (**7**) and were pleased to see that it had substantial activity. Consistent with the importance of an acidic functional group at this position, the methyl ester (**8**) and amide (**9**) derivatives of **7** were inactive. To determine if the positioning of the carboxylate group on the aryl ring was important, we synthesized analogs with 2- and 3- carboxylates (**10** and **11**); both derivatives were less active than the 4-carboxylate. The p*K*_a_ of aryl-tetrazoles is also similar to those of carboxylic acids; therefore, we synthesized the 4-tetrazole derivative of AR-12 (**12**) and found its activity to be comparable to **7**. Finally, the benzosulfimide moiety of the saccharin ring is also ionized at physiological pH with a p*K*_a_ of ∼2. Consistent with the other data, the corresponding saccharin-derived analog (**13**) had similar potency to **7** and **12**. Taken together, these data strongly support the requirement of a negatively charged group at the 4-position of the ring in region A as advantageous for ACS inhibition.

### Phenanthrene Is Highly Favored at Region B

Next, we explored the SAR of region B in the context of analogs of the 4-carboxylate-containing compound **7**. We focused on incorporating aryl hydrophobic substituents of varying sizes, geometries, and conformations (Table 2). These included molecules with aromatic rings attached directly to the pyrazole (**14–20**), as well as compounds containing an ether linkage to the same position (**21**, **22**). In addition, nitrogen heterocycles were tested (**23** and **24**). Much to our surprise, none of the alternate hydrophobic scaffolds inhibited either fungal ACS. These data indicate a critical role of the phenanthrene ring, which cannot be recapitulated with naphthalene, biphenyl, or other hydrophobic groups.

**Table 2:** ACS Inhibition by Region B Analogs of **7**

ID	R	CnAcs1 50 μM (% Inhibit)	CnAcs1 25 μM (% Inhibit)	CaAcs2 50 μM (% Inhibit)	CaAcs2 25 μM (% Inhibit)
**14**	4-Cl-phenyl	0	0	0.5 ± 4.2	7.4 ± 10.5
**15**	Phenyl	0	0	2.9 ± 5.1	0
**16**	2-Naphthalenyl	0	0	0	0
**17**	4-Biphenyl	4.1 ± 1.38	2.3 ± 5.5	0	0
**18**	4-Br-phenyl	0	0	0	0
**19**	3-Biphenyl	0	0	0	0
**20**	2-Methoxy-1,1′-biphenyl	0	0	0	0
**21**	4-Isopropyl-benzyl)oxy	0	0	7.6± 6.3	0
**22**	Naphthalen-2-yl-methoxy	0	0	8.7±11.4	0
**23**	3-Phenylpyridine	0	0	2.0 ± 3.7	0
**24**	5-Phenylpyrimidine	0	0	0	0

Replacement of the phenanthryl moiety of compound (**7**) employed the same synthetic pathway as the phenanthryl analog, via Claisen condensation of the acetyl aryl compound with ethyl trifluoroacetate followed by a Knorr pyrazole synthesis with 4-hydrazinylbenzoic acid hydrochloride to form compounds **14**–**19** in good yields per Scheme 2A. As shown in Scheme 2C, the 4-bromophenyl analog (**18**) was further functionalized to form compounds (**20**, **23–24**) through a Suzuki reaction with various aryl boronic acids utilizing Pd­(dppf)­Cl_2_ and Cs_2_CO_3_ in a 9:1 (v/v) mixture of 1,4-dioxane/H_2_O, respectively, in excellent yields.

Analogs that employed the ether linkage at position 5 of the pyrazole and the 4-benzoic acid (**21**-**22**) were synthesized through a Knorr pyrazole synthesis of the hydrazinyl-benzoic acid and ethyl trifluoroacetoacetate in refluxing EtOH with HCl (Scheme 2B). This process not only afforded the 5-hydroxy pyrazole but also converted the carboxylic acid to the corresponding ethyl ester (**21b**). The protection of the carboxylic acid as the corresponding ethyl ester allowed for convenient selective alkylation of the hydroxyl group without alkylating the carboxylic acid ester (**21a, 22a**). The esters were hydrolyzed utilizing NaOH in an EtOH/H_2_O mixture at room temperature to afford the benzoic acid analogs (**21**–**22**) in good yields.
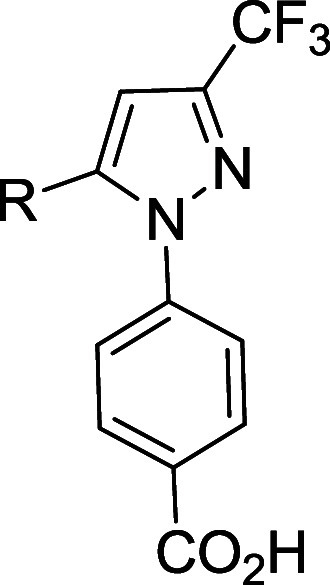


We also synthesized three saccharin derivatives with different region B aryl substituents (Table 3). The biphenyl- (**25**) and naphthalene-substituted (**26**) saccharin derivatives had slightly improved activity relative to the molecules with the phenyl-4-carboxylate analogs, but none showed greater than 50% inhibition at 50 μM. Although a large number of additional molecules could be synthesized to provide a more comprehensive assessment of region A, it appears that a large aromatic substituent such as phenanthrene is needed. Further increasing the hydrophobic nature at this position is likely to reduce its pharmacological tractability. Therefore, we elected not to pursue further optimization at this position.

**Table 3:** ACS Inhibition by Region B Analogs of **13**

ID	R	CnAcs1 50 μM (% Inhibit)	CnAcs1 25 μM (% Inhibit)	CaAcs2 50 μM (% Inhibit)	CaAcs2 25 μM (% Inhibit)
**25**	4-Biphenyl	25.7 ± 11.9	2.9 ± 2.8	34.8 ± 6.9	11.0± 1.7
**26**	2-Naphthalenyl	6.1 ± 7.8	0	17.0 ± 6.9	0
**27**	4-Chlorobenzyloxy	0%	0%	25.3 ± 4.5	11.8 ± 5.6

The synthesis of analogs employing a cyclic acyl-sulfonamide (Scheme 3) begins with the diazotization/reduction of the 5-aminosaccharin into the corresponding hydrazine hydrochloride. These analogs are referred to as saccharin analogs because the bottom aryl ring, benzo­[*d*]­isothiazol-3­(2*H*)-one 1,1-dioxide, was sold commercially as an artificial sweetener called saccharin. The Knorr pyrazole synthesis step was generally carried out at elevated temperatures in acidic DMF to form analogs (**25–26**). The use of acidic EtOH as the solvent in the Knorr pyrazole formation step resulted in the ring-opening of saccharin to produce the 4-ethylcarboxylate-5-sulfonamido phenyl products. The saccharin ring can be successfully restored under basic conditions, as proved to be the case in analog **27.**

**Scheme 3:**
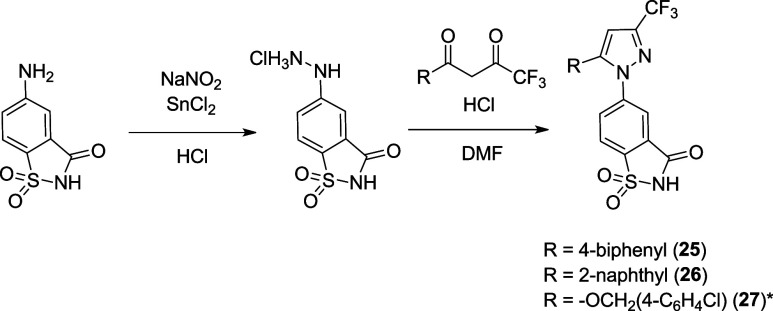
Synthetic Route for the Saccharin Analogs (**25**–**27**)­^a^

a *Proceeded through the same synthetic pathway, but with accidental ring opening and then subsequent ring closure of the saccharin. See Supporting Information for more details.


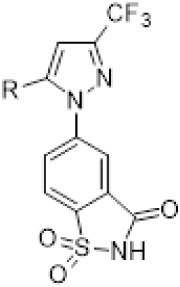


### Compounds **7** and **13** Inhibit Multiple Fungal ACS and Human ACSS2 Enzymes

To characterize the broader enzyme activity of AR-12 analogs, we tested the activity of **7** and **13** against a spectrum of ACS enzymes (Table 4, Figure S1), including other fungal ACSs and the human ACS (ACSS2). In general, **7** and **13** showed IC_50_ values in the 15–70 μM range against the fungal and human ACS enzymes, demonstrating similar inhibitory activity toward acetyl-CoA synthetase enzymes from the major human fungal pathogens *C. neoformans*, *A. fumigatus*, and *C. immitis* (Table 4). *C. neoformans* aceto-acetyl CoA synthetase (CnKbc1) has the same mechanism as the ACS enzymes but converts acetoacetate, a ketone body, to aceto-acetyl CoA.^22,23^ As such, CnKbc1 utilizes a slightly larger substrate compared to ACS. Interestingly, both **7** and **13** inhibit CnKbc1, while other reported ACS inhibitors do not.

**Table 4:** IC_50_ Values for **7** and **13** toward Acyl-CoA Synthetases

Enzyme	**7** (IC_50_, μM)	**13** (IC_50_, μM)
*C. neoformans* CnAcs1	36.6 ± 3.8^a^	17.7 ± 1.4
*A. fumigatus* AfAcs1	28.6 ± 7.61	16.7 ± 1.3
*C. immitis* CiAcs1	17.8 ± 5.72	11.9 ± 0.8
*S. cerevisiae* ScAcs1	55.7 ± 3.2	26.4 ± 3.3
*H. sapiens* ACSS2	69.2 ± 28.9	13.3 ± 3.8
*C. neoformans* CnKbc1	22.2 ± 3.6	8.2 ± 2.3

a IC_50_ error calculated from the 95% confidence interval of the curve fit.

## Computational Modeling

In order to identify a potential binding site and ligand conformation for the AR-12-based ACS inhibitors, we performed molecular docking and molecular dynamics (MD) studies. Since broad-spectrum inhibition of ACS enzymes has previously only been observed with alkyl adenosine monophosphate (AMP) esters, we began our investigations by looking at the ATP binding pocket. Supporting this modeling, compound **7** showed evidence of ATP-competitive inhibition by Lineweaver–Burke analysis (Figure S3) and AR-12 also showed ATP competitive inhibition against ScAcs1.^15^ Several X-ray crystal structures of ACS enzymes with AMP esters bound are available, so we began by docking **7** into various enzymes to identify a complex with favorable docking scores and ligand interactions. We ultimately selected the structure of CaAcs2 bound to adenosine-5′-allylphosphate (PDBID: 8V4P, ref 23) and used the chain C ATP-binding pocket for further analysis.

Molecular docking is often plagued by high false positive rates, requiring a rigorous validation process to ensure quality results. The generation of a receiver operating characteristic (ROC) curve allows for the quantitative evaluation of docking procedures by plotting the sensitivity against 1-specificity.^24^ This is done by designating true binders and false binders to test whether docking can discriminate between the two groups. Sensitivity (the true positive rate) is defined as the proportion of actual positive hits correctly identified by the model, calculated as true positives/(true positives + false negatives). 1-Specificity is the false positive rate, calculated as false positives/(true negatives + false positives). To generate the ROC curve, these two rates are plotted against each other.^24^ The area under the curve (AUC) for the ROC plot corresponds to docking performance, with an AUC of 0.5 providing no validation to docking procedures and an AUC of 1 being a theoretically perfect docking protocol. In our case, we assigned **4**, **7**, and **12** as actives, while **1, 2, 5, 6, 9–11, 14–17,** and **19–24** were assigned as inactive molecules. Docking scores ranked the active molecules as more favorable than the inactive molecules, demonstrating a strong correlation between enzyme inhibition and docking scores (Table S1). The ROC curve (Figure S2) indicated an AUC of 0.93, which strongly supports the hypothesis that these ligands bind in the ATP pocket.

Inspection of the docking pose for **7** indicates two direct interactions with enzyme residues Tyr443 and Thr446 (Figure 2A). Tyr443 forms an edge-to-face π-stacking interaction with the phenanthrene ring, while Thr446 is oriented with its hydroxyl group 2.9 Å from the carboxylate of **7**. Similar interactions are observed in the crystal structure of AMP-bound CaAcs2 (8V4O, ref 20), as shown in yellow in Figure 2B. Alignment of the docked pose of **7** with the crystal structure pose of AMP reveals close agreement of residue interactions in the regions of focus. In region A, the carboxylate closely aligns with AMP’s phosphate group and provides a rationale for the presence of negatively charged moieties in region A of active ACS inhibitors in this series. In region B, the phenanthrene ring overlaps with the adenine portion of AMP, forming a similar π interaction with Tyr443.

**Figure 2:**
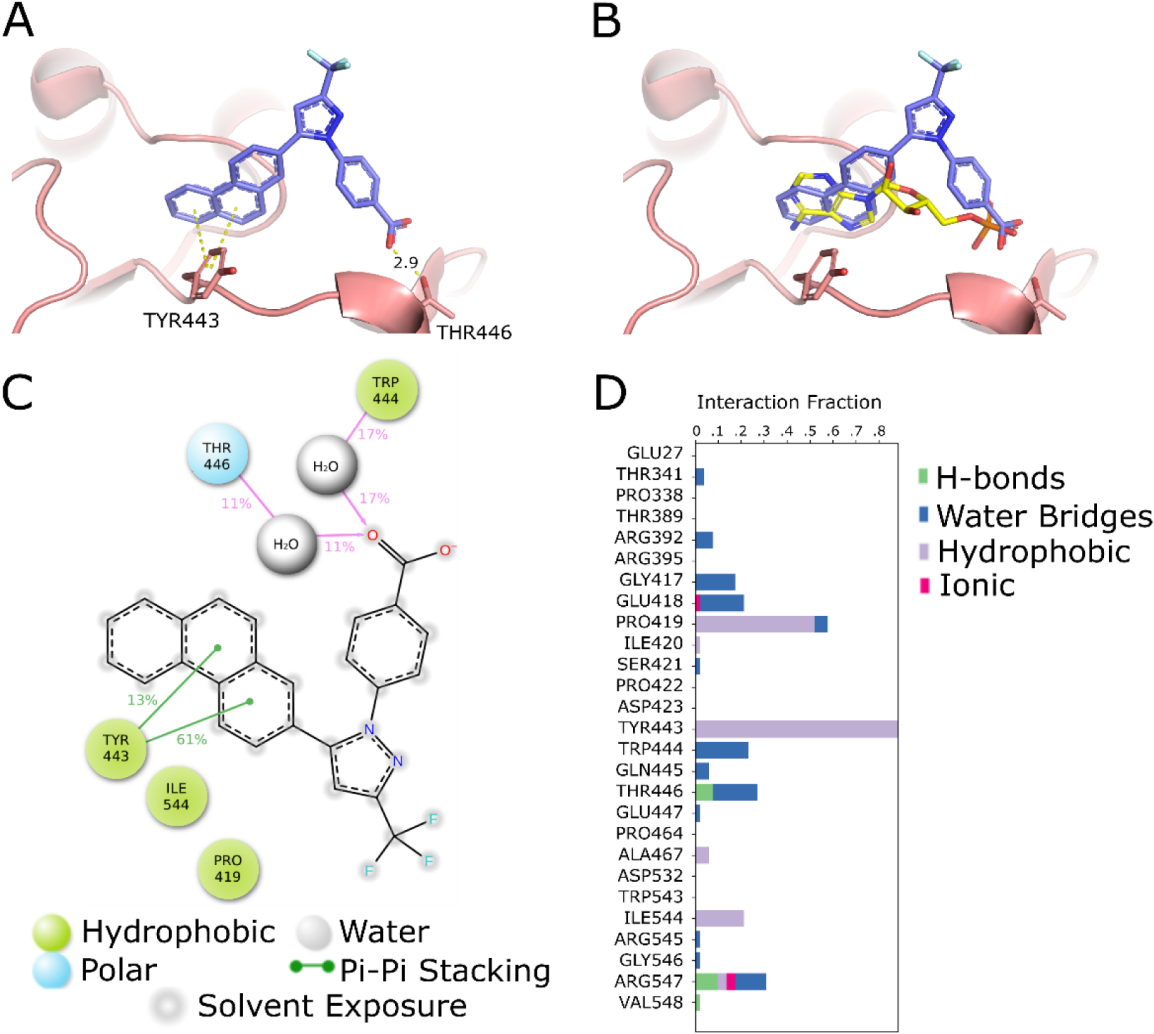
Docking pose and MD simulation of compound **7** in the ATP-binding pocket. A. **7** (purple) shows favorable docking in the ATP-binding pocket of CaAcs2, forming edge-to-face π-stacking with Tyr443 and hydrogen bonding with Thr446. B. Overlay of **7** with AMP (yellow) demonstrates that the hydrophobic/planar phenanthrene ring aligns with the adenine ring, while the carboxylate aligns with the phosphate. C. 2-D interaction diagram from a 1000 ns MD simulation of **7** in the ATP-binding site. D. Histogram of MD residue interactions between **7** and CaAcs2.

We further characterized the binding interaction of CaAcs2 with compound **7** by performing an MD simulation (Figure 2C and D). Throughout the trajectory of the simulation, the phenanthrene moiety remained deeply buried in the adenine-binding hydrophobic cavity and formed stable π–π and van der Waals interactions with Tyr443. Additional hydrophobic contacts with Pro419, Ile544, and Val548 were also observed and contributed to the binding of the inhibitor to CaAcs2. The carboxylate of **7** projects toward the neighboring polar region and engages a dynamic hydrogen-bonding network involving Thr446, Trp444, and Gln445. This interaction was also mediated by one or more bridging water molecules, while intermittent electrostatic and hydrogen-bond contacts with Arg547 further stabilize this polar interaction. Taken together, this MD-derived model supports the hypothesis that **7** interacts with the ACS active site by simultaneously exploiting a high-occupancy hydrophobic pocket and a precisely oriented para-anionic group that recruits a structured water-mediated polar network in the active site.

### The Antifungal Activity of 1,5-Diarylpyrazole ACS Inhibitors Correlates with On-Target Activity

We next determined the minimum inhibitory concentration (MIC) of the top four AR-12-derived ACS inhibitors against fungal strains of *C. albicans*, *C. neoformans*, and *A. fumigatus* (Table 5). It was immediately apparent that the carboxylic acid **7** and tetrazole **12** derivatives possessed the most potent antifungal activity, with MICs ranging from 32 to 16 μg/mL against most *C. albicans* and *C. neoformans* strains. This activity correlated well with the IC_50_ values against the CnAcs1 and CaAcs2; for example, the 37 μM value for **7** against CnAcs1 converts to 16 μg/mL, which is its MIC against *C. neoformans*. *C. albicans* clinical isolate 20385.058 was an exception, as only compound **12** possessed an MIC of 64 μg/mL. Similarly, the *C. neoformans* strains were most susceptible to **7** and **12**, while only 12 had activity against *A. fumigatus* CEA10 (Table 5; 64 μg/mL). It should be noted that both *C. neoformans* and *A. fumigatus* have ACS and ACL pathways for the synthesis of AcCoA. We have previously shown that CnAcs1-specific inhibitors inhibit the growth of *C. neoformans*; the modest activity of **12** against *A. fumigatus* is consistent with this observation.

**Table 5:** Antifungal Activity of Diarylpyrazole ACS Inhibitors

	MIC (μg/mL)			
Fungal Strain	**4**	**7**	**12**	**13**
*C. albicans* SN250	>64	32	32	>64
SN250 *cdr1*ΔΔ	>64	16	16	>64
*C. albicans* 20392.039	>64	32	32	>64
*C. albicans* 20385.058	>64	>64	64	>64
*C. neoformans* H99	32	16	16	32
*C. neoformans* 138.97	>64	16	16	>64
*C. neoformans* 103.98	64	16	32	>64
*C. neoformans* 111.00	>64	16	32	>64
*A. fumigatus* CEA10	>64	>64	64	>64

One of the most common mechanisms of antifungal resistance is mediated by putative drug efflux pumps.^25^ To determine whether this affects the antifungal activity of the 1,5-diaryl pyrazoles, we tested their activity against a *C. albicans* strain in which the efflux pump *CDR1* was deleted. The MIC of both **7** and **12** was reduced by 2-fold, which is the limit of detection of the assay. Therefore, it appears that efflux pump activity has a minor effect on the activity of this series of compounds, which is an important feature for a new class of antifungal compounds because drug efflux is a common mechanism of resistance.

### Diaryl Pyrazole **7** Has Reduced In Vitro Toxicity against HepG2 Cells and Improved Murine Pharmacologic Properties Relative to AR-12

One of the factors limiting therapeutic applications of AR-12 is its toxicity.^26,27^ In order to assess whether modifications to region A improve the physiological profile of the scaffold, **7**, **13**, and AR-12 were assessed for cytotoxicity by quantifying LDH release in HepG2 cells (Figure 3A). AR-12 caused maximal LDH release at 2 μg/mL, whereas **7** only produced the same effects at 64 μg/mL, and **13** never reached 100%, indicating substantially lower in vitro cytotoxicity relative to AR-12.

**Figure 3:**
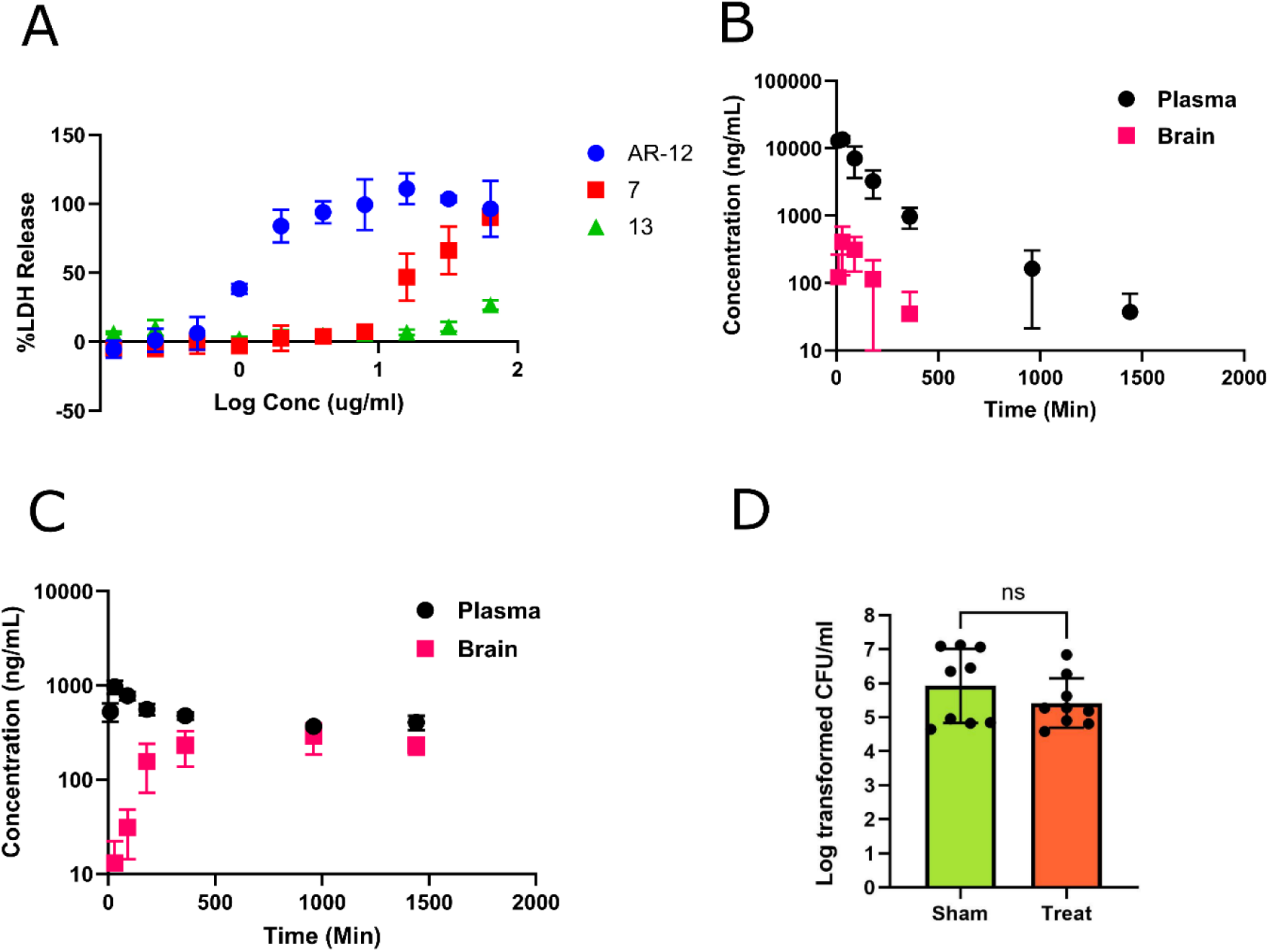
In vitro cytotoxicity, pharmacology, and in vivo activity of **7**. A. Hep2G cytotoxicity of **7**, **13**, and AR-12 (measured by LDH release; data points are means of three independent replicates with error bars showing standard deviation). B. Plasma and brain concentrations of **7** (B) and AR-12 (C) at the indicated time points following intraperitoneal administration of a 10 mg/kg dose to mice (data points are mean of three mice with error bars indicating standard deviation). D. Mice (*n* = 10/group) were infected with *C. albicans* strain SN250 by tail-vein injection and treated with 30 mg/kg of **7** or a sham for 2 days. Kidneys were harvested 72 h postinfection and fungal burden was determined by quantitative plating. Bars indicate the mean fungal burden with error bars showing the standard deviation. Differences between groups were analyzed by Student’s *t*-test with significance set at *p* < 0.05.

Next, we compared the pharmacological properties of **7** to those of AR-12 in a mouse model following IP injection of 10 mg/kg of each drug. Both brain and plasma concentrations were determined over a 1440 min period following injection. As shown in Figure 3B, **7** achieved good plasma exposure (Cmax: 13567 ng/mL) and an extended half-life (*T*_1/2_ = 230 min). However, very little brain exposure was observed (C_max_: 408 ng/mL) and the plasma/brain ratio at C_max_ was 0.028. The Cmax for **7** was >10-fold higher than for AR-12, while the relative brain exposure was much higher for AR-12 (C_max_: 290 ng/mL) with a plasma-to-brain ratio of 0.49 (Figure 3C). The half-life was much longer for AR-12. Finally, we tested the efficacy of **7** in a mouse model of disseminated *C. albicans* infection using a dose of 30 mg/kg per day because it would be predicted to establish a plasma concentration above the *C. albicans* in vitro MIC. However, we did not observe a statistically significant difference in fungal burden in the kidney (the target organ in mice) between sham and untreated mice (3 doses, Figure 3D).

## Discussion

ACS enzymes are candidate drug targets for the treatment of diseases ranging from fungal infections to cancer. Previous work identified ACS inhibition as a potential mechanism underlying the antifungal properties of AR-12, a Celecoxib derivative with broad antifungal activity against yeasts, molds, and dimorphic fungi.^15^ We undertook a medicinal chemistry-based optimization campaign centered on improving on-target activity against ACS enzymes from *C. albicans* and *C. neoformans*, two important human fungal pathogens. In doing so, we identified compounds with reduced toxicity and improved bioavailability relative to those of AR-12.

We previously reported that AR-12 inhibits *S. cerevisiae* ACS with an IC_50_ of 18 μM. In the current study, we focused on the *C. albicans* and *C. neoformans* ACS enzymes in hopes of identifying ACS inhibitors with greater clinical relevance. While inhibition of these enzymes was observed at high concentrations of AR-12, we were unable to obtain IC_50_ values due to overlap between inhibitory concentrations and the solubility point of AR-12. This observation supports the growing body of data suggesting that AR-12 has multiple targets contributing to its biological activity.^17,27^ Our initial goals were to use the AR-12 scaffold as a point of departure with the goals of: 1) identifying derivatives with improved on-target ACS potency against multiple fungal ACSs and 2) improving the toxicity and pharmacological properties of the diaryl-pyrazoles. Although we have not yet achieved candidates with in vivo activity, we have made substantial progress toward both of these goals.

Overall, our characterization of the ACS SAR for the AR-12 scaffold shows that the structural requirements for inhibition are quite specific for the two regions that we examined. Modification of region A showed that active compounds required functional groups with p*K*_a_ values similar to those of carboxylates at the 4-position of the phenyl ring at the 1-position of the pyrazole. The position of the acidic group is also important because the *meta*- and *para*-analogs of **7** have greatly reduced potency. The dependence of activity on the position of the negatively charged substituent suggests that the acidic group is participating in a specific molecular interaction rather than simply having a nonspecific physicochemical effect on potency. This is supported by the computational studies, which place the carboxylate of **7** in an orientation similar to the phosphate group of AMP. Movement of the carboxylate to the *meta* and *para* position would likely remove contact with Thr446, one of the primary contact residues based on MD. The parental compound, AR-12, contains a glycinamide at the key region A position (Figure 1). Regardless, **4**, **7**, **12**, and **13** are more potent than AR-12 toward the examined ACSs, indicating that increased negative charge at this position is important for improving ACS inhibition.

We observed an even more stringent SAR at region B, where only a phenanthrene ring was tolerated. An explanation for this can be surmised from our docking/MD simulation, where the B and C rings of phenanthrene participate in a π interaction with Tyr443. Initially, we were quite surprised that the biphenyl analog **17** possessed no activity relative to **7**. However, this derivative would lose planarity in region B, alongside the π interaction seen with the B ring of phenanthrene. Indeed, the docked pose of **17** placed the biphenyl in the phosphate-recognizing site and gave poor docking scores, indicating a large loss of complex stability. In the case of the other active AR-12 derivatives, we saw some variability in docking conformation, but all placed the phenanthrene ring in similar conformations, allowing for π interactions with Tyr443 (Figure S4).

Modifications to region B were mainly probed using a carboxylic acid (Table 2) or saccharin (Table 3) substituent or saccharin in region A. In the case of the latter, we did see some inhibition with the smaller hydrophobic substituents. A series of sulfonamide analogs with various ether-linked hydrophobic groups were also synthesized and tested (Table S2); however, none of these showed activity, mirroring the lack of inhibition for **1**. At a relatively superficial level, small aryl groups are not sufficient to engage with the enzyme. We elected not to explore even larger polyaromatic ring systems due to potential pharmacokinetic and toxicity issues that could arise. As such, it is possible that further optimization of region B may lead to more potent ACS inhibitors.

A notable feature of **7** compared to previously reported ACS inhibitors is its activity against multiple fungal ACSs and the human ACSS2 enzyme. In contrast, the human ACSS2 inhibitors VY-3-249 and MTB-9665 have shown no activity against CnAcs1.^20^ Similarly, a chemically distinct isoxazole series of CnAcs1 inhibitors is inactive against human ACSS2.^19^ Prior to this work, the only inhibitors with activity against fungal, parasitic, and human ACS enzymes have been derivatives of AMP that function as mimics of the acetyl-AMP intermediate of the reaction.^19,22^ To date, there has been no structure of the human enzyme (ACSS2), and therefore, the mechanism by which molecules such as VY-3-249 inhibit ACS is based on docking studies.

We were unable to obtain the crystal structure of the AR-12 derivatives bound to CnAcs1 or CaAcs2. Therefore, we sought a computational approach to detail a potential binding site. Since we observed broad inhibition of diverse ACS enzymes, we hypothesized that the binding site may mimic that of AMP esters. To determine whether estimates of binding affinity for this site align with enzyme inhibition data, we performed ROC analysis of our docking results. Applying ROC analysis to docking is one of the most rigorous ways to quantify docking accuracy, and we obtained a highly favorable AUC of 0.93 for the ROC curve. Much to our satisfaction, the docking protocol estimated improved binding affinity for the active compounds, suggesting that the selected binding site may represent the true target of the AR-12 derivatives. In further support of this, docking into an adjacent pocket did not provide a favorable ROC curve and docking score for the actives. An MD simulation of **7** based on the docked pose showed stable interactions with the docking site residues, which also incorporated bridging water molecules in the dynamic system.

The interaction of this series of molecules with the ATP or acetyl-AMP binding pocket of the active site is also supported by biochemical data indicating that AR-12 ^15^ and **12** are competitive with ATP (Figure S3). However, it is important to keep in mind that the ACS is a multisubstrate enzyme that undergoes large conformational changes during the two-step reaction.^19^ Therefore, it is likely that the exact biochemical mechanism of inhibition for these molecules is more complex than simple lock-and-key binding.

With those caveats, the ATP pocket binding of the diaryl-pyrazoles is also supported by other efforts to develop ACS inhibitors. For example, the only previously known broad-spectrum ACS inhibitors are derivatives of AMP, with multiple crystal structures showing occupancy in the ATP pocket. Additionally, we recently reported a highly selective CnAcs1 inhibitor that bound in the CoA and acetate binding sites, as confirmed from X-ray crystallography.^19^ As part of that work, we examined an antimalarial ACS inhibitor that similarly targeted the CoA pocket and showed preferential inhibition for *Plasmodium falciparum* ACS. Taken together, our work supports the hypothesis that molecules which bind in the ATP pocket lead to broader-spectrum ACS inhibition, while binding in the CoA/acetate sites correlates with greater selectivity.

Further encouragement for the continued development of ACS as an antifungal target is found in the antifungal activity of compounds **7** and **12**. Although the activity is modest, it correlates well with the potency of the inhibitors against the ACS enzymes. This supports the conclusion that the antifungal activity is due to on-target inhibition. Notably, we observed antifungal activity against *C. neoformans*. *C. neoformans* has both ACS and ACL enzymes and, thus, deletion mutants of Cn*ACS1* are viable. We have previously shown that the isoxazole class inhibitors of CnAcs1 also have antifungal activity against *C. neoformans*.^19^ We hypothesized that this is due to the increased dependence of *C. neoformans* on CnAcs1 in relatively nutrient-poor medium such as tissue culture medium,^19,23^ or, alternatively, is due to the fact that acute inhibition of CnAcs1 does not allow the cell to reprogram its metabolic state to compensate as it does during the creation of a genetic mutant. The observation of antifungal activity with a chemically distinct class of CnAcs1 inhibitors further supports the notion that the inhibition of ACS enzymes in fungi that can also generate acetyl-CoA through ACL could still be an effective antifungal strategy.

Finally, we have addressed two additional liabilities associated with AR-12 by reducing the *in vitro* cytotoxicity and improving bioavailability. Since we have not yet achieved a molecule with efficacy in animal models of fungal infection, additional optimization of the aryl-pyrazole ACS inhibitors will be needed. However, the insights gained from structural modeling of the interaction of this series with the target enzyme will facilitate optimization. Interest in ACS inhibitors as therapeutic leads has been increasing across a number of areas of medicine and our data support the potential of this target for future development in the antifungal space.

## Materials and Methods

### General Chemical Materials and Methods

All starting materials for chemical synthesis were purchased from either Aaron Chemicals, AmBeed Inc., or Enamine Ltd. and were used without further purification. All compounds have a purity of >95% as judged by HPLC analysis (UV absorbance at 254 nm). A full description of the chemical synthesis can be found in the Supporting Information.

### General Microbiological Methods and Strains

*C. albicans*, *C. glabrata*, *C. neoformans*, and *A. fumigatus* strains have been published previously.^28,29^ Media were prepared using standard recipes.^29^ Strains were precultured overnight at 30 °C in yeast peptone dextrose medium prior to use in susceptibility assays and in vivo animal experiments.

### Expression and Purification of Acyl-CoA Synthetases

The expression, purification, and characterization of these enzymes have been previously reported by our lab.^19,20^ Briefly, expression plasmids for the ACS enzymes were transformed into the *Escherichia coli* strain BL21 with appropriate antibiotic selection. Resistant colonies were used to start an overnight culture in standard LB broth with continued antibiotic selection, shaking at 200 rpm and 37 °C. The following morning, bacterial overnights were inoculated with a 1:1000 dilution of each respective overnight culture and allowed to grow while shaking at 200 rpm and 37 °C until mid-log phase (OD_600_ 0.5–0.8), then induced with 1 mM isopropyl-β-D-thiogalactopyranoside for 2 h. Pelleted cells were lysed via sonication and protein was purified via IMAC. All proteins retained their 6X-his tag following purification and were stored at −80 °C in elution buffer.

### ACS Enzymatic Assays

Initial screening of ACS inhibition by compounds was performed in duplicate wells on three separate days. Compound stocks were created at 50 μM and 25 μM, in 50% DMSO. The enzyme reactions were performed as previously described.^15^ Briefly, the enzyme reaction mixture contained 125 mM K_2_PO_4_, 4 mM MgCl_2_, 50 mM KF, 10 mM GSH, 1 mM CoA, 1 mM ATP, 0.5 mM acetate, and 200 mM hydroxylamine. The assays were incubated for 50 min at 37 °C, at which time the quenching reagent (616 mM FeCl_3_ and 180 mM trichloroacetate) was added, and absorbance at 540 nM was measured using a SpectraMax i3X Multi-Mode plate reader (Molecular Devices). Dose-response curves were generated using a minimum 8-point 2-fold drug dilution series, along with a DMSO and ethyl-AMP control. The 50% inhibitory concentration (IC_50_) was calculated using the nonlinear regression analysis software, Prism (GraphPad). All inhibition studies were performed with a minimum of three experimental replicates and inclusion of a positive control inhibitor, the competitive inhibitor ethyl-AMP at 50 μM.

For compounds with high background at 540 nM, ACS activity was determined using the absorption-based coupled kinetic assay as previously described.^19^ Substrate and coupling reagents were prepared either fresh or thawed from small aliquots stored at −80 °C for a maximum of two freeze–thaw cycles. All tested drug concentrations were diluted from the stock such that DMSO concentrations did not exceed 5%. All reagents, including the compound and minus the start reagent, were mixed and aliquoted at room temperature, followed by a 15 min incubation at 37 °C. Acetate was then added to start the coupled reaction, and the reaction was followed continuously at 37 °C in a SpectraMax i3X Multi-Mode plate reader (Molecular Devices) at an absorbance of 360 nm.

### Molecular Docking Calculations

The ligand structures were initially sketched in ChemDraw Ultra 12.0 and then converted to three-dimensional geometries using ChemDraw Ultra 12.0. Docking studies were carried out in Maestro (version 13.9.138, Schrödinger Release 2024-1). Ligands were processed with LigPrep under the OPLS4 force field, during which desalted forms, relevant ionization and tautomeric states, and physiologically reasonable protonation states at pH 7.0 ± 2.0 were generated using Epik. The CaAcs2 protein structure (PDB: 8V4P) was prepared in Maestro’s Protein Preparation Wizard using the protocol described by Madhavi Sastry and colleagues.^30^ This included assigning bond orders, adding missing hydrogens and incomplete side chains, optimizing protonation states at pH 7.0, and removing crystallographic waters located more than 6 Å from the heteroatom groups. The protein was subsequently minimized using the OPLS4 force field. A receptor grid was then generated by centering the 23 Å box on the AMP-ester binding region. Docking employed the receptor grid (.zip) and the processed ligand library, with Glide operating in Extra Precision (XP) mode.^31^ During docking, ligand flexibility was fully sampled, while the receptor remained rigid, aside from adjustments within the active site.

### Molecular Dynamics Simulations

The docked complex of compound **7** in 8V4P was subjected to molecular dynamics (MD) simulations using the Desmond module in the Schrödinger software suite (Schrödinger, LLC, New York, NY, Release 2024-1), employing the OPLS5 force field.^32^ The system was solvated using the simple point charge (SPC) water model, and the simulation boundary was defined by placing the complex in an orthorhombic water box with a total volume of 1,572,935 Å^3^, generated using the Buffer method for box-size determination. The system was then neutralized with sodium ions to balance the net charge. A 1000 ns production MD simulation was performed under an isothermal–isobaric (NPT) ensemble at 300 K and 1.01325 bar using Nosé–Hoover temperature coupling and isotropic pressure scaling. System equilibration and subsequent production dynamics followed the standard Desmond protocol. Trajectory analyses included RMSD, RMSF, and ligand–protein interaction profiling, with all simulation parameters summarized in Table S3.^33^

### In Vitro Mammalian Cytotoxicity Assay

HepG2 (ATCC, HB-8065) cells were maintained in Dulbecco’s Modified Eagle Medium (Gibco) supplemented with 10% fetal bovine serum and 1% penicillin/streptomycin. Cells were cultured at 37 °C in a humidified atmosphere with 5% of the CO_2_. To perform toxicity assays, cells were seeded into 96-well plates at a density of 1.25 × 10^4^ cells per well and incubated overnight. The following day, the medium was aspirated and replaced with fresh medium containing a 2-fold dilution series of the test drug, with an equal concentration of DMSO in all wells. After 24 h of incubation, the supernatant was collected to quantify lactate dehydrogenase (LDH) release using the CyQuant LDH assay kit (Invitrogen).

### Antifungal Susceptibility Assays

The susceptibility of the fungal strains to the ACS inhibitors was determined using a modified Clinical and Laboratory Standards Institute (CLSI) microdilution method as previously described.^19^ Overnight cultures of *C. albicans* and *C. neoformans* were washed in sterile phosphate-buffered saline (PBS) and brought up into RPMI supplemented with 165 mM MOPS pH 7.0, such that 1000 cells would be delivered into each well with a final volume of 200 μL in a 96-well plate. Each tested drug was added so that the highest concentration was 64 μg/mL and 1.25% DMSO. Plates were incubated at 37 °C for 72 h for *C. neoformans* and 24 h for *C. albicans* and *A. fumigatus*. Each assay was performed in a minimum of technical duplicates with three independent experimental replicates

### Pharmacological Analysis of AR-12 and **7** in Mice

Twenty-one female CD-1 mice were dosed with IP with 10 mg/kg of **7** or AR-12. Whole blood was collected in a syringe coated with ACD. Plasma was processed from whole blood by centrifugation at 10,000 rpm for 10 min. Brain tissue was harvested and was gently washed with 1x PBS to remove residual circulating blood. The tissue was weighed and snap frozen in liquid nitrogen. **Plasma Processing**: For the standards and quality controls, 98 μL and 98.8 μL of blank vendor plasma were added to a microfuge tube and spiked with 2 and 1.2 μL of the initial standard. Standards, quality controls, and samples of 100 μL were then precipitated with 200 μL of methanol containing 0.15% formic acid and 12.5 ng/mL IS (final concentration). The samples were vortexed for 15 s, incubated at room temperature for 10 min, and centrifuged at 13,200 rpm twice in a standard microcentrifuge. The supernatant was then analyzed by LC-MS/MS. Vendor-supplied plasma used in standards and QCs: Bioreclamation, LLC; lot #MSE284936; ACD anticoagulant. **Brain Processing:** Brain tissues were homogenized in 3X volume of PBS (X = weight of the tissue). For the standards and quality control samples, 98 μL and 98.8 μL of pooled blank brain were added to a microfuge tube and spiked with 2 or 1.2 μL of the initial standard. Standards and quality control samples of 100 μL were then precipitated with 200 μL of methanol containing 0.15% formic acid and 12.5 ng/mL IS (final concentrations). The samples were vortexed for 15 s, incubated at room temperature for 10 min, and centrifuged at 13,200 rpm twice in a standard microcentrifuge. The supernatant was then analyzed by LC-MS/MS.

### Mouse Model of Disseminated Candidiasis

CD1 female mice (10 per group, 6–8 weeks old; Envigo) were inoculated by lateral tail vein injection with 5 × 10^5^ *C. albicans* SN250. Mice were injected immediately after infection and every 24 h with 30 mg/kg **7**, formulated in 5% DMSO/20% PEG400/1% Tween 80/74% D5W. Mice were monitored daily, and those who demonstrated symptoms of severe diseases such as fur ruffling, abnormal posture, and/or failure to respond to the surroundings were euthanized immediately. Kidneys were harvested after 72 h postinfection and collected into ice-cold sterile phosphate-buffered saline. 10 μL of 10-fold serial dilutions of the extracts were spotted onto the YPD plates, and fungal burden was determined on the log-transformed values using unpaired *t*-test. Mouse experiments were approved by the University of Iowa Institutional Animal Care and Use Committee and were performed in accordance with all national and local guidelines.

## Supplementary Material



## Notes

Supporting table, figures, and chemical structure validation can be found in the Supporting Information; Synthetic techniques can be found in the experimental SI (PDF)

## Abbreviations

ACS: acetyl-CoA Synthetase
CoA: coenzyme A
AcCoA: acetyl-coenzyme A
ACL: ATP-citrate lyase
DMC: 2,5-dimethylcelecoxib
SAR: structure–activity relationship
CnAcs1: *Cryptococcus neoformans* ACS1
CaAcs2: *Candida albicans* ACS2
DMF: dimethylformamide
THF: tetrahydrofuran
ACSS2: *Homo sapiens* ACS
AfAcs1: *Aspergillus fumigatus* ACS
CiAcs1: *Coccidioides immitis* ACS
ScAcs1: *Saccharomyces cerevisiae* ACS
CnKbc1: *Cryptococcus neoformans* aceto-acetyl-CoA synthetase
MIC: minimum inhibitor concentration
AMP: adenosine monophosphate
