# Supplementary material for: Optimization of Antifungal 1,5-Diaryl-Pyrazole Acetyl-CoA Synthetase Inhibitors
Source: ACS Omega. 2026 Apr 14;11(16):24237–49. doi: 10.1021/acsomega.5c13157 (PMC13130119; doi:10.1021/acsomega.5c13157)

Optimization of antifungal 1,5-diaryl-pyrazole acetyl-CoA synthetase inhibitors

Supplemental Data

Jonah P. Propp[1], Jeffrey C. Ferreira[2], Parisa Enayati[2], Kathryn M. Alden[1], Drashti G. Daraji[2], Charles L. Lail III[2], Michael E. Heene[2], Andrew J. Jezewski[1], Rohan Wakade[1], Damien Castor[1], Noelle S. Williams[3], Timothy J. Hagen[2]*, and Damian J. Krysan*[1,8]

[1]Department of Pediatrics, Carver College of Medicine, University of Iowa, Iowa City, IA 52242, USA. [2]Department of Chemistry and Biochemistry, Northern Illinois University, DeKalb, IL 60115, USA. [3]Department of Biochemistry, UT Southwestern Medical Center, Dallas, TX 75390, USA. [4]Department of Molecular Physiology and Biophysics, Carver College of Medicine, University of Iowa, Iowa City, IA 52242, USA.

Figure S1. IC_{50} curves of top AR-12 derivatives against ACS enzymes

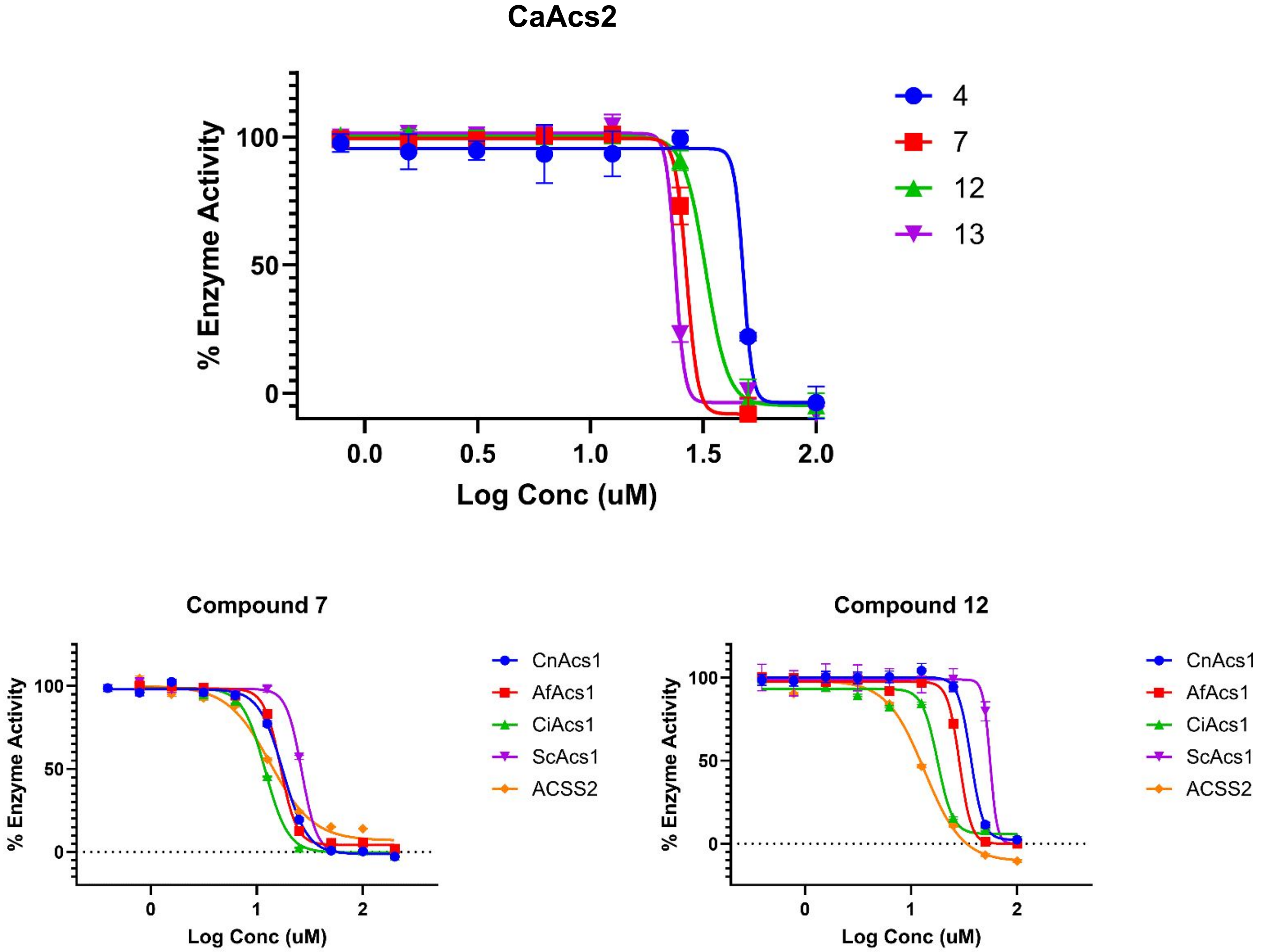

Representative inhibition data for top inhibitors using the continuous, enzyme-coupled assay described in the methods. Compounds **7** and **12** were assayed against various ACS enzymes, with IC_{50} values reported in Table **4**. IC_{50} curves were also examined using the end-point iron complex assay, but the compounds gave high background at top concentrations (data not included).

Table S1. Docking scores and ROC tabulation

Ligand Name	SubPocket score	Cumulative Actives	Cumulative Total	Cumulative Inactives	True Positive Rate	False Positive Rate
4	-7.589	1	1	0	0.25	0
7	-7.129	2	2	0	0.5	0
1	-5.749	3	3	0	0.75	0
12	-5.354	4	4	0	1	0
6	-4.575	4	5	1	1	0.056
9	-4.455	4	6	2	1	0.111
17	-4.162	4	7	3	1	0.167
10	-4.145	4	8	4	1	0.222
22	-3.888	4	9	5	1	0.278
21	-3.75	4	10	6	1	0.333
20	-3.746	4	11	7	1	0.389
23	-3.614	4	12	8	1	0.444
5	-3.516	4	13	9	1	0.5
24	-3.466	4	14	10	1	0.556
15	-3.367	4	15	11	1	0.611
3	-3.264	4	16	12	1	0.667
14	-3.256	4	17	13	1	0.722

Figure S2. ROC curve for docking into 8V4P Chain C

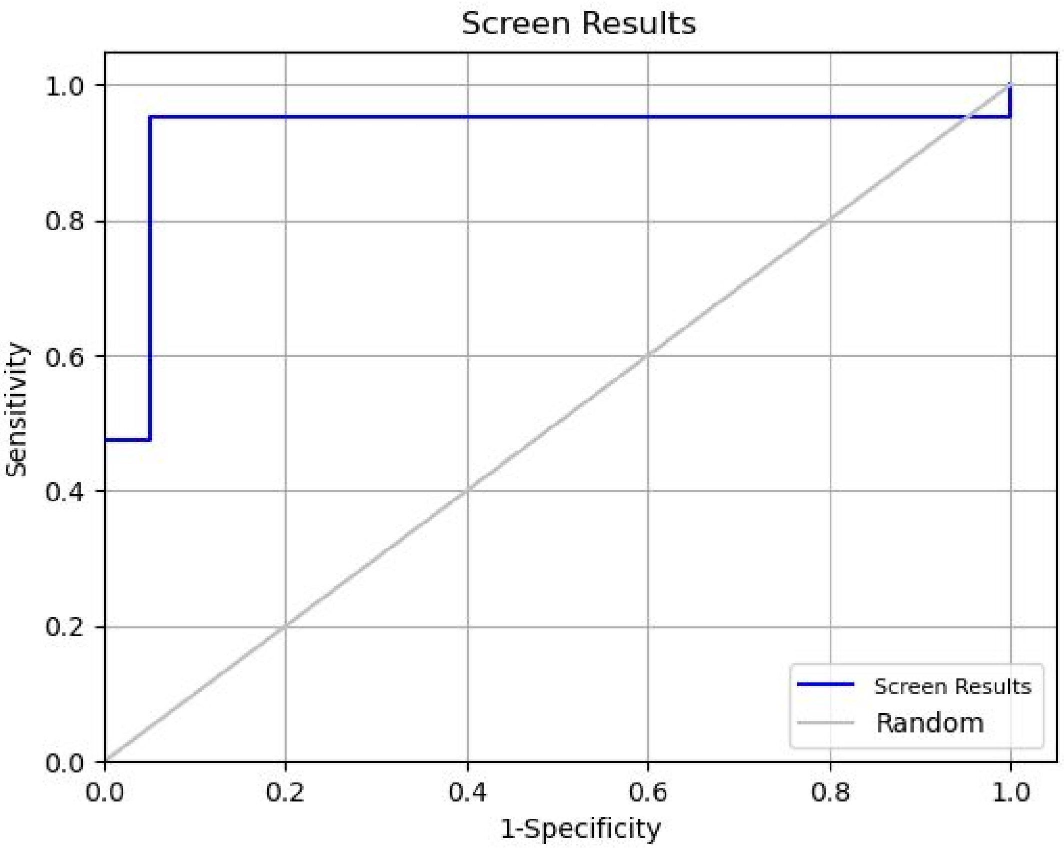

ROC curve of AR-12 scaffold with **4, 7,** and **12** designated as actives, and **1, 2, 5, 6, 9-11, 14-17,** and **19-24** designated as inactive. Sensitivity is the true positive rate (TPR), defined as the number of true positives / (true positives + false negatives), and 1-specificity is the false positive rate (FPR), defined as false positives / (true negatives + false positives). The docking data used to generate the TPR and FPR are listed in **Table S1**. Docking was performed using Glide extra precision mode and provided an AUC of 0.93.

Figure S3. Compound **12** shows competitive inhibition with respect to ATP

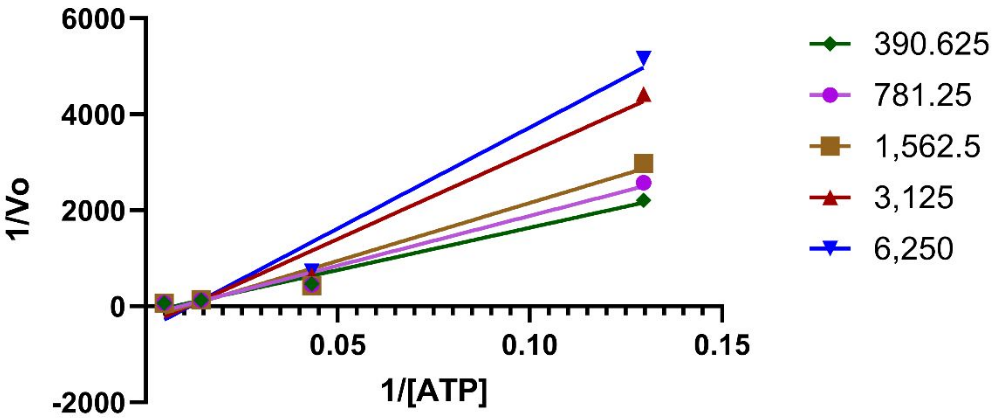

Lineweaver-Burke plot shows changes in initial velocity (V_o) at varying substrate concentrations of ATP. While interpretation of data is hindered by a steep drop-off in enzyme activity at high inhibitor concentrations, increasing concentrations of ATP reduce inhibition caused by compound **12**, as indicated by intersecting lines at the X-axis, and lower initial rate at high substrate concentrations.

Figure S4. Docking pose overlay of actives used in ROC analysis

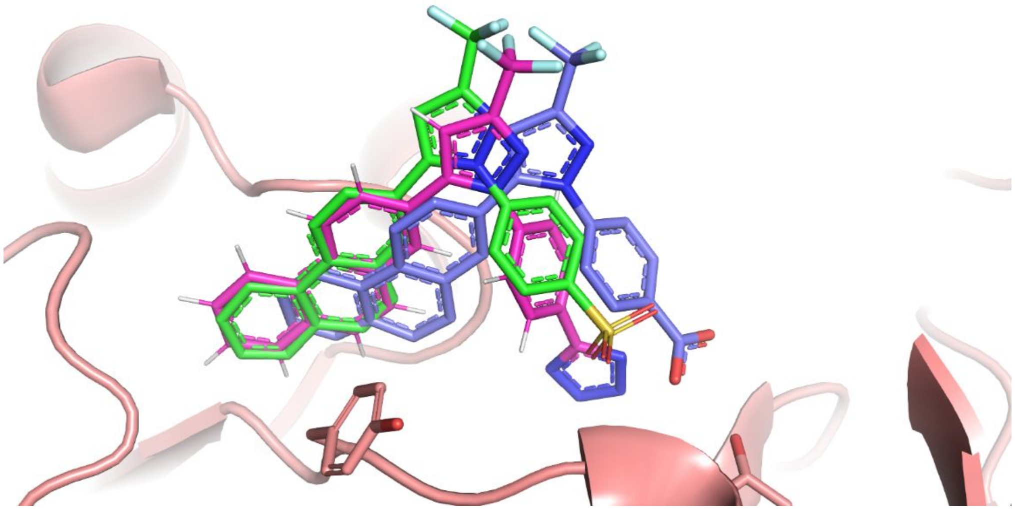

Overlay of **4, 7,** and **12**, within the CaAcs2 ATP pocket. While some variations in region A and B are observed, they largely agree in terms of placing the phenanthrene near Tyr443/the adenine binding region and the negative charge in region B near the phosphate group of AMP.

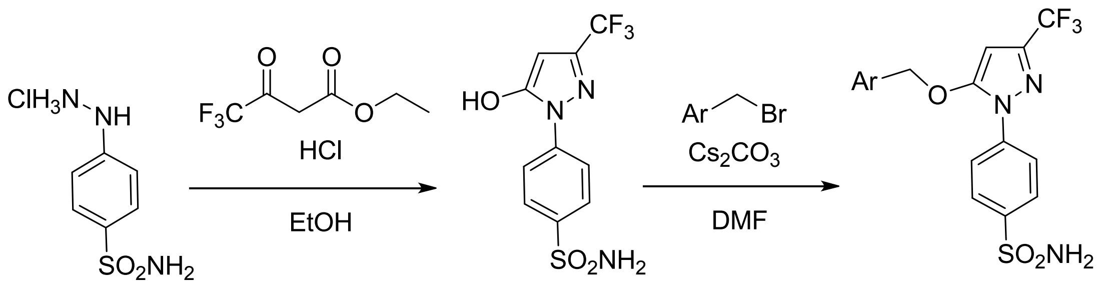

(**28a**)

Ar = 4-C_6H_4Cl (**28**)
Ar = 4-C_6H_4F (**29**)
Ar = 4-$C_6H_4CH(CH_3)_2$ (**30**)
Ar = 4-$C_6H_4CH_3$ (**31**)
Ar = 4-$C_6H_4C(CH_3)_3$ (**32**)
Ar = 4-CH_2CH_3 (**33**)
Ar = C_6H_5 (**34**)
Ar = 2-naphthyl (**35**)
Ar = 1-naphthyl (**36**)

Figure S5: Synthetic Route for Sulfonamide Analogs (**28-36**).

The synthesis of sulfonamide analogs (**Scheme S1**) followed a similar pathway to that of **Scheme 2B**. Incorporation of an oxygen atom at the 5-position allowed for rapid functionalization of the intermediate species (**28a**), which was synthesized from a Knorr pyrazole synthesis utilizing ethyl trifluoroacetoacetate. This process afforded the 5-hydroxy-pyrazole rather than the 5-ethoxy-pyrazole. The -OH group of this intermediate compound was successfully alkylated, and not the sulfonamide group, by utilizing various benzyl bromides with Cs_2CO_3 in DMF at room temperature to form compounds **28-36** in fair to good yields.

Table S2. SAR of sulfonamide AR-12 analogs

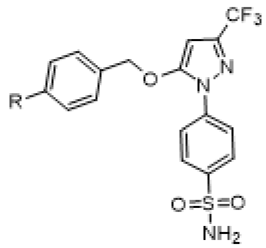

	R	CnAcs1 50μM	CnAcs1 25μM	CaAcs2 50μM	CaAcs2 25μM
28	Cl	0%	0%	7.4% ± 1.4	0%
29	F	0%	0%	0%	0%
30	Isopropyl	0%	0%	0%	4.8% ± 4.5
31	Methyl	0%	0%	5.5% ± 0.4	3.3% ± 7.1
32	Tert-butyl	0%	0%	0%	1.4% ± 1.7
33	Ethyl	0%	0%	4.6% ± 3.6	0%
34	H	0%	0%	6.4% ± 1.3	1.0% ± 2.0
35	2-Naphthelenyl	0%	0%	0%	0%
36	1-Naphthelenyl	0%	0%	0%	0%

Table S3. MD simulation summary

Parameter	Value
Simulation time	1001.002 ns
Ensemble	NPT (300 K)
Total No of atoms	49300
Number of water molecules	13714
Ions added	14 Na+ (18.561 mM)
Protein	523 residues, Chain C, 4112 Heavy Atome
Ligand	$C_{25}H_{14}F_3N_2O_2$,46 atoms, Charge (-1)
Box Dimensions	Cubic Box (3A°)

Figure S6: Structural characterization of synthesized compounds

A: 4-(5-(phenanthren-2-yl)-3-(trifluoromethyl)-1H-pyrazol-1-yl)benzenesulfonamide (1)

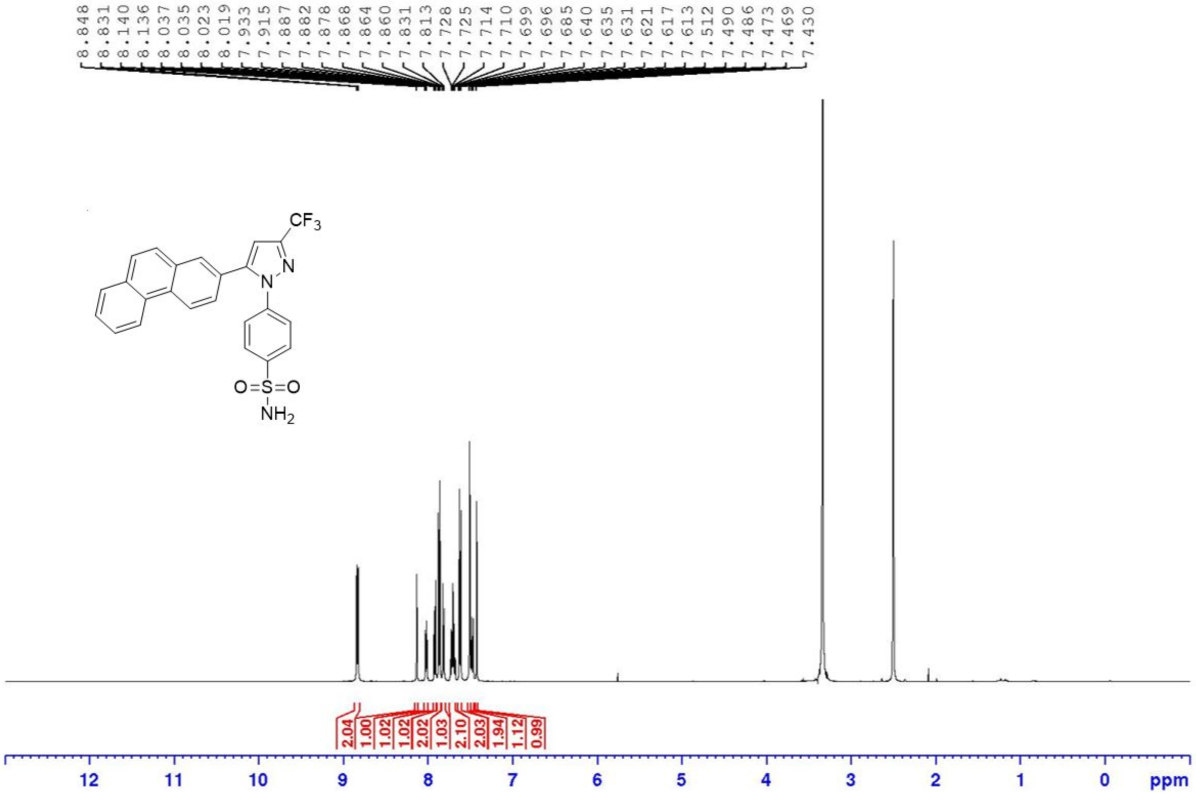

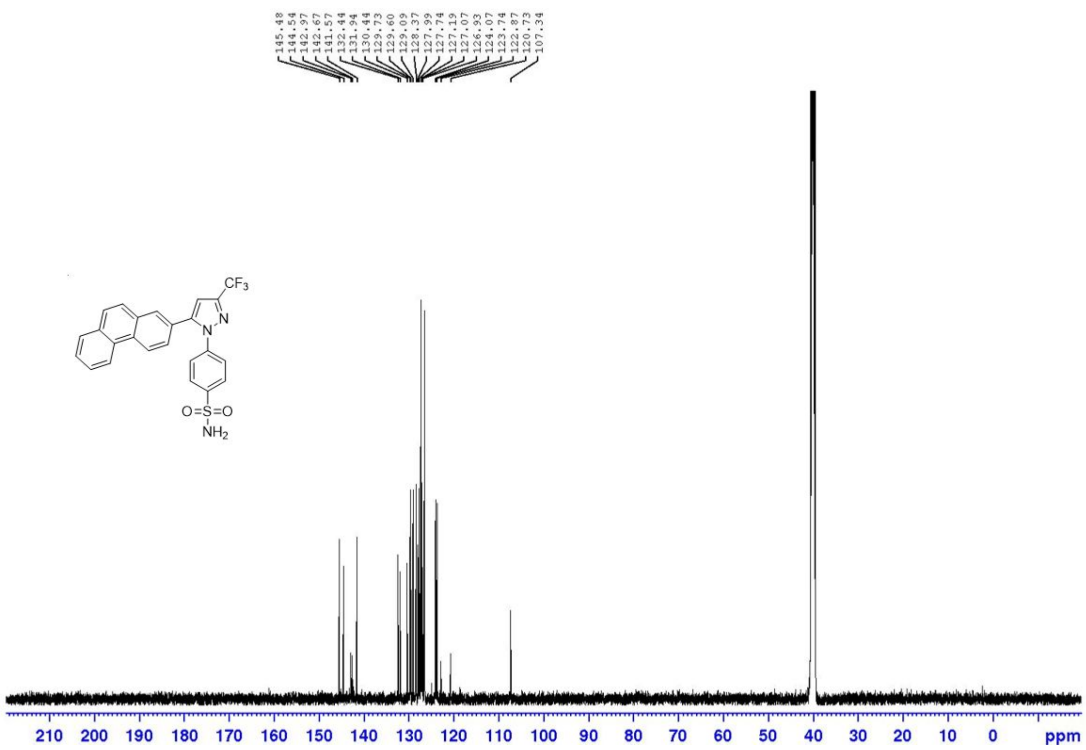

B: N-((4-(5-(phenanthren-2-yl)-3-(trifluoromethyl)-1H-pyrazol-1-yl)phenyl)sulfonyl)acetamide (2)

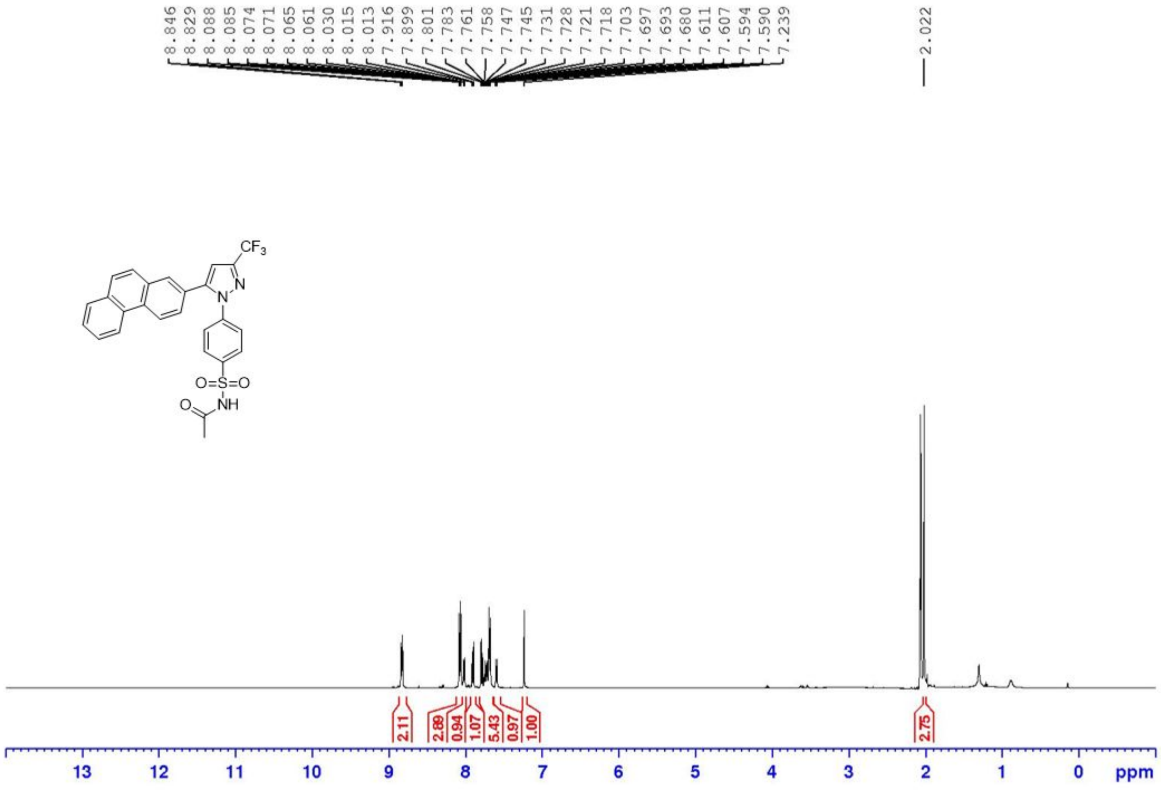

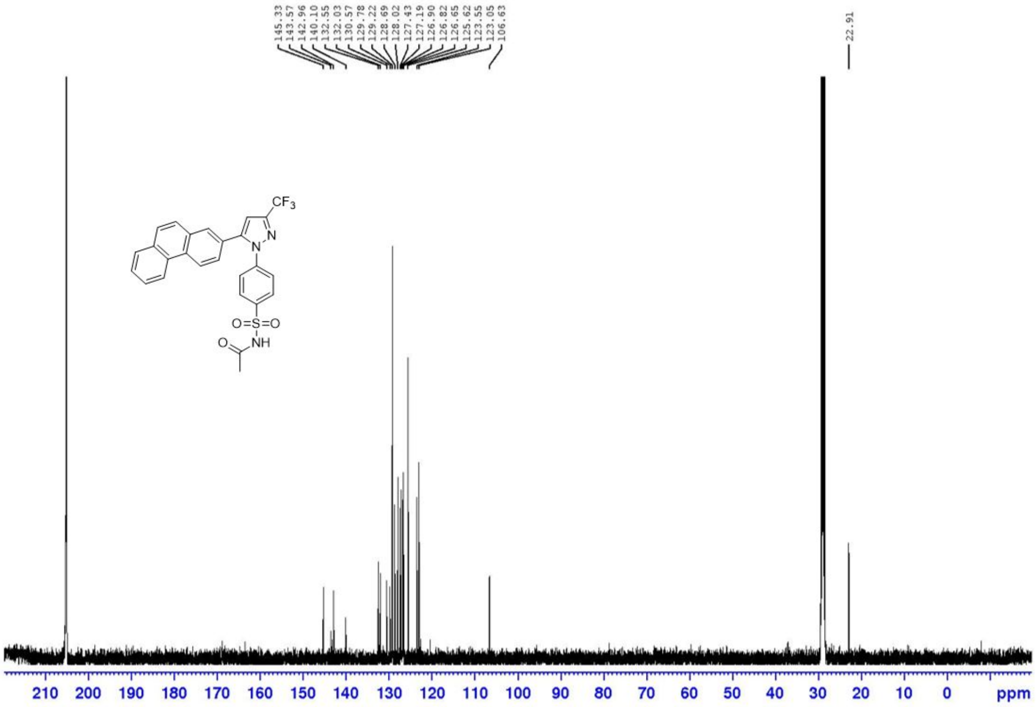

C: N-ethyl-4-(5-(phenanthren-2-yl)-3-(trifluoromethyl)-1H-pyrazol-1-yl)benzenesulfonamide (3)

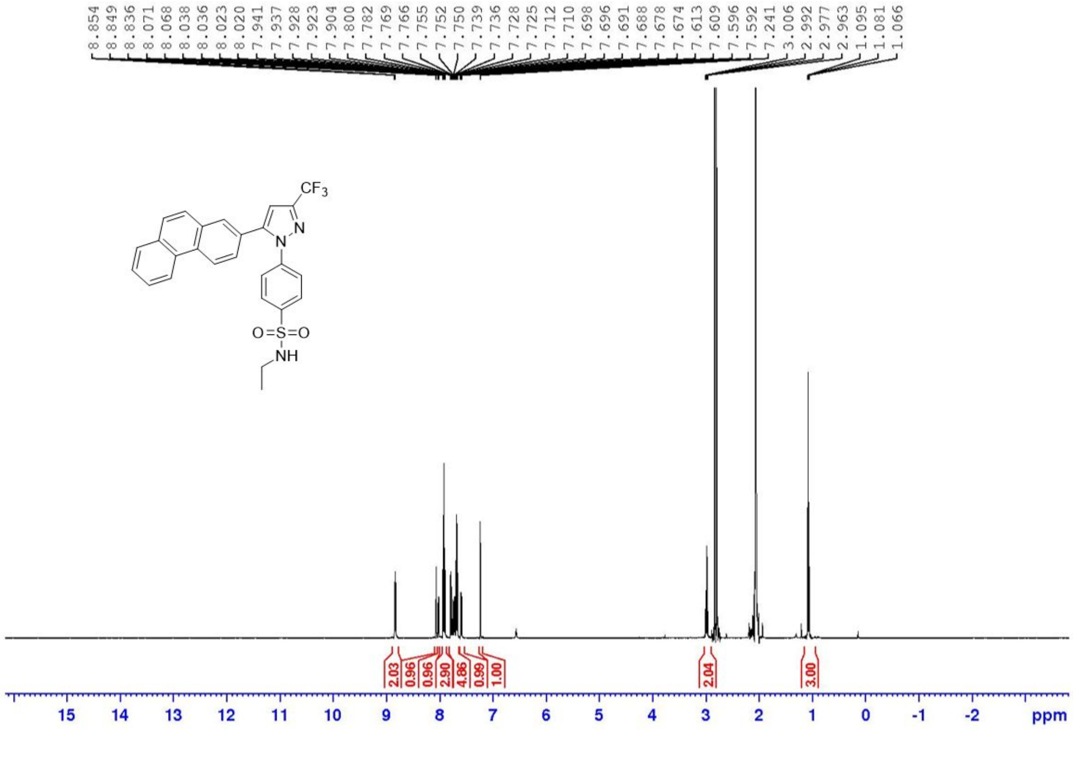

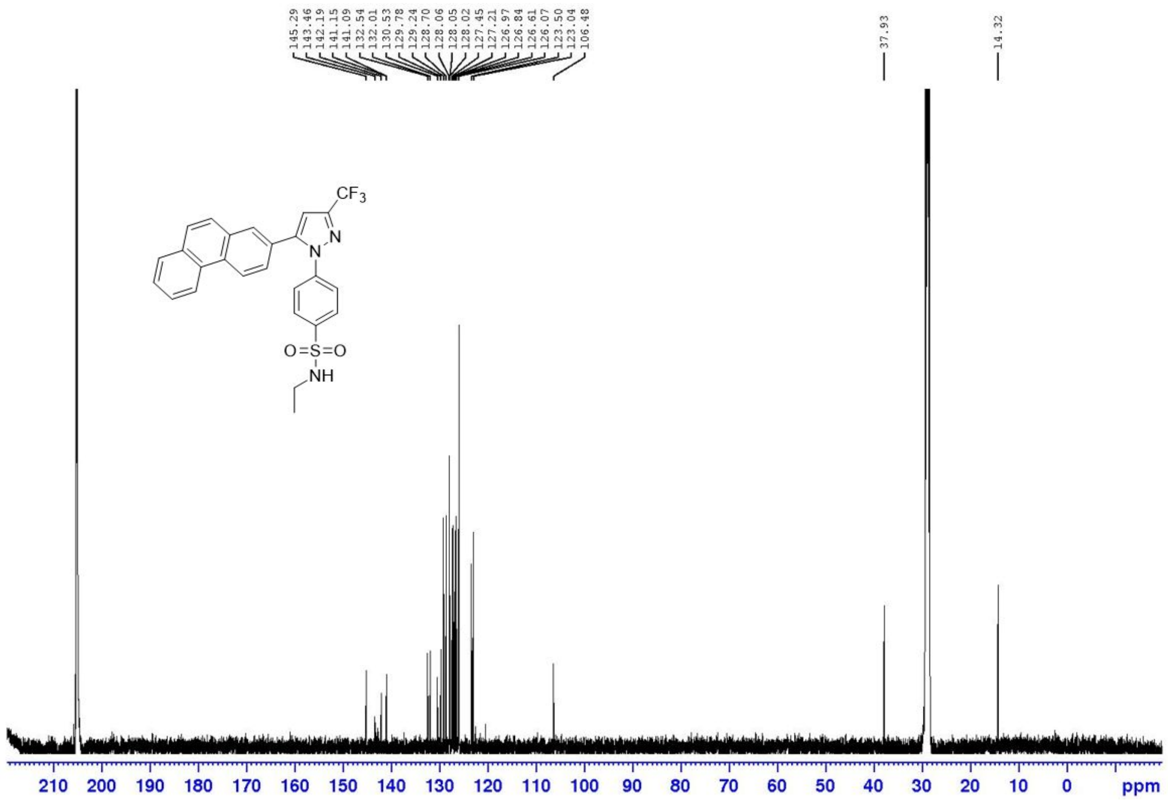

D: 4-(5-(phenanthren-2-yl)-3-(trifluoromethyl)-1H-pyrazol-1-yl)benzenesulfonic acid (4)

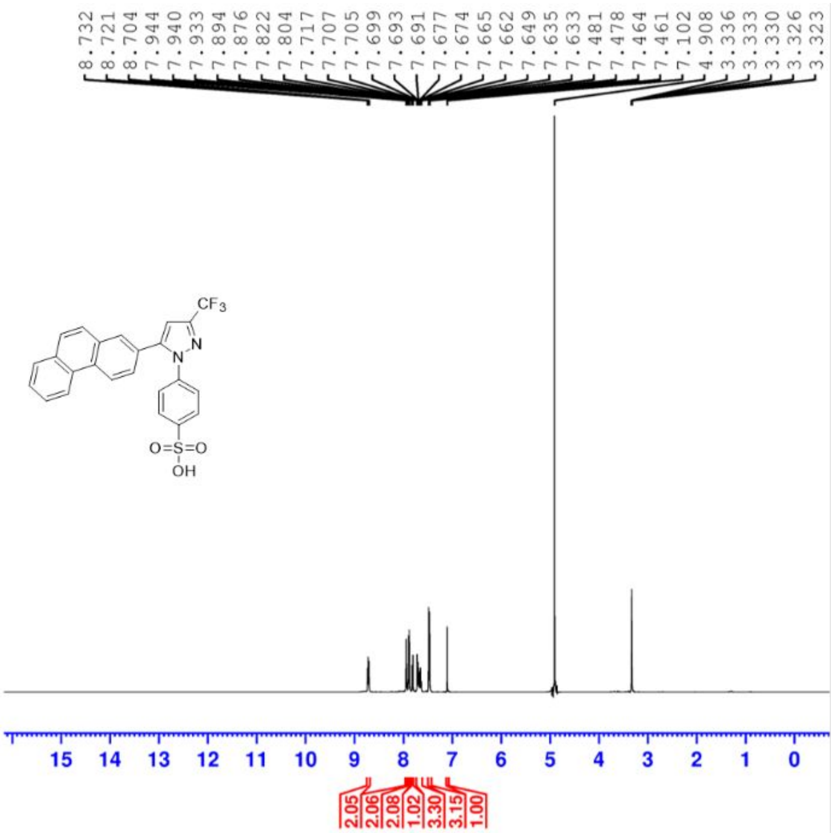
8.732
8.721
8.704
7.944
7.940
7.933
7.894
7.876
7.822
7.804
7.717
7.707
7.705
7.699
7.693
7.691
7.677
7.674
7.665
7.662
7.649
7.635
7.633
7.481
7.478
7.464
7.461
7.102
4.908
3.336
3.333
3.330
3.326
3.323
CF3
N
N
O=S=O
OH
15 14 13 12 11 10 9 8 7 6 5 4 3 2 1 0
2.05
2.06
2.08
1.02
3.30
3.15
1.00

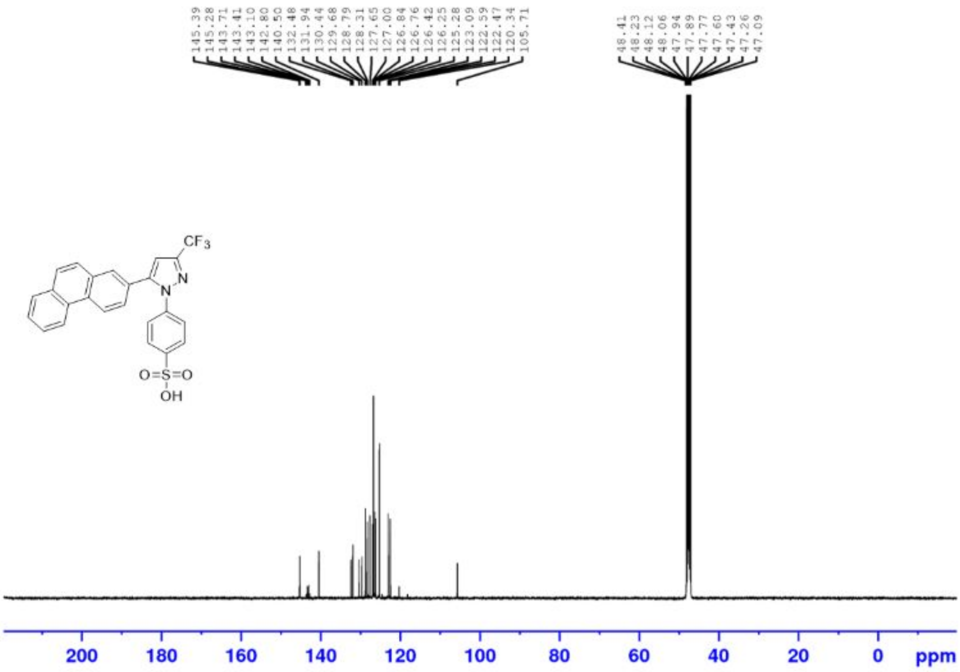
145.39
145.28
143.71
143.41
143.10
142.80
140.50
132.48
131.94
130.44
129.68
128.79
128.31
127.65
127.00
126.84
126.76
126.42
126.25
125.28
123.09
122.59
122.47
120.34
105.71
48.41
48.23
48.12
48.06
47.94
47.89
47.77
47.60
47.43
47.26
47.09
CF3
N
N
O=S=O
OH
200 180 160 140 120 100 80 60 40 20 0 ppm

E: 4-(5-(phenanthren-2-yl)-3-(trifluoromethyl)-1H-pyrazol-1-yl)-N-(thiazol-2-yl)benzenesulfonamide (5)

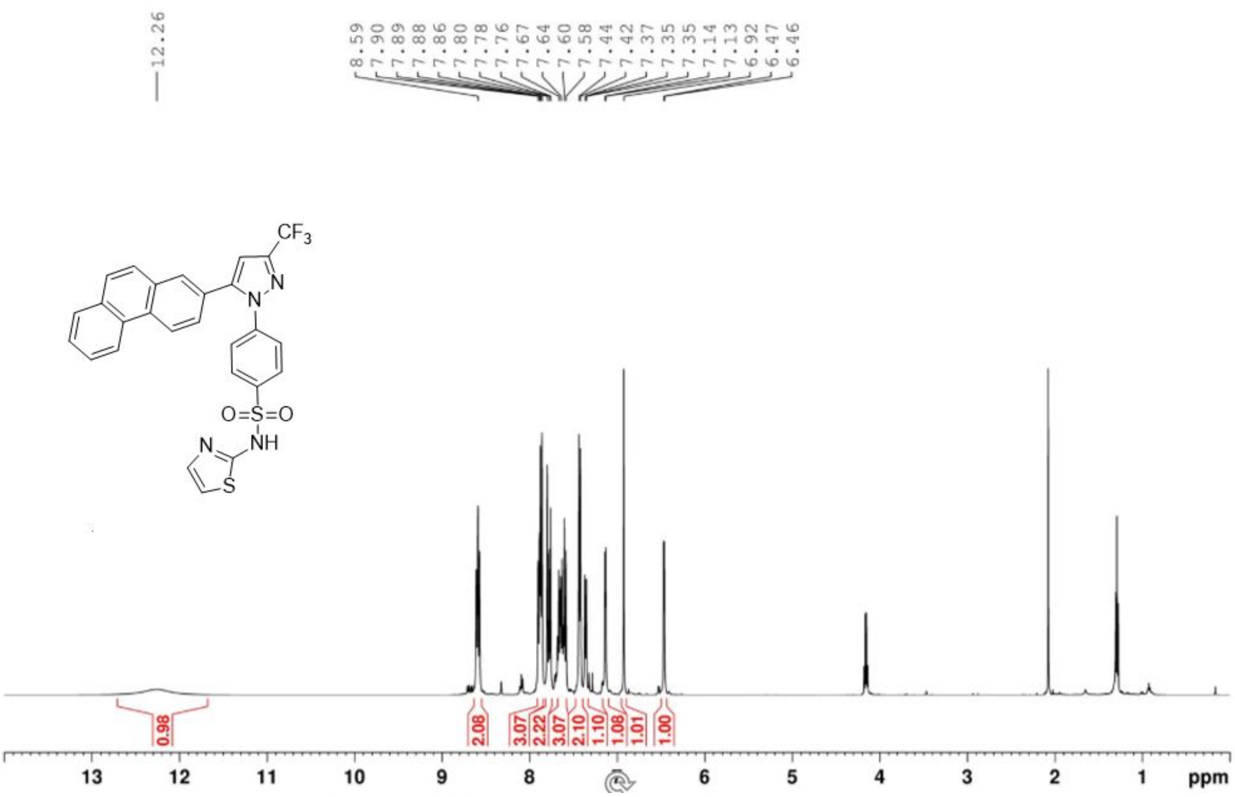

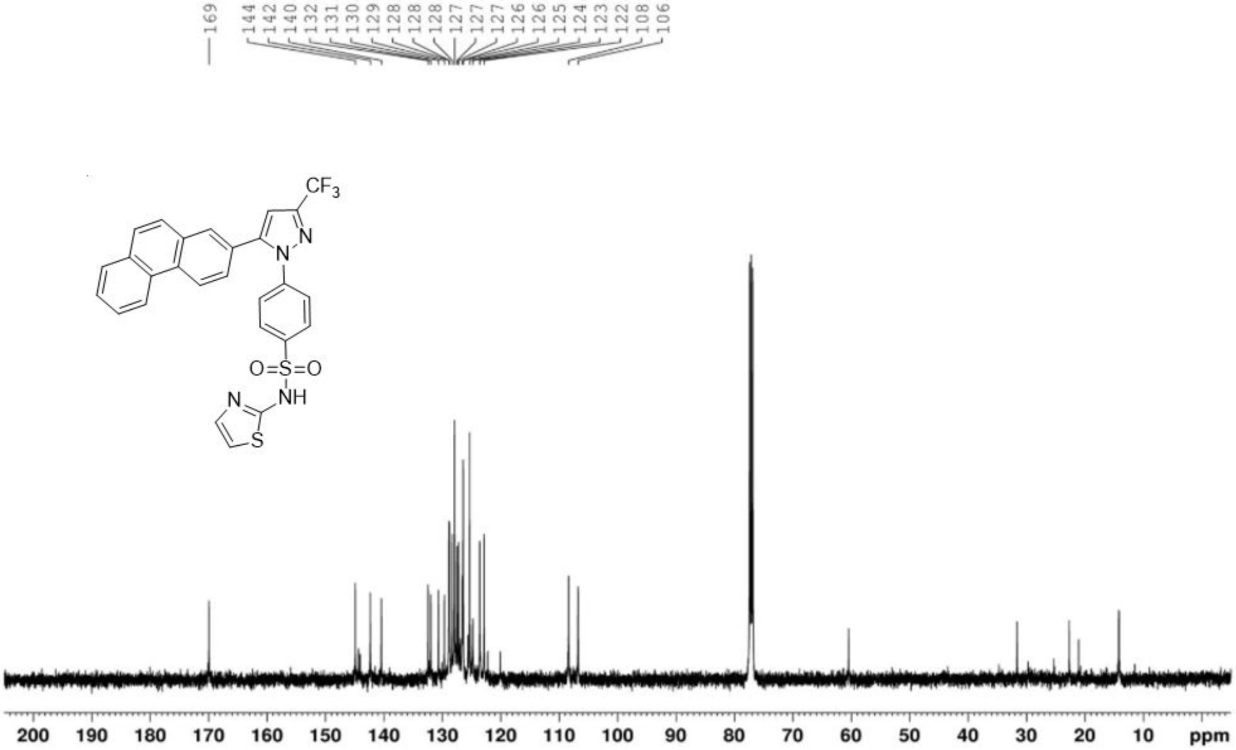

F: 1-(4-(methylsulfonyl)phenyl)-5-(phenanthren-2-yl)-3-(trifluoromethyl)-1H-pyrazole (6):

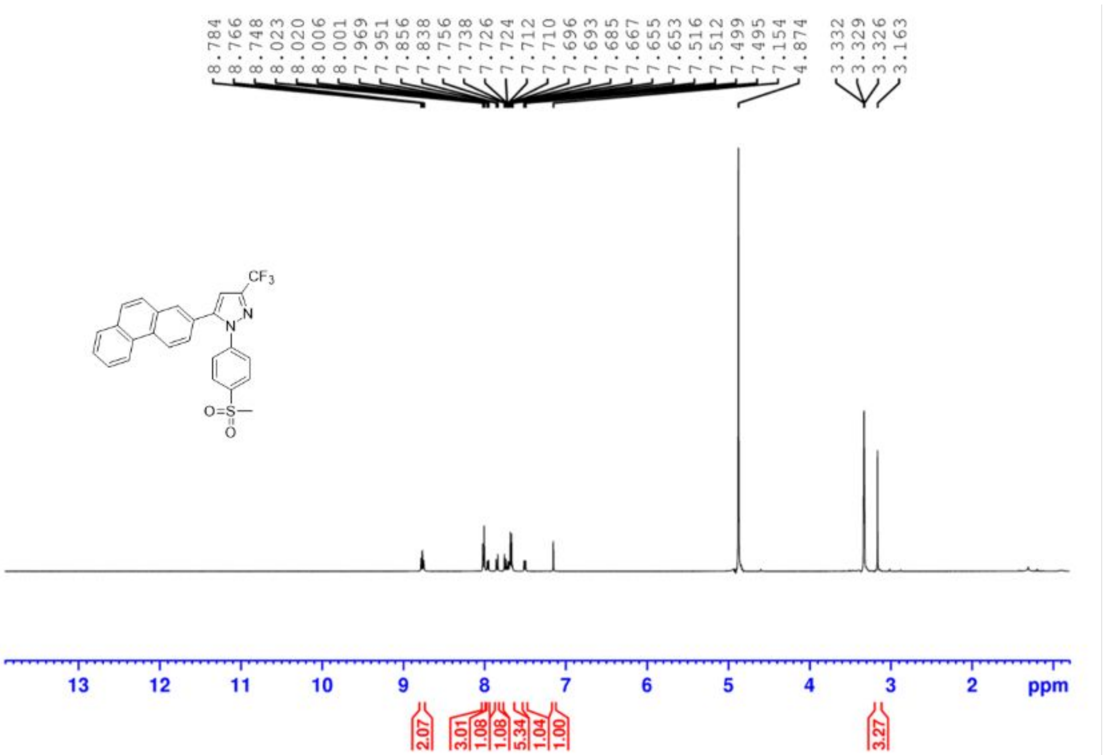

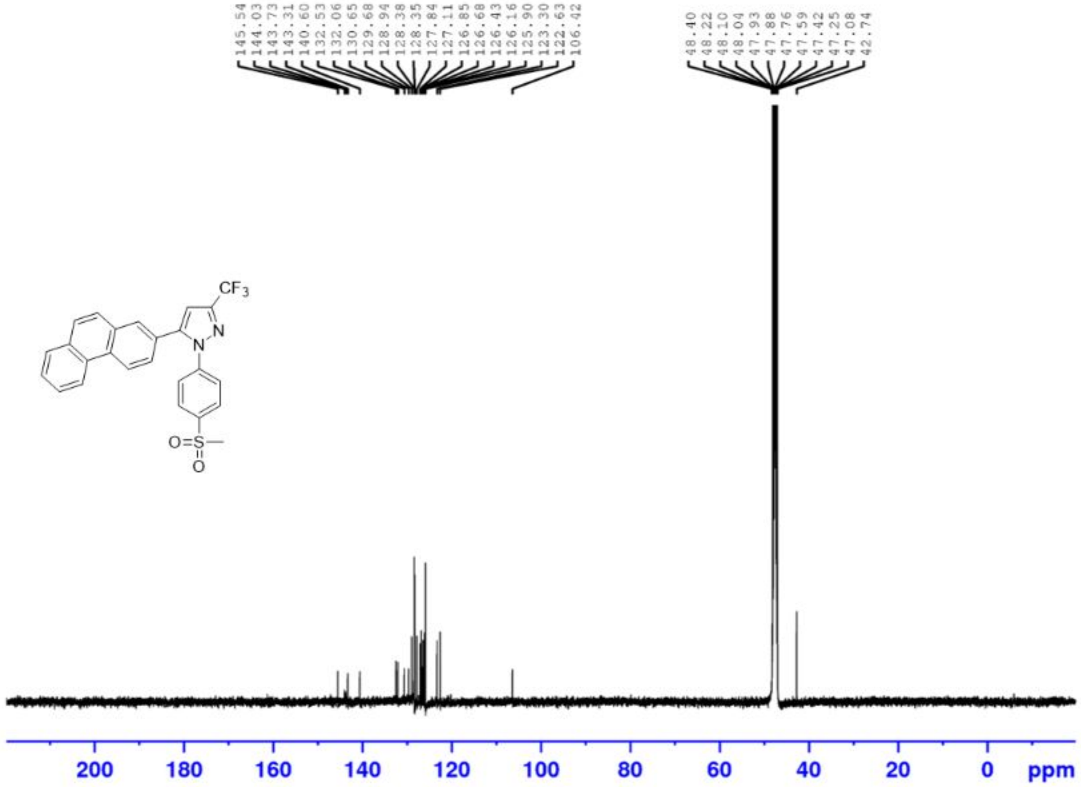

G: 4-(5-(phenanthren-2-yl)-3-(trifluoromethyl)-1H-pyrazol-1-yl)benzoic acid: (7)

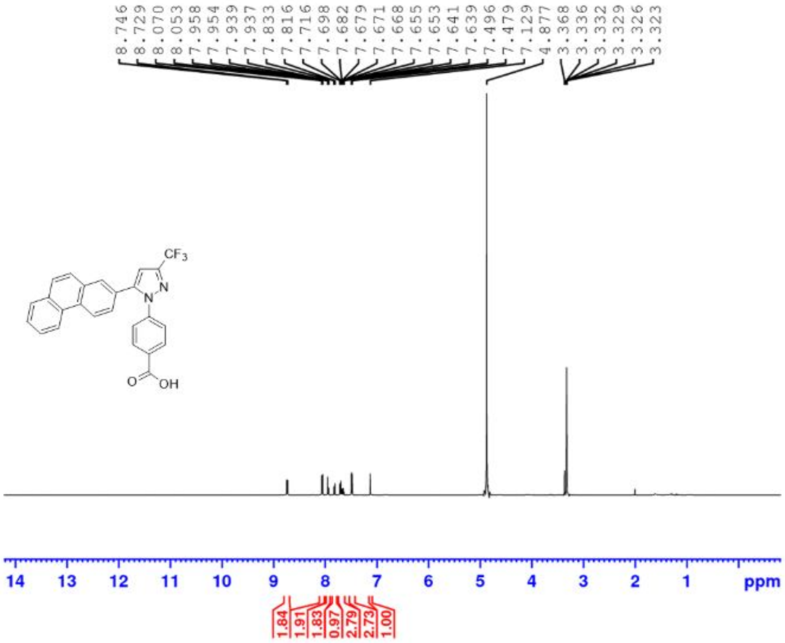

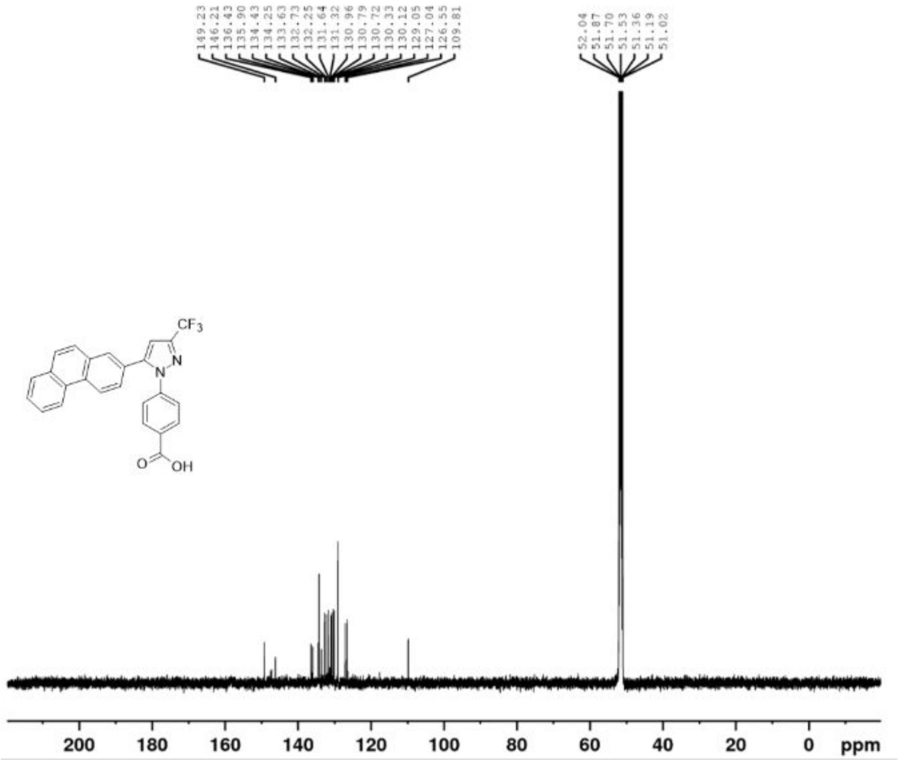

H: methyl 4-(5-(phenanthren-2-yl)-3-(trifluoromethyl)-1H-pyrazol-1-yl)benzoate compound: (8)

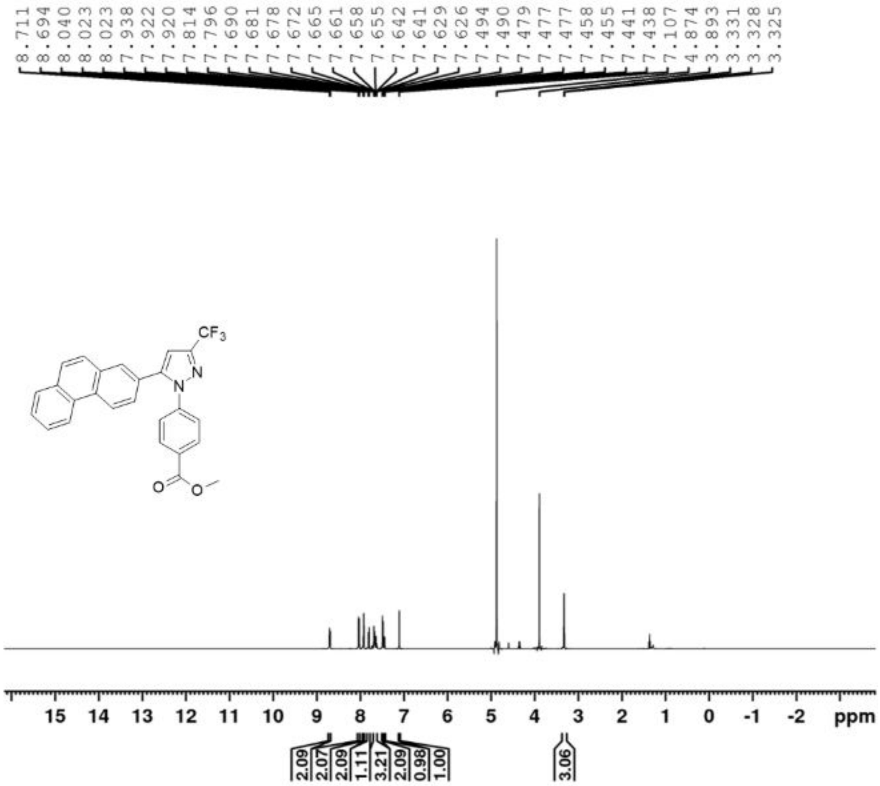

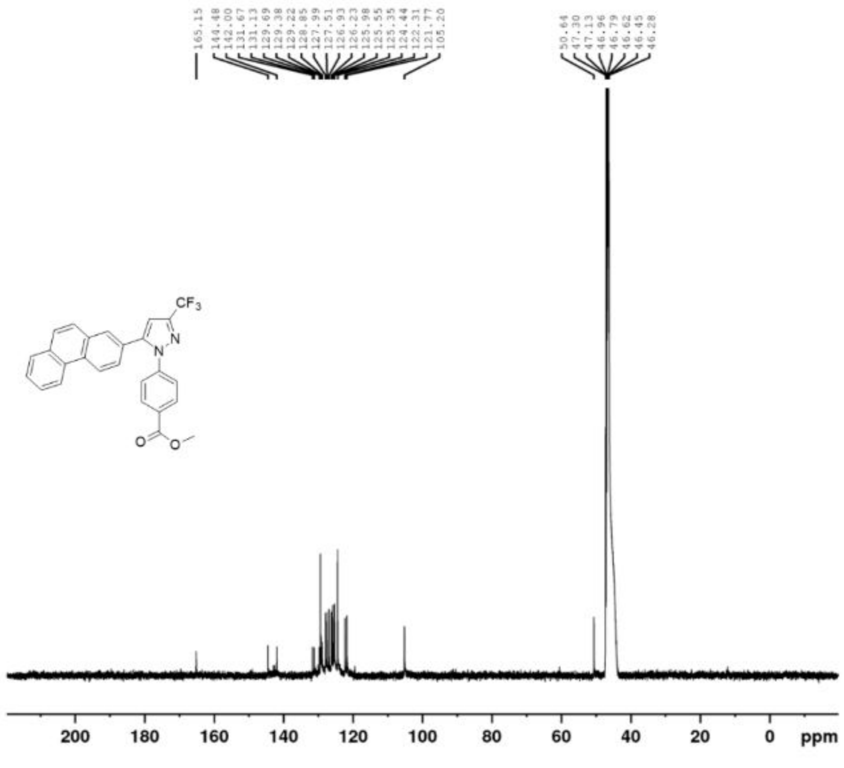

I: 4-(5-(phenanthren-2-yl)-3-(trifluoromethyl)-1H-pyrazol-1-yl)benzamide (9):

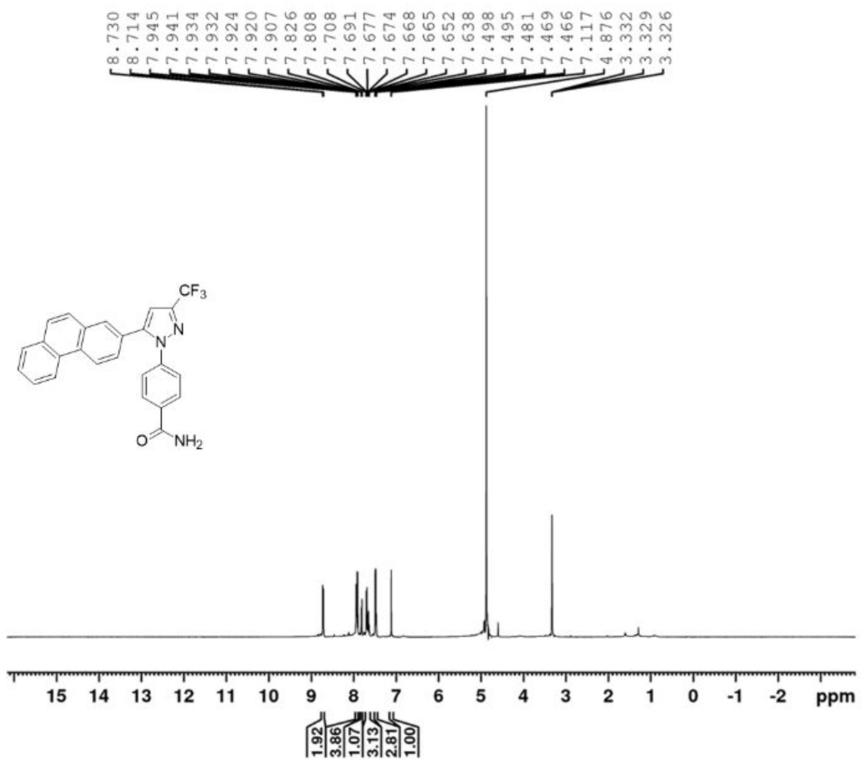

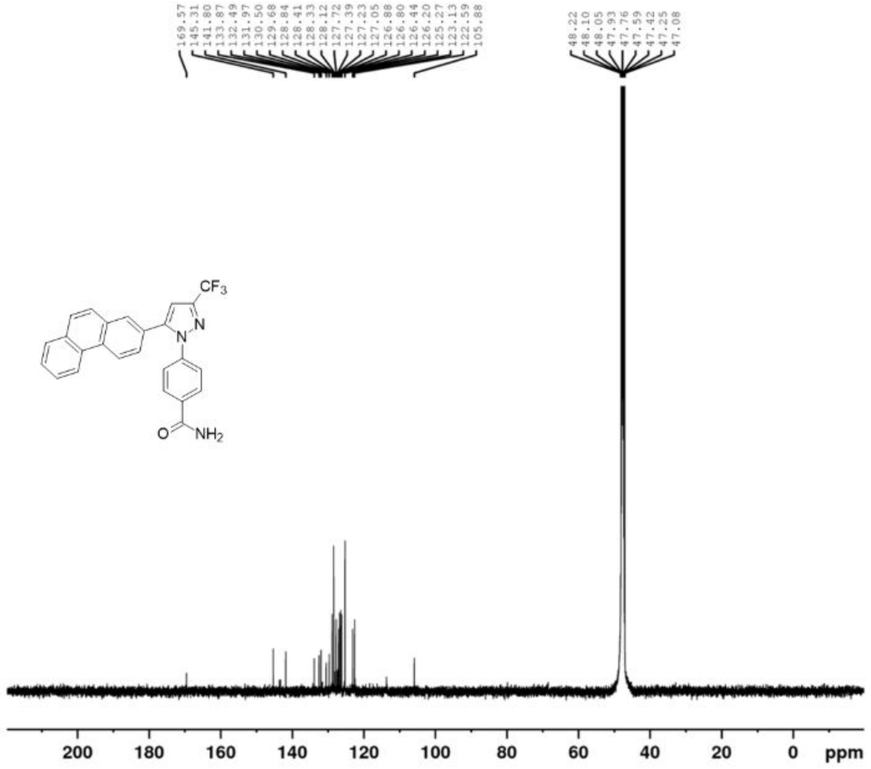

J: 2-(5-(phenanthren-2-yl)-3-(trifluoromethyl)-1H-pyrazol-1-yl)benzoic acid (10)

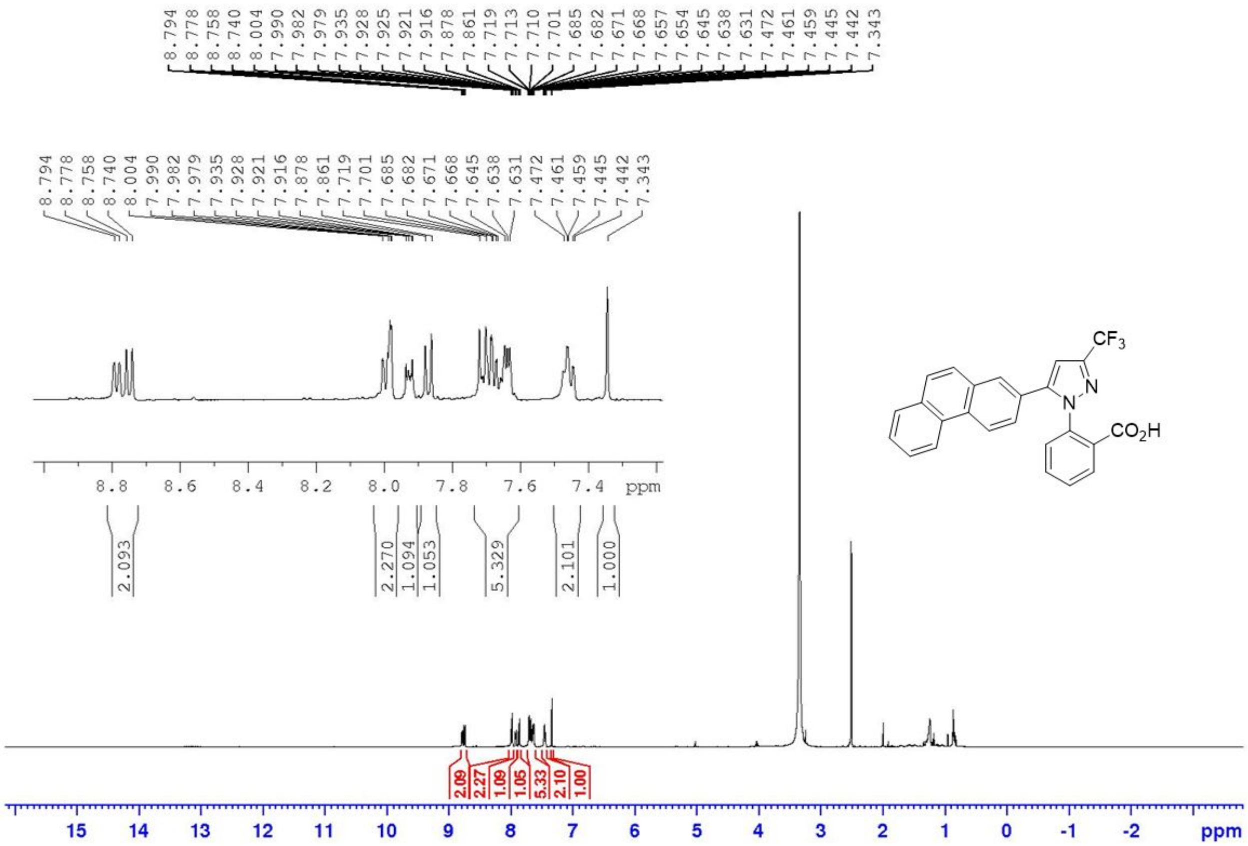

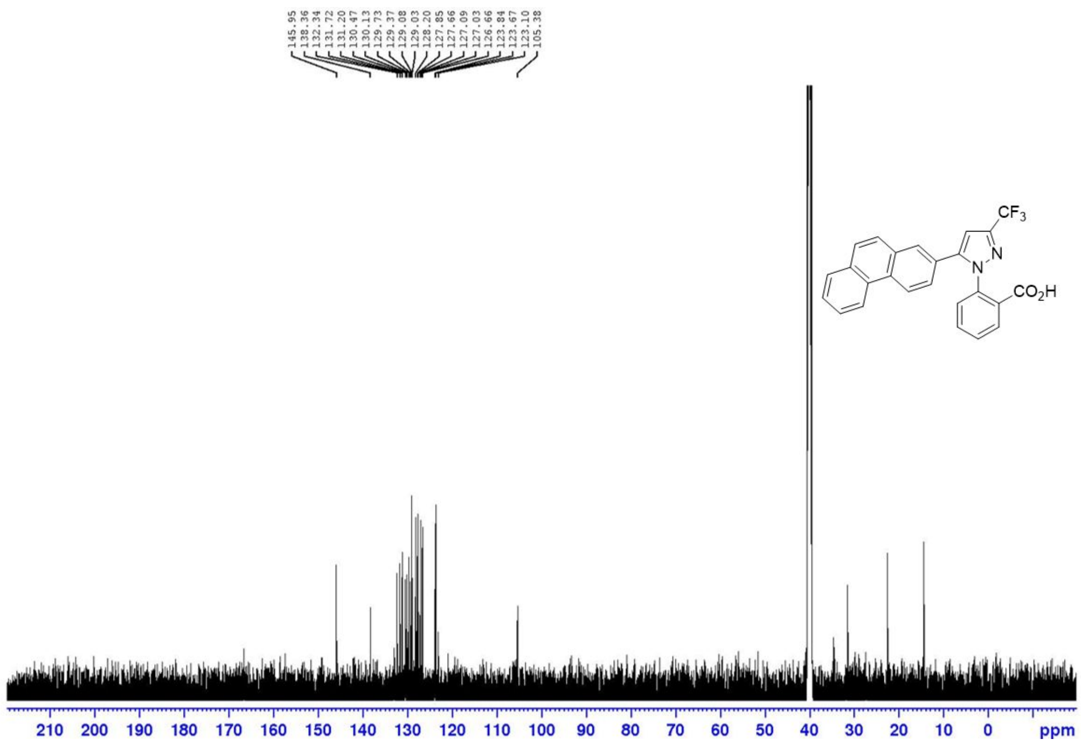

K: 3-(5-(phenanthren-2-yl)-3-(trifluoromethyl)-1H-pyrazol-1-yl)benzoic acid (11)

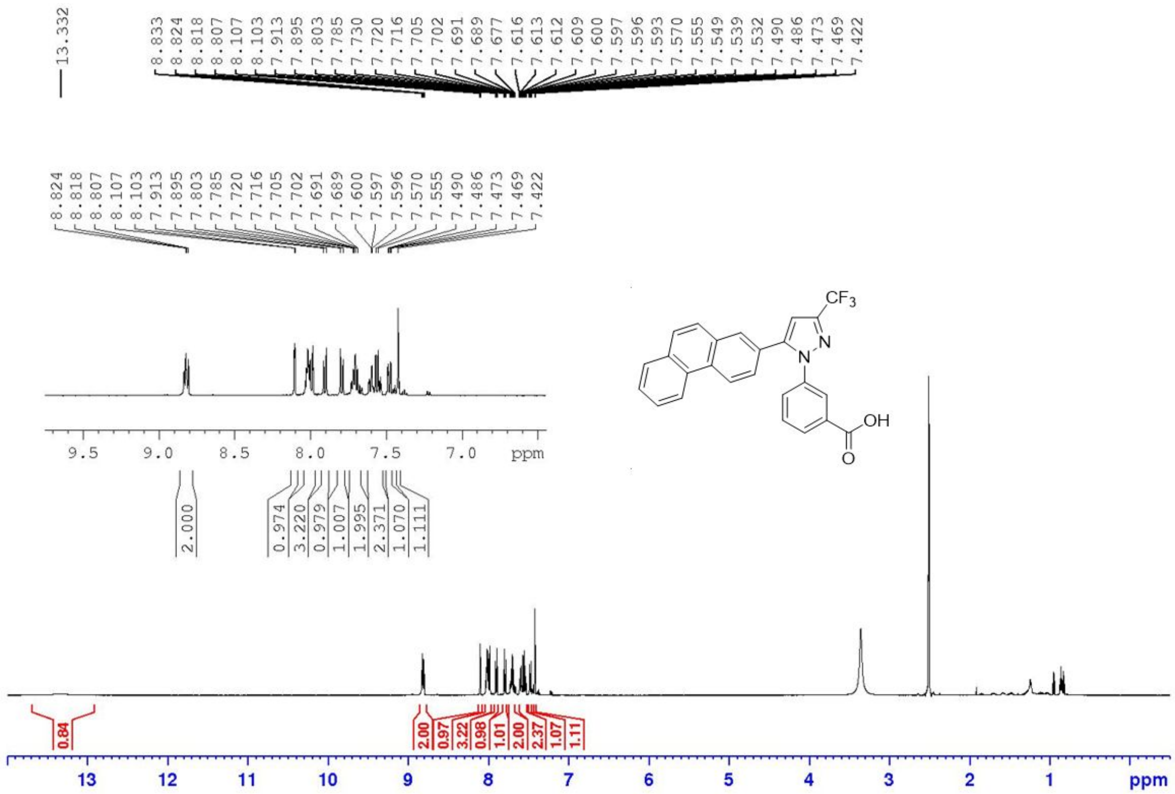

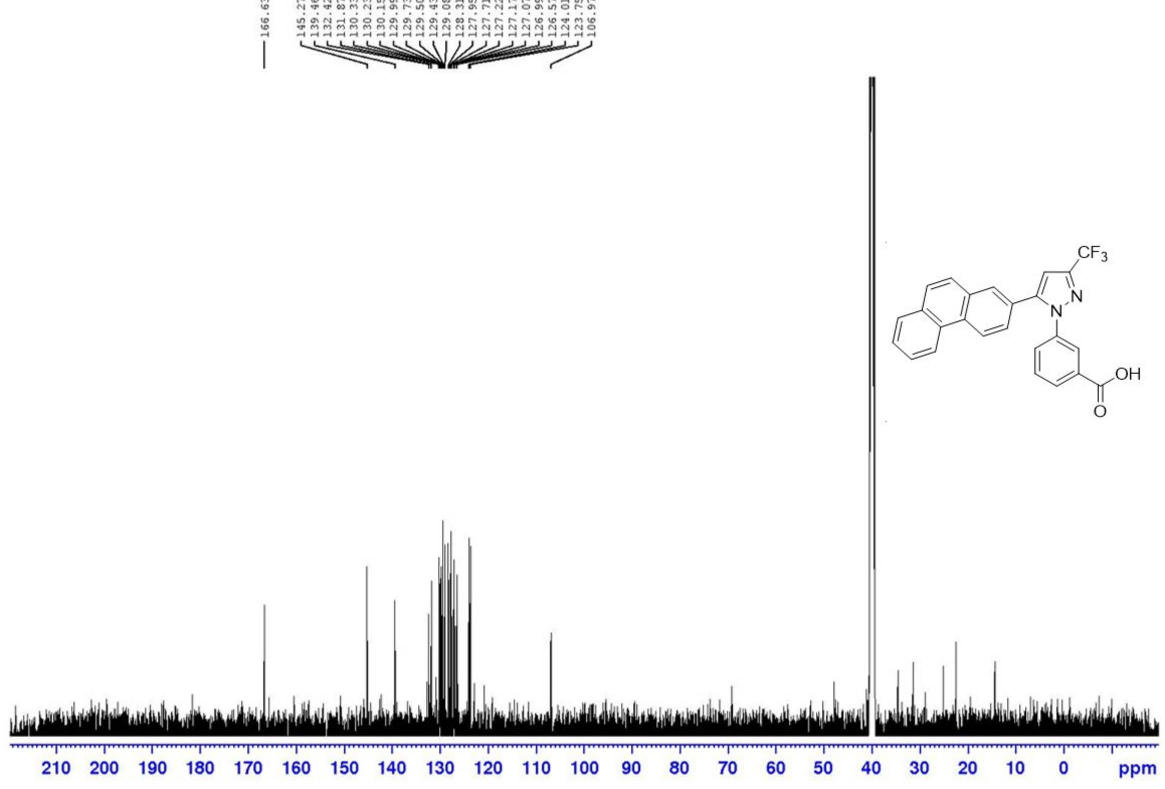

L: 5-(4-(5-(phenanthren-2-yl)-3-(trifluoromethyl)-1H-pyrazol-1-yl)phenyl)-1H-tetrazole: (12)

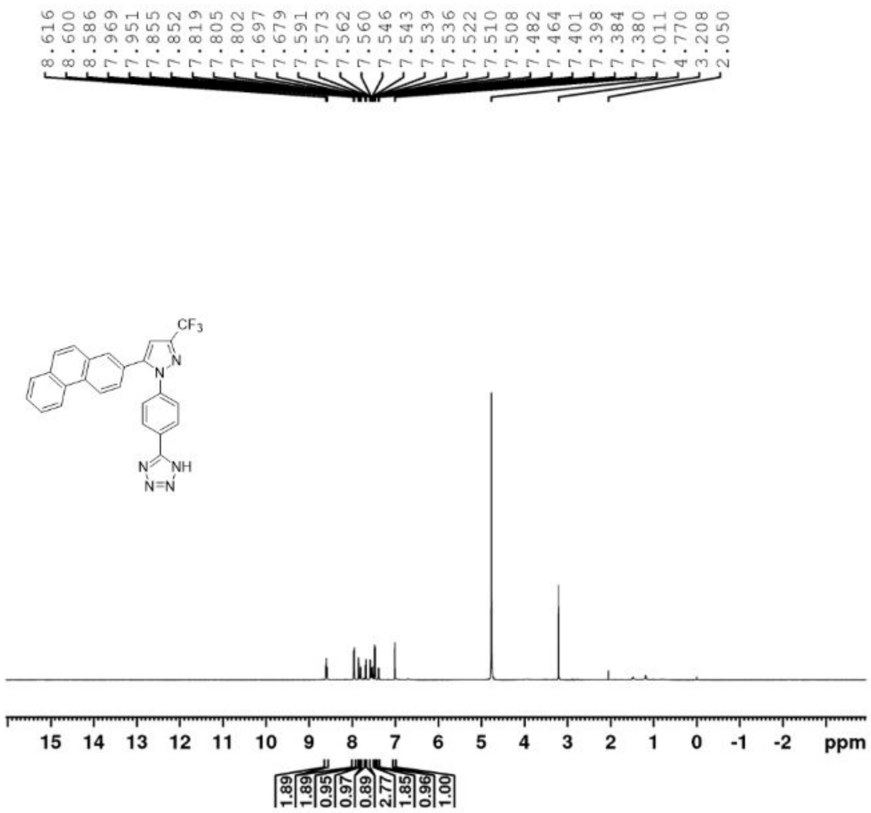
8.616
8.600
8.586
7.969
7.951
7.855
7.852
7.819
7.805
7.802
7.697
7.679
7.591
7.573
7.562
7.560
7.546
7.543
7.539
7.536
7.522
7.510
7.508
7.482
7.464
7.401
7.398
7.384
7.380
7.011
4.770
3.208
2.050
CF3
N
N
N
NH
N=N
15 14 13 12 11 10 9 8 7 6 5 4 3 2 1 0 -1 -2 ppm
1.89
1.89
0.95
0.97
0.89
2.77
1.85
0.96
1.00

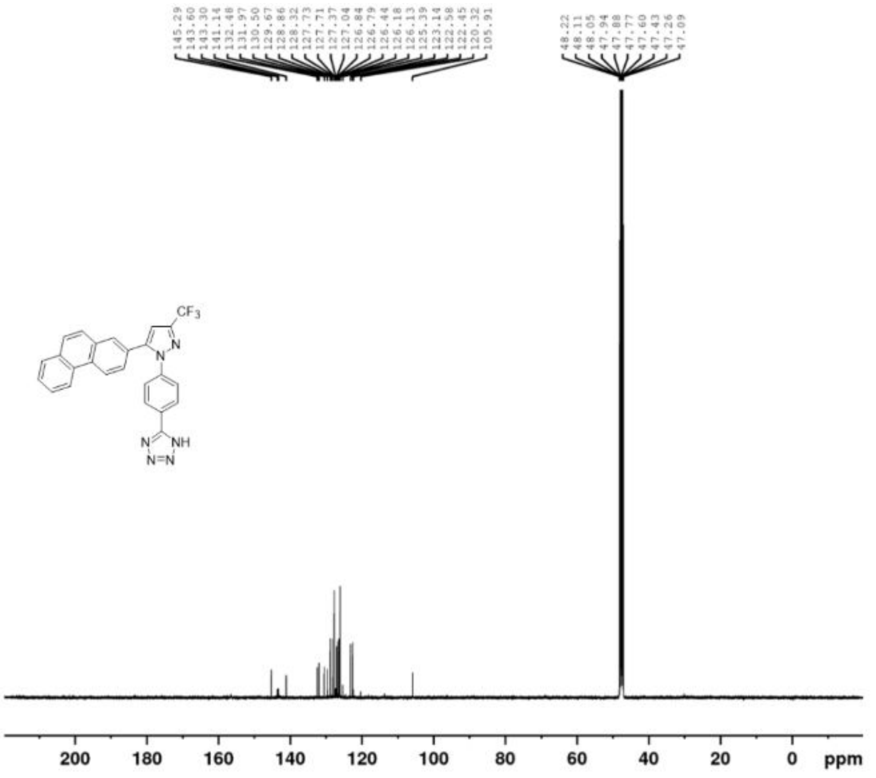
145.29
143.60
143.30
141.14
132.48
131.97
130.50
129.67
128.86
128.32
127.73
127.71
127.37
127.04
126.84
126.79
126.44
126.18
126.13
125.39
123.14
122.58
122.45
120.32
105.91
48.22
48.11
48.05
47.94
47.88
47.77
47.60
47.43
47.26
47.09
CF3
N
N
N
NH
N=N
200 180 160 140 120 100 80 60 40 20 0 ppm

M: sodium 3-oxo-5-(5-(phenanthren-2-yl)-3-(trifluoromethyl)-1H-pyrazol-1-yl)-3H-benzo[d]isothiazol-2-ide 1,1-dioxide (13, *sodium salt*)

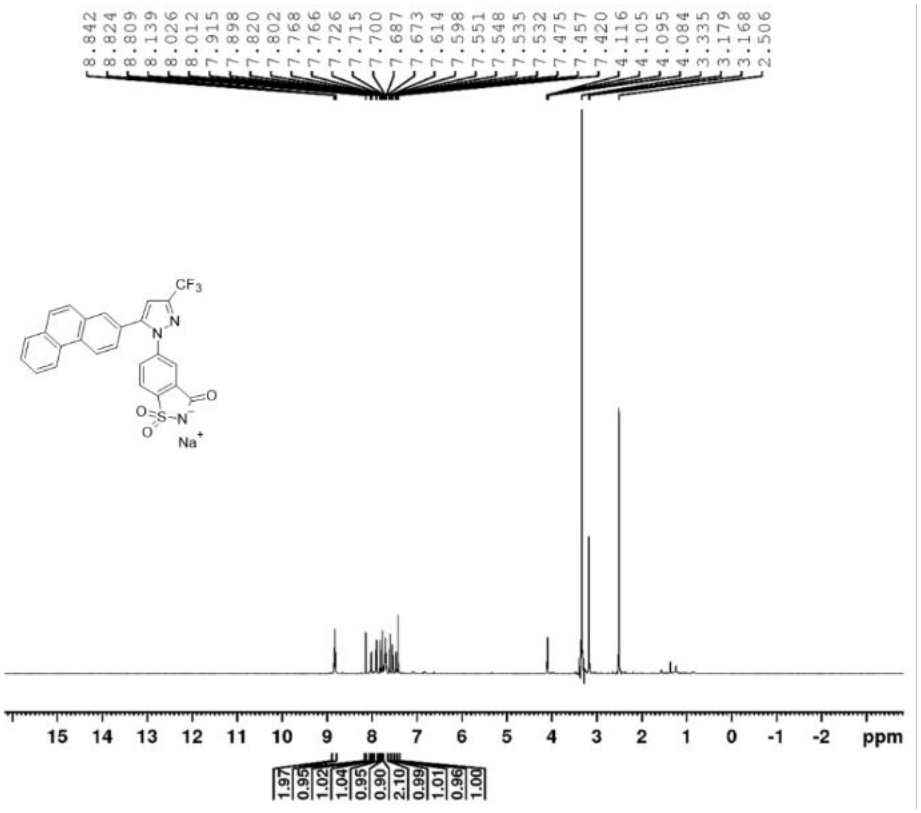

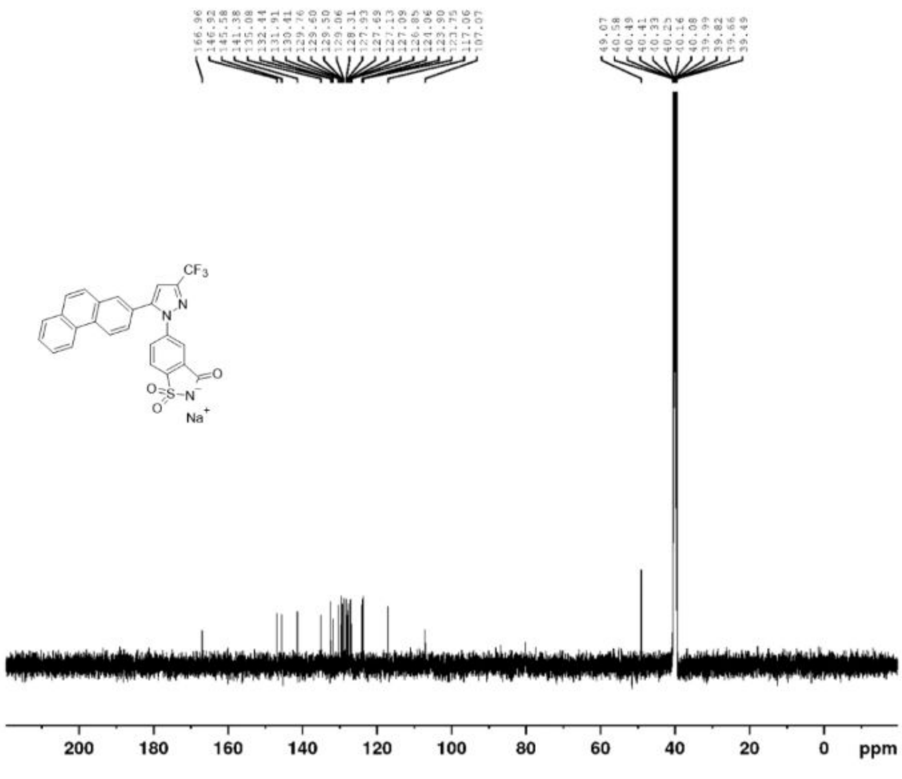

N: 5-(5-(phenanthren-2-yl)-3-(trifluoromethyl)-1H-pyrazol-1-yl)benzo[d]isothiazol-3(2H)-one 1,1-dioxide (13, *free base)*

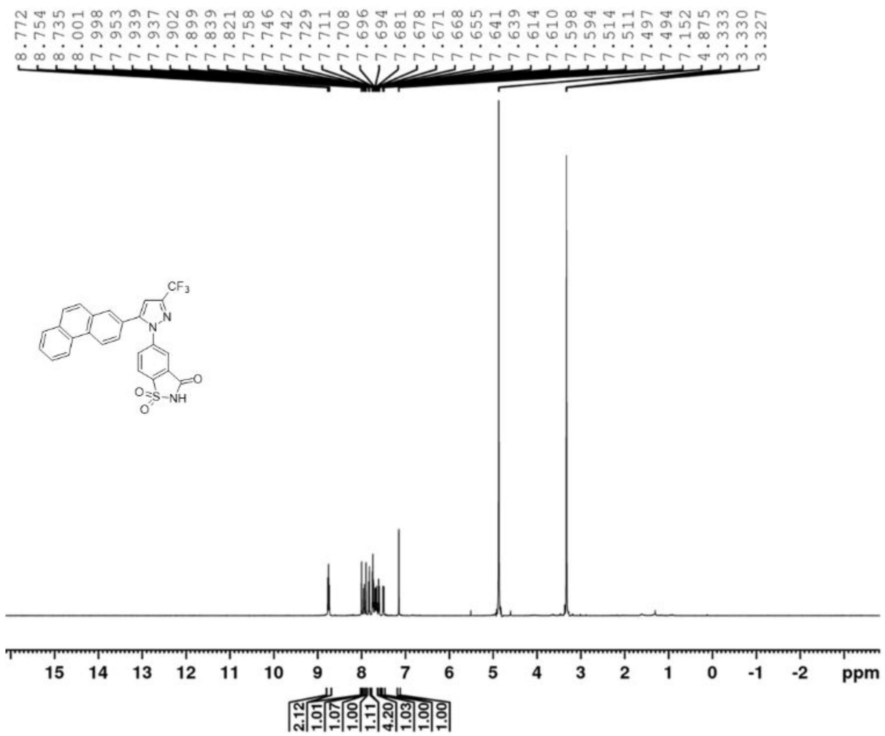

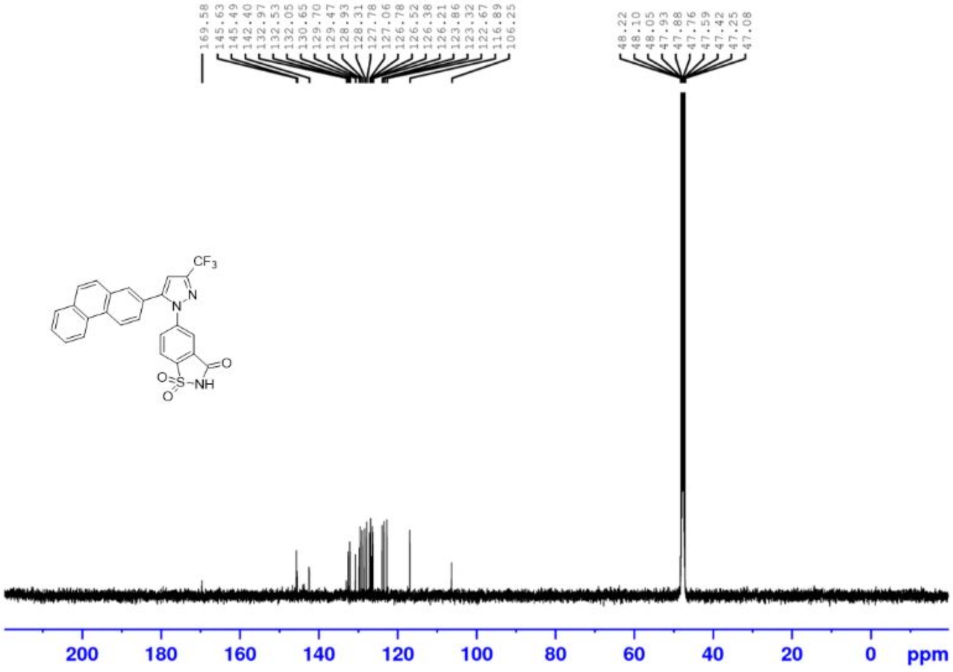

O: 4-(5-(4-chlorophenyl)-3-(trifluoromethyl)-1H-pyrazol-1-yl)benzoic acid (14)

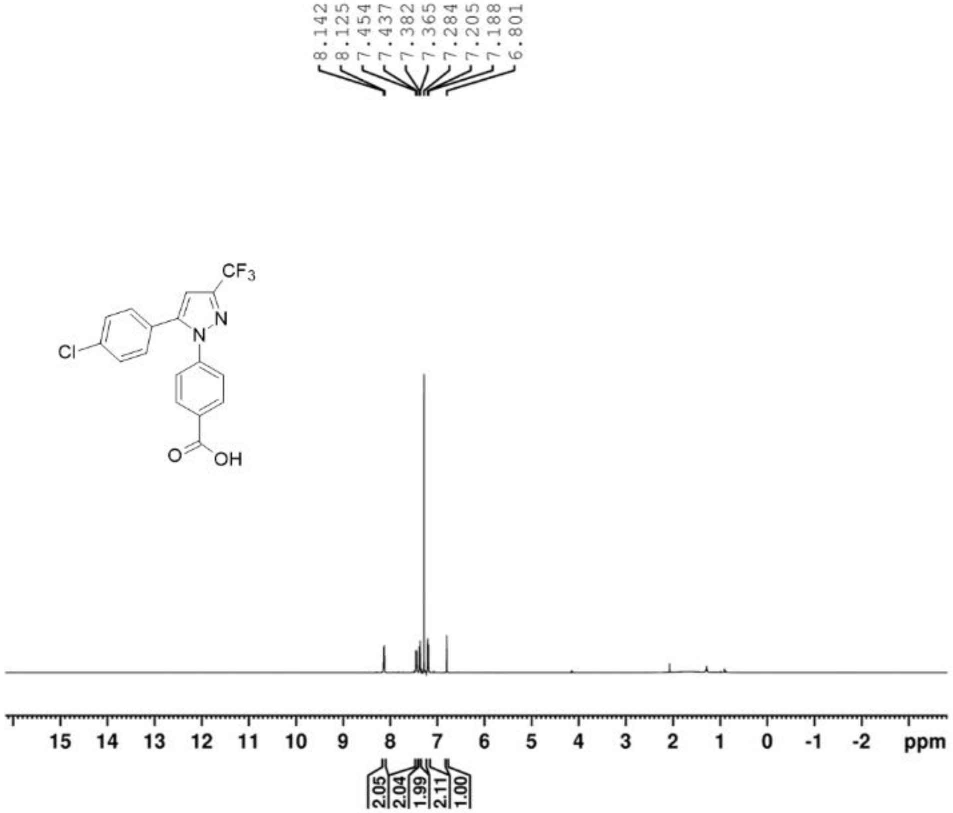

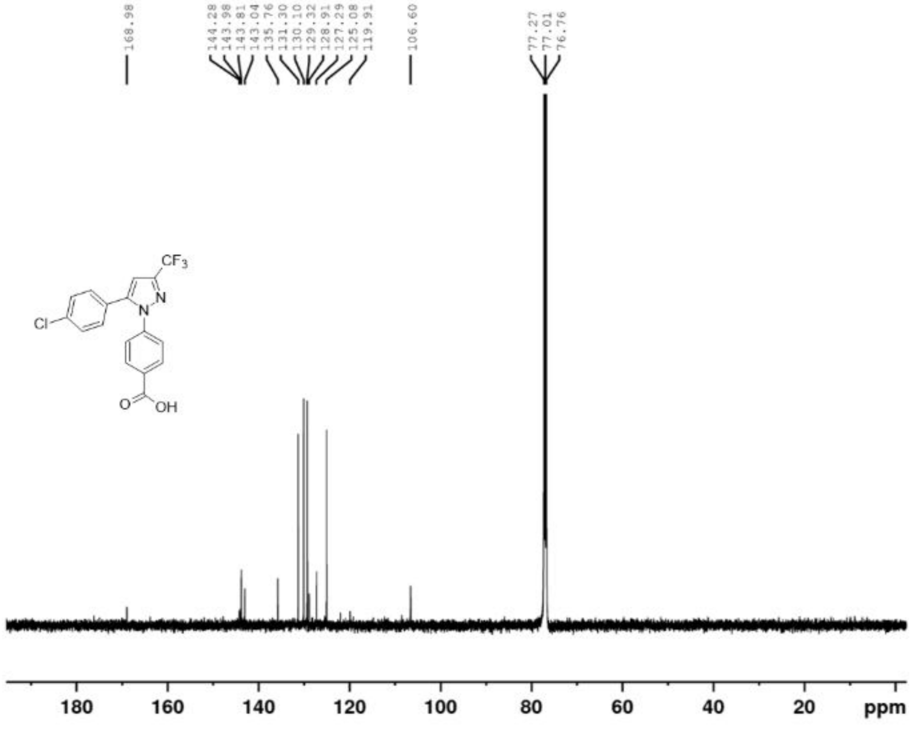

P: 4-(5-phenyl-3-(trifluoromethyl)-1H-pyrazol-1-yl)benzoic acid (15)

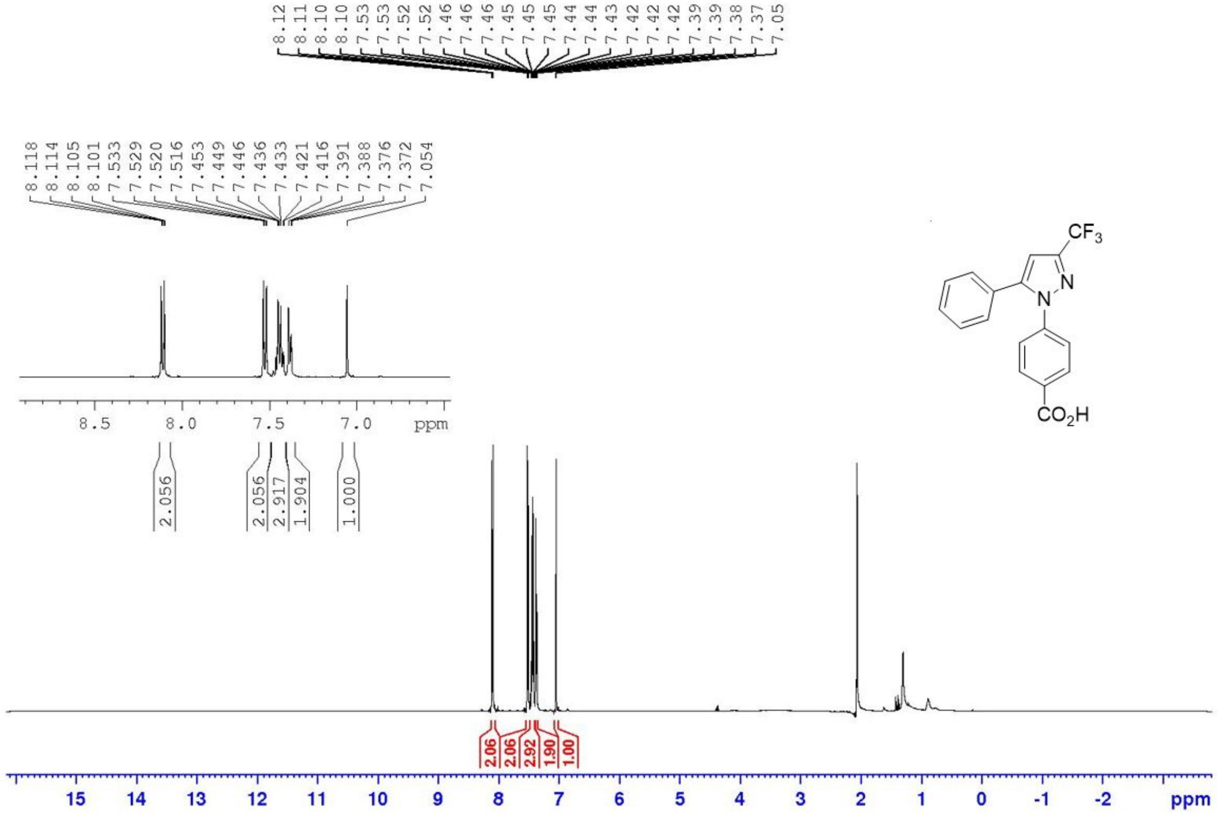

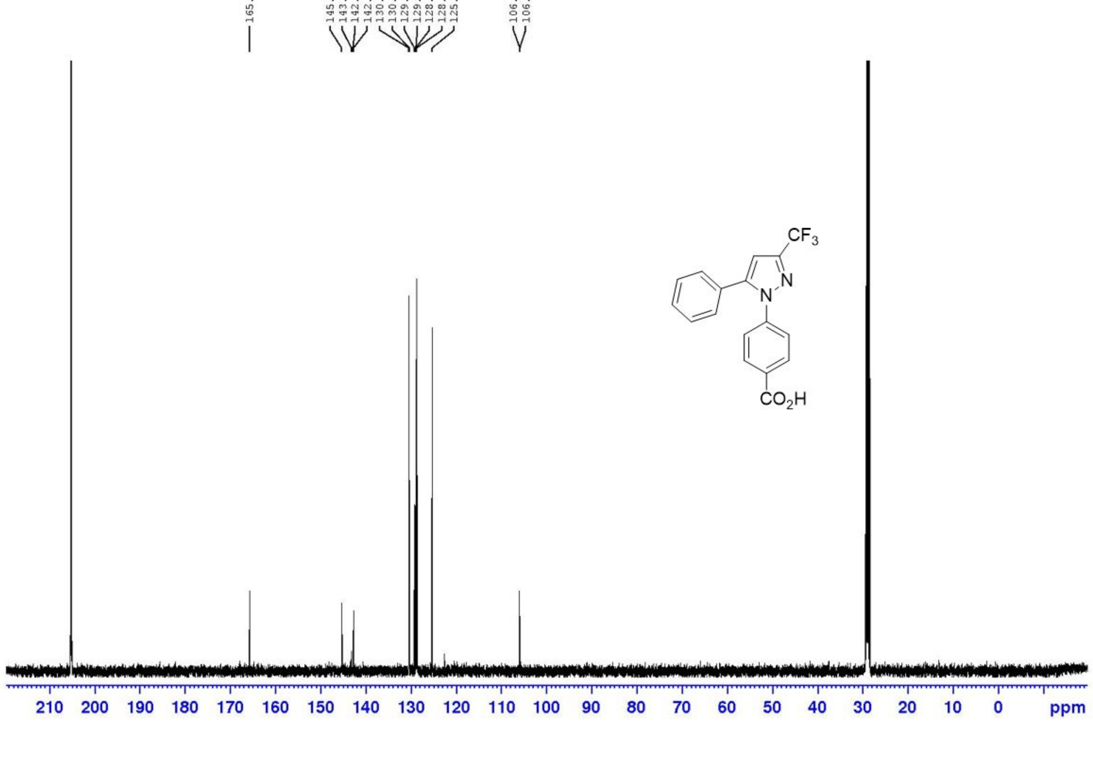

Q: 4-(5-(naphthalen-2-yl)-3-(trifluoromethyl)-1H-pyrazol-1-yl)benzoic acid (16)

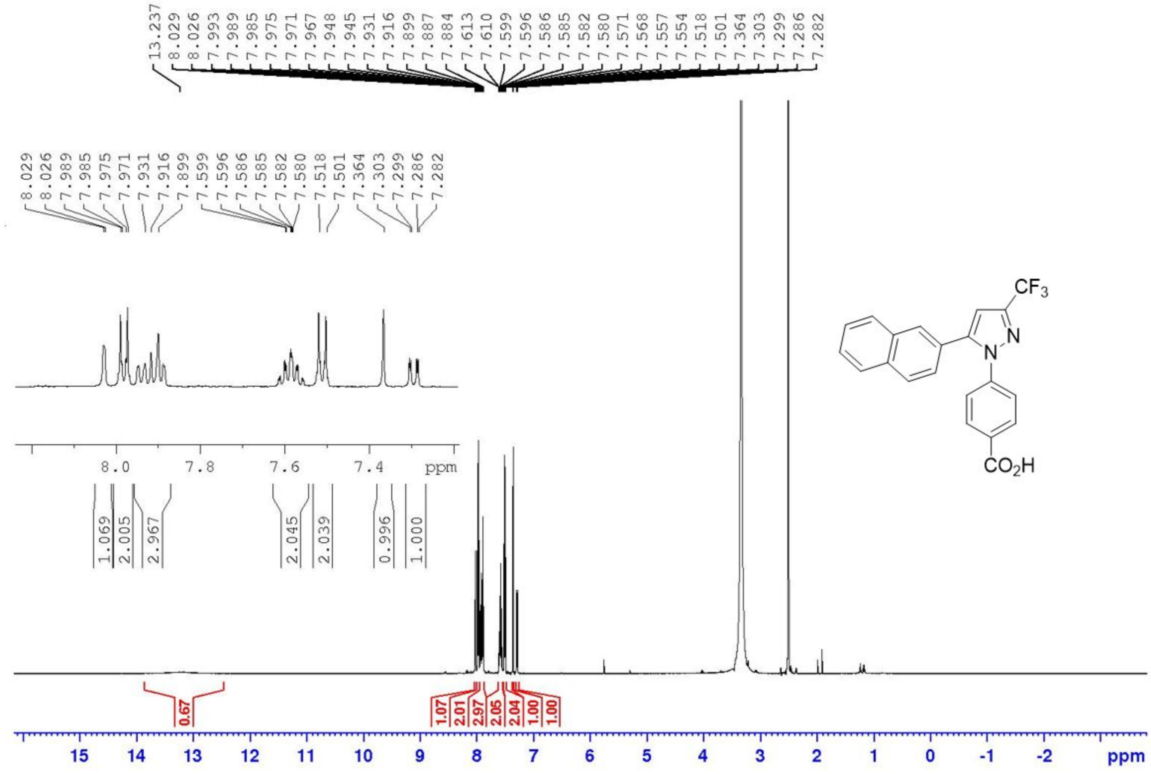

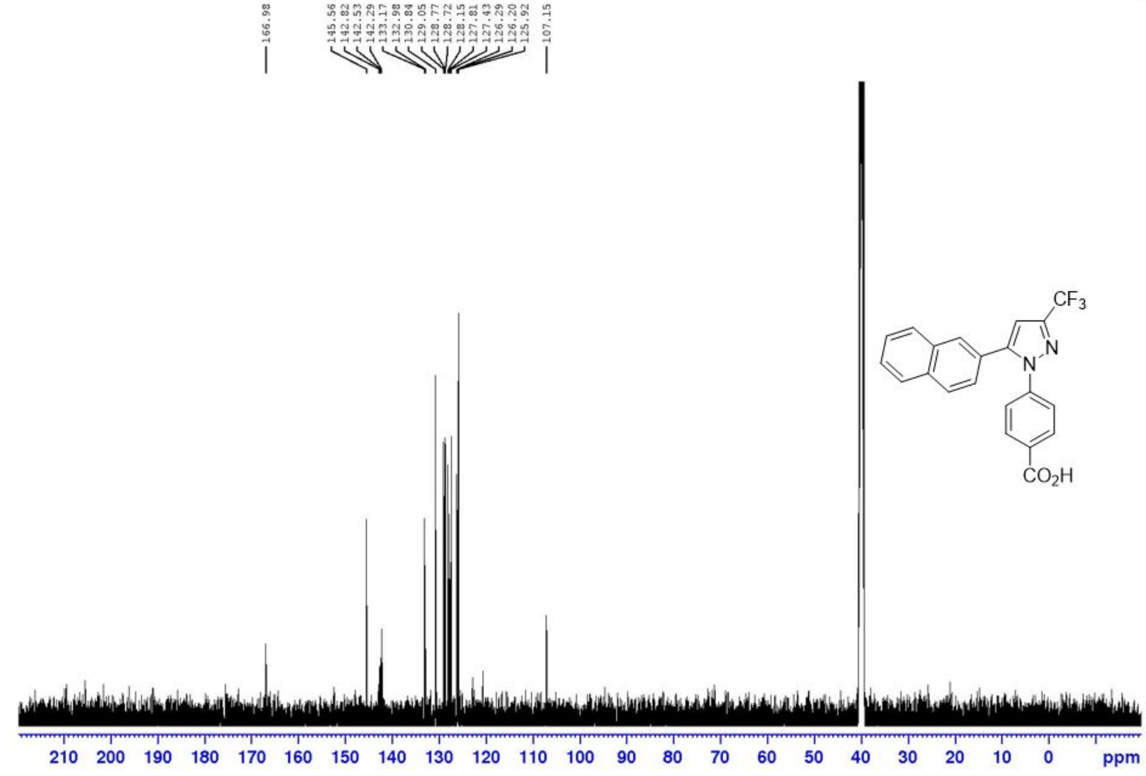

R: 4-(5-([1,1'-biphenyl]-4-yl)-3-(trifluoromethyl)-1H-pyrazol-1-yl)benzoic acid (17)

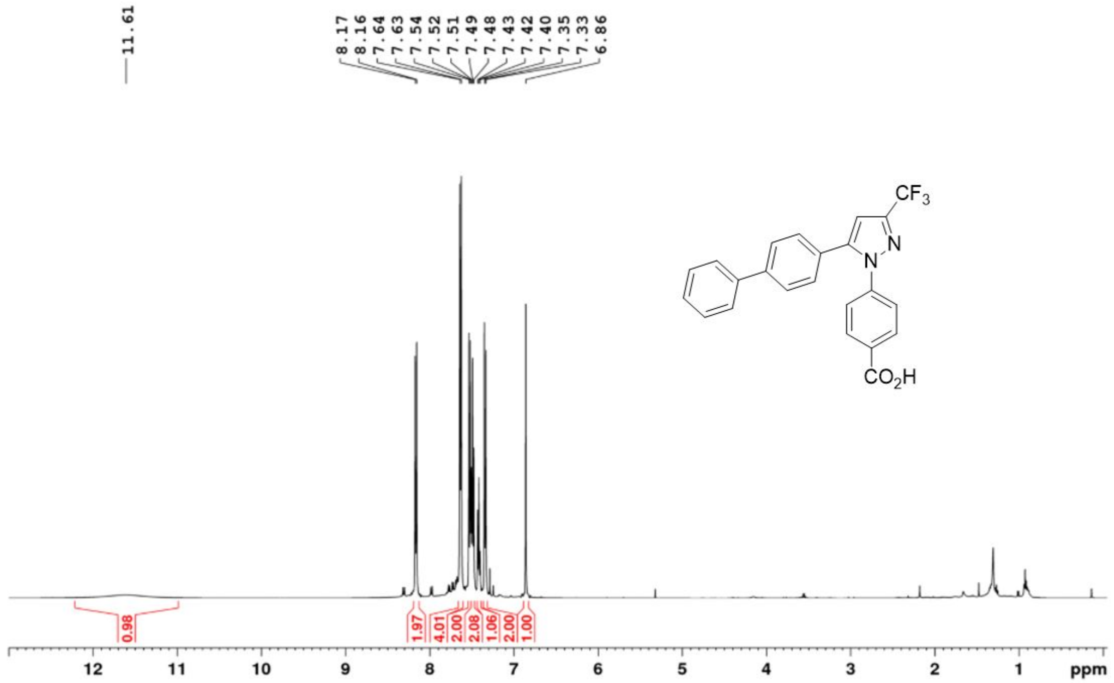

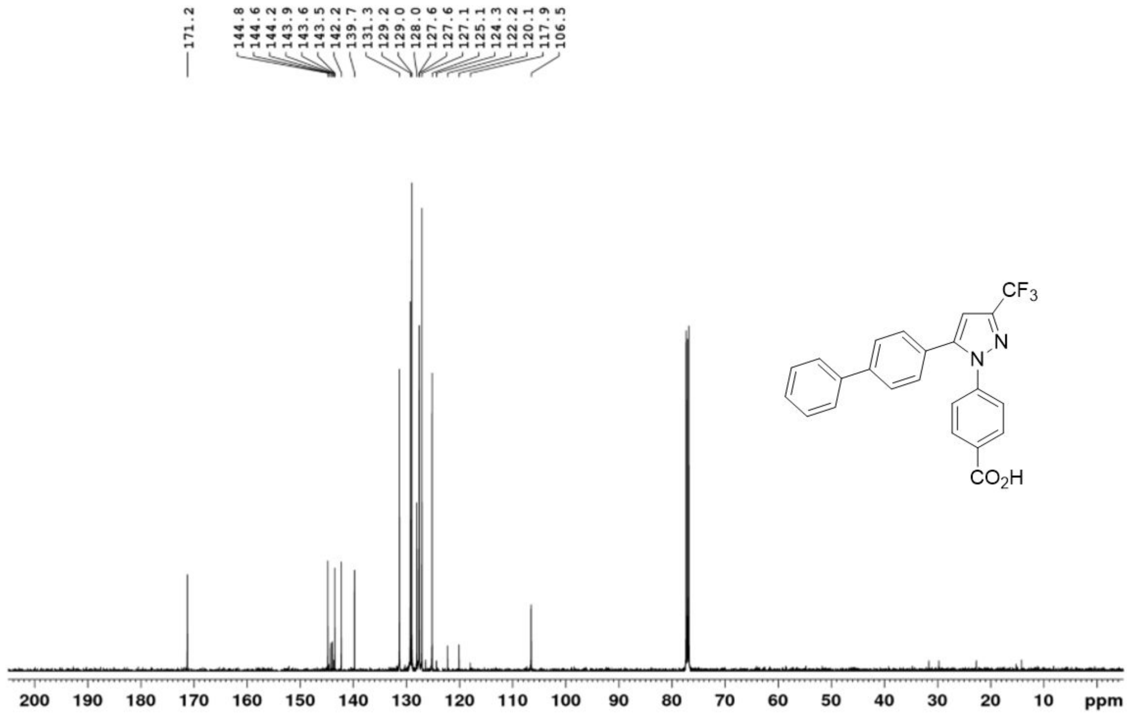

S: 4-(5-(4-bromophenyl)-3-(trifluoromethyl)-1H-pyrazol-1-yl)benzoic acid (18)

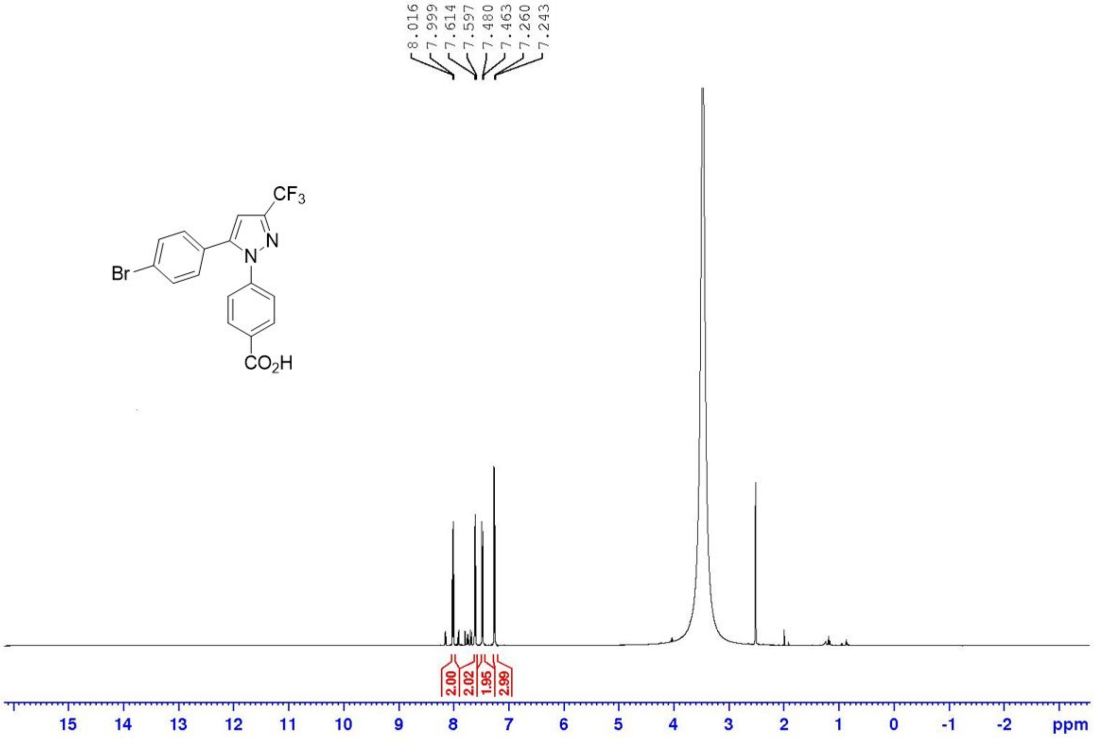

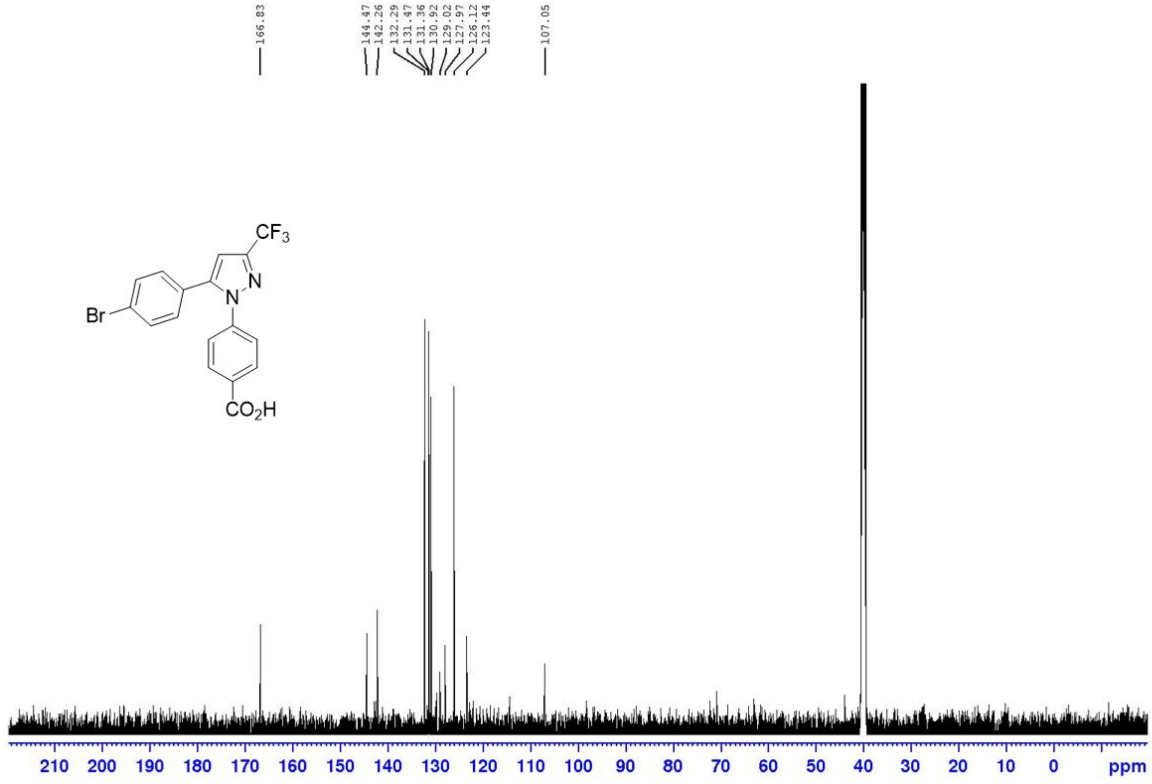

T: 4-(5-([1,1'-biphenyl]-3-yl)-3-(trifluoromethyl)-1H-pyrazol-1-yl)benzoic acid (19)

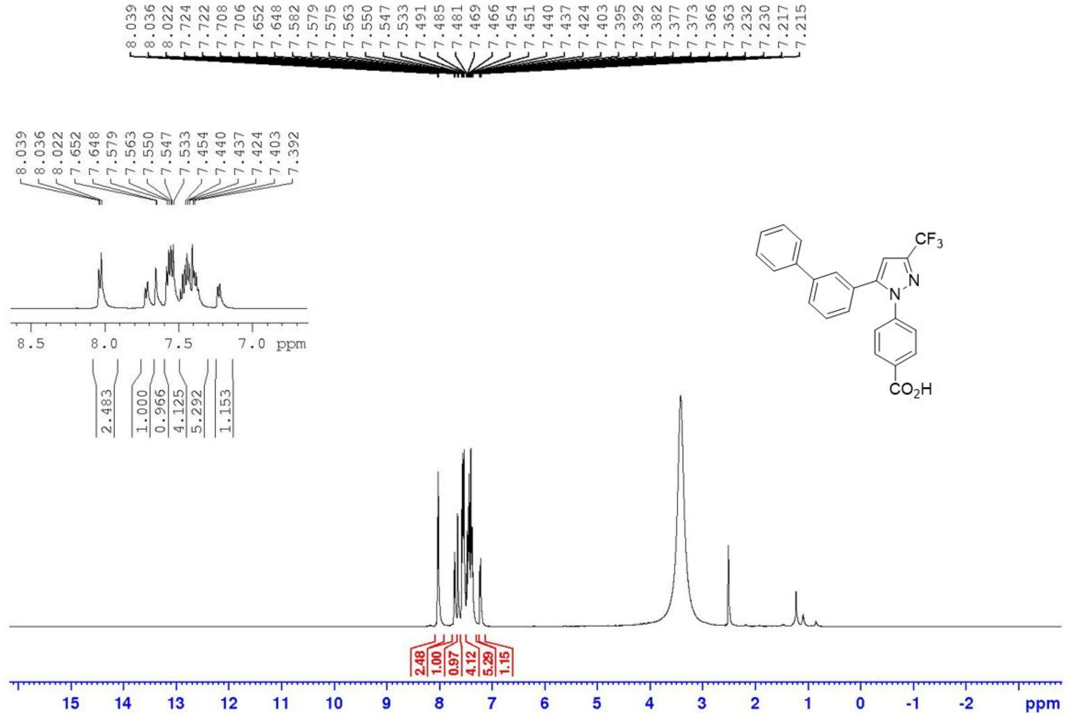

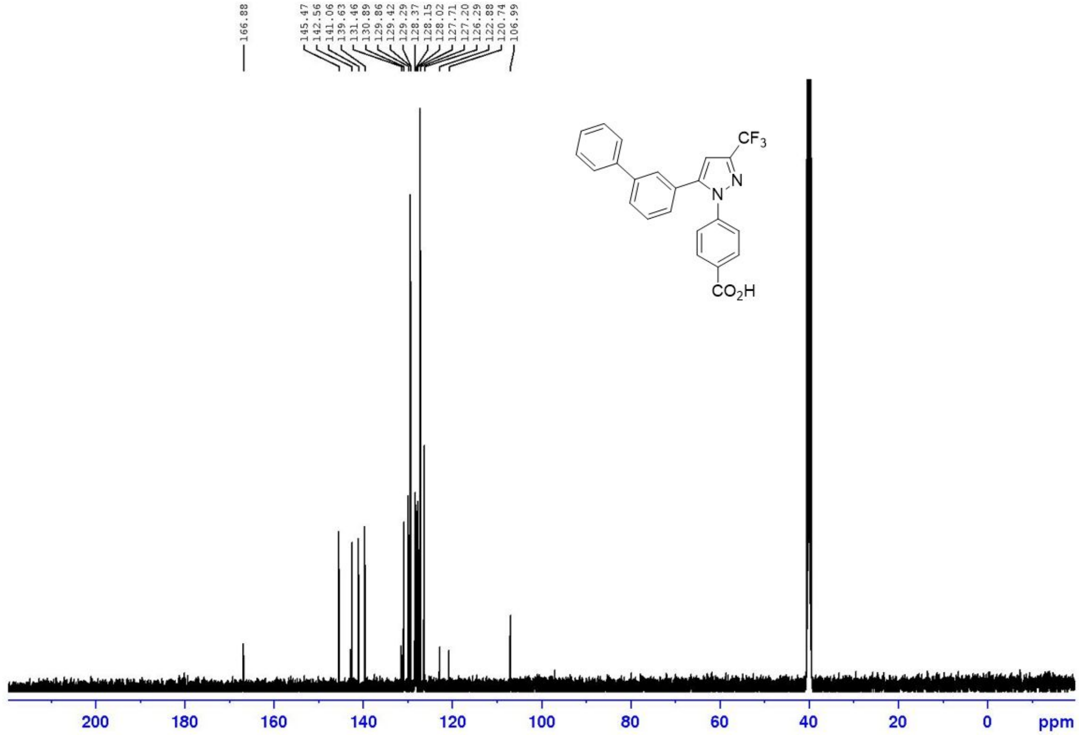

U: 4-(5-(2'-methoxy-[1,1'-biphenyl]-4-yl)-3-(trifluoromethyl)-1H-pyrazol-1-yl)benzoic acid (20)

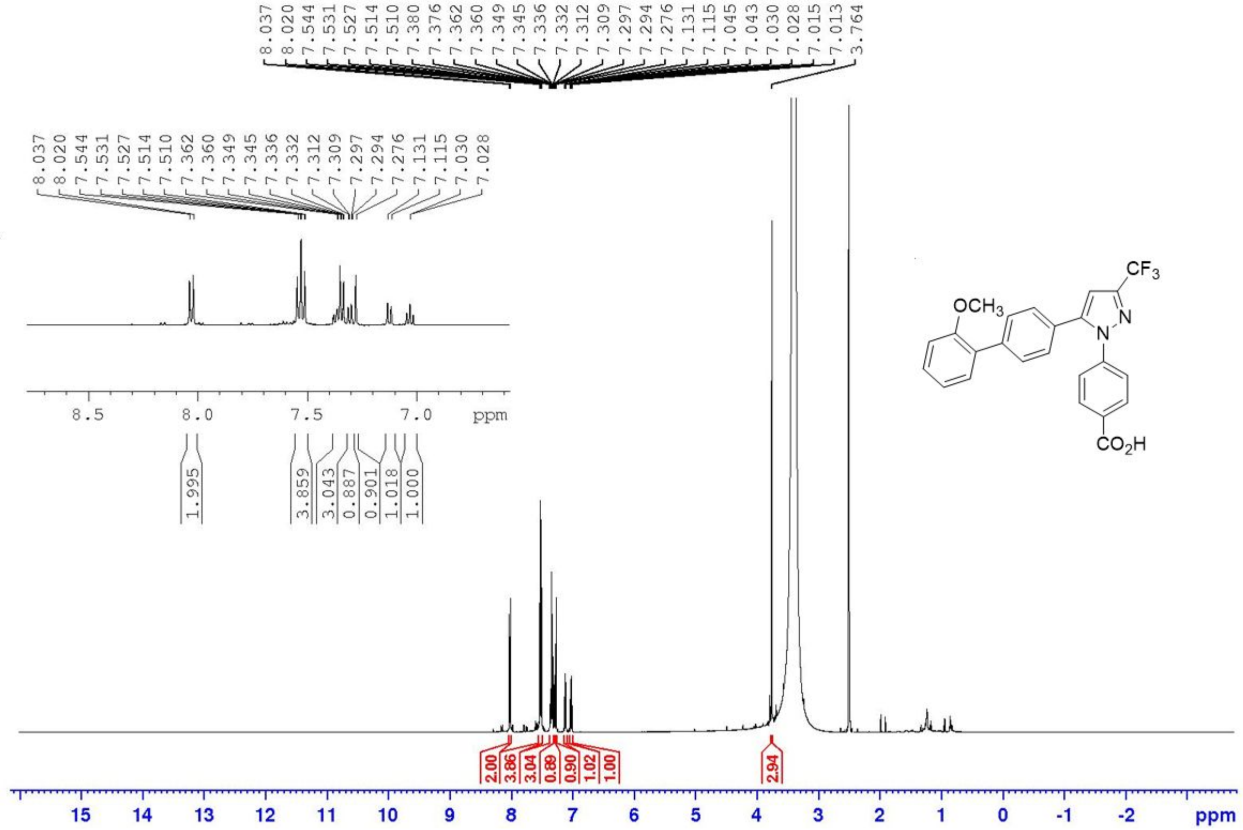

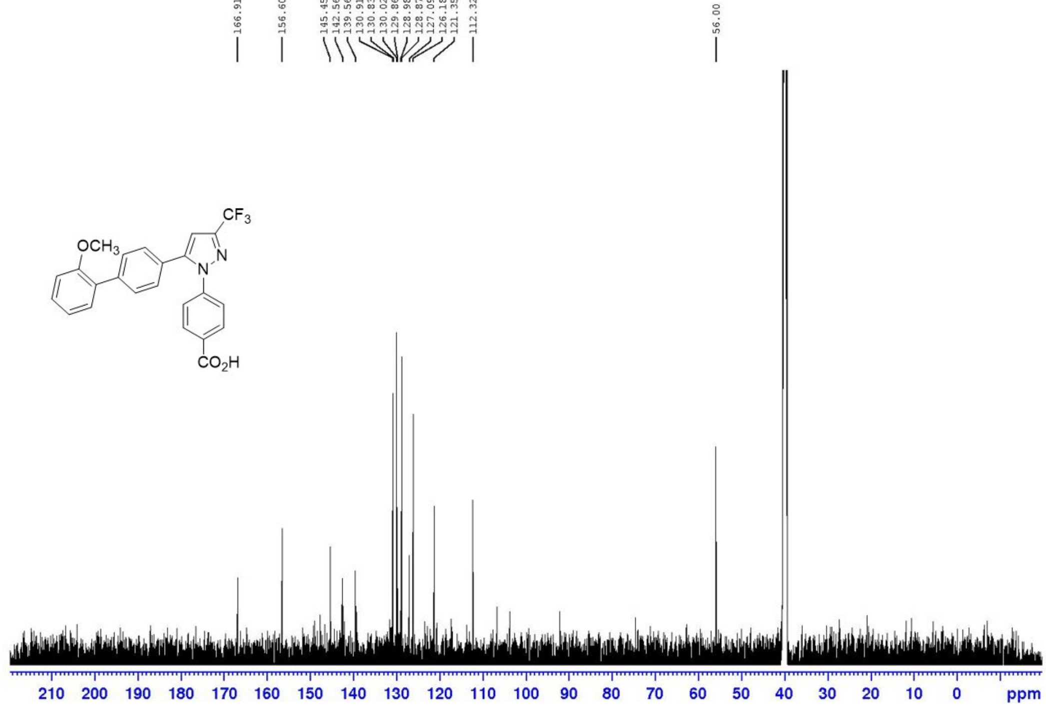

V: ethyl 4-(5-((4-isopropylbenzyl)oxy)-3-(trifluoromethyl)-1H-pyrazol-1-yl)benzoate (21a)

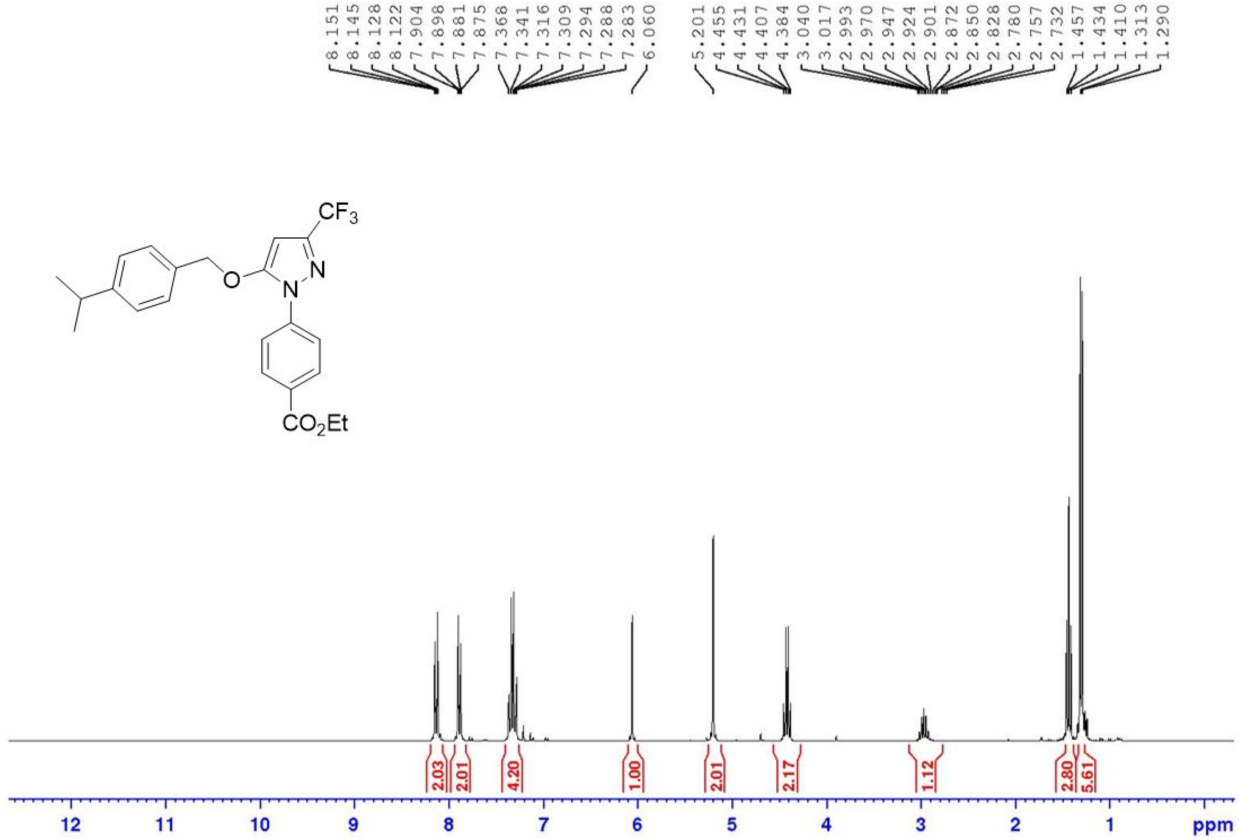

W: 4-(5-((4-isopropylbenzyl)oxy)-3-(trifluoromethyl)-1H-pyrazol-1-yl)benzoic acid (21)

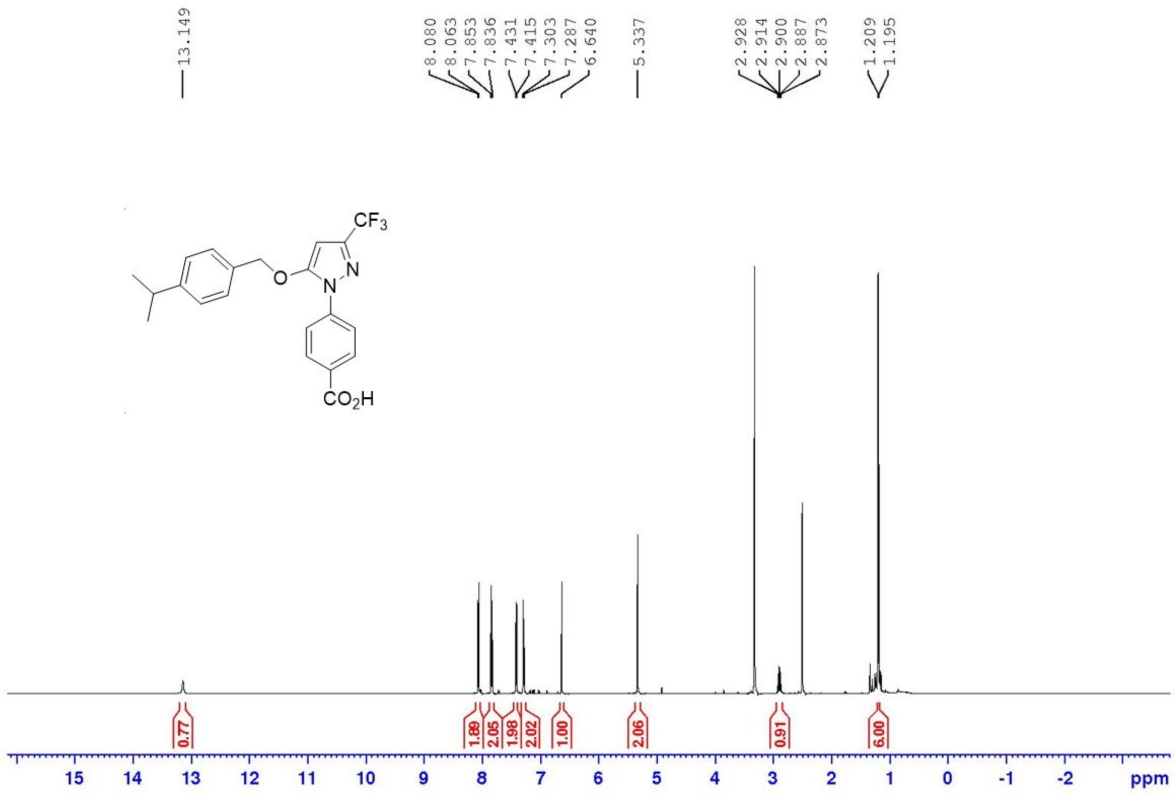

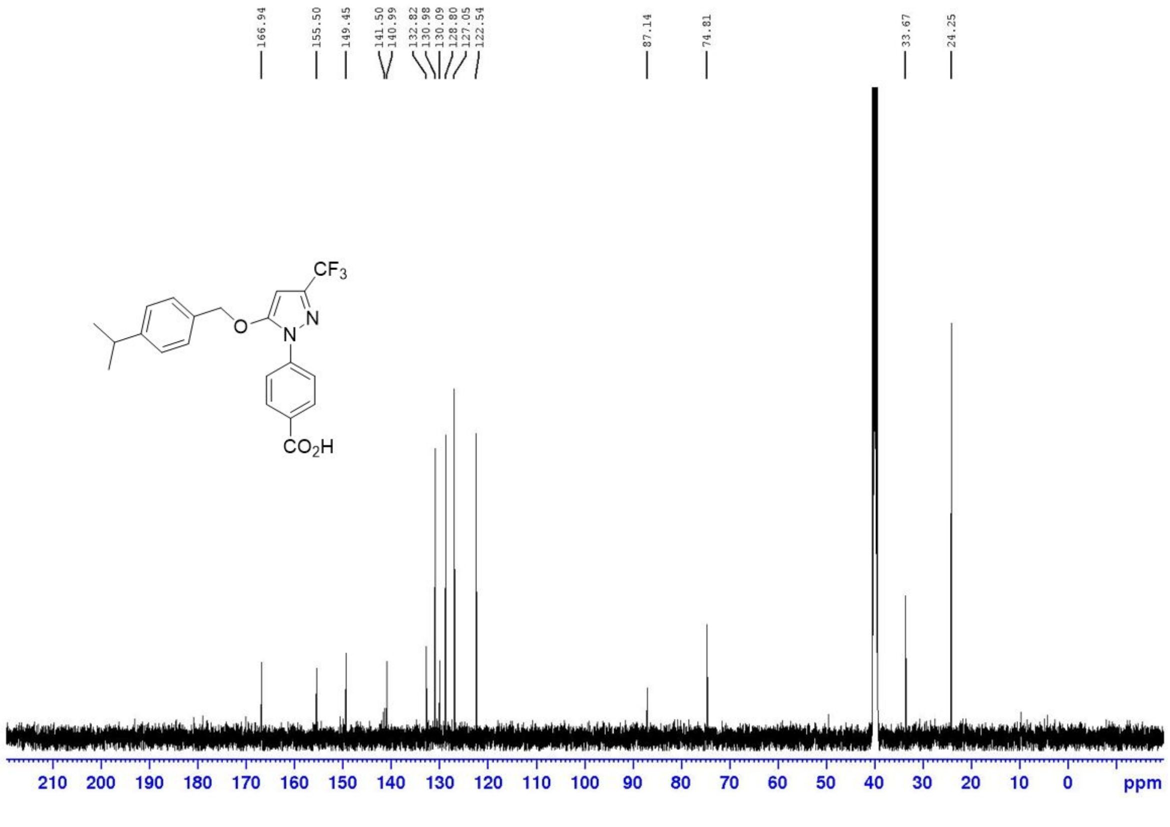

X: ethyl 4-(5-(naphthalen-2-ylmethoxy)-3-(trifluoromethyl)-1H-pyrazol-1-yl)benzoate (22a)

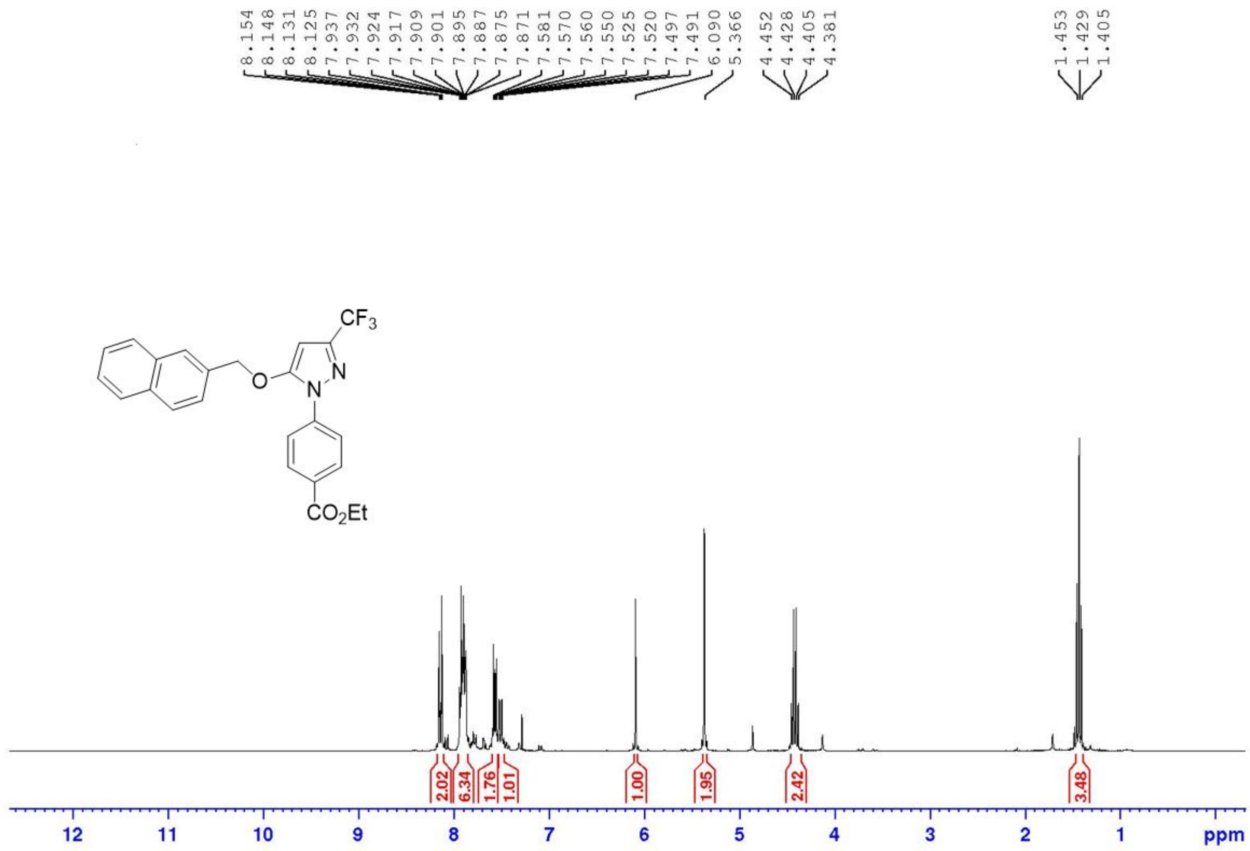

Y: 4-(5-(naphthalen-2-ylmethoxy)-3-(trifluoromethyl)-1H-pyrazol-1-yl)benzoic acid (22)

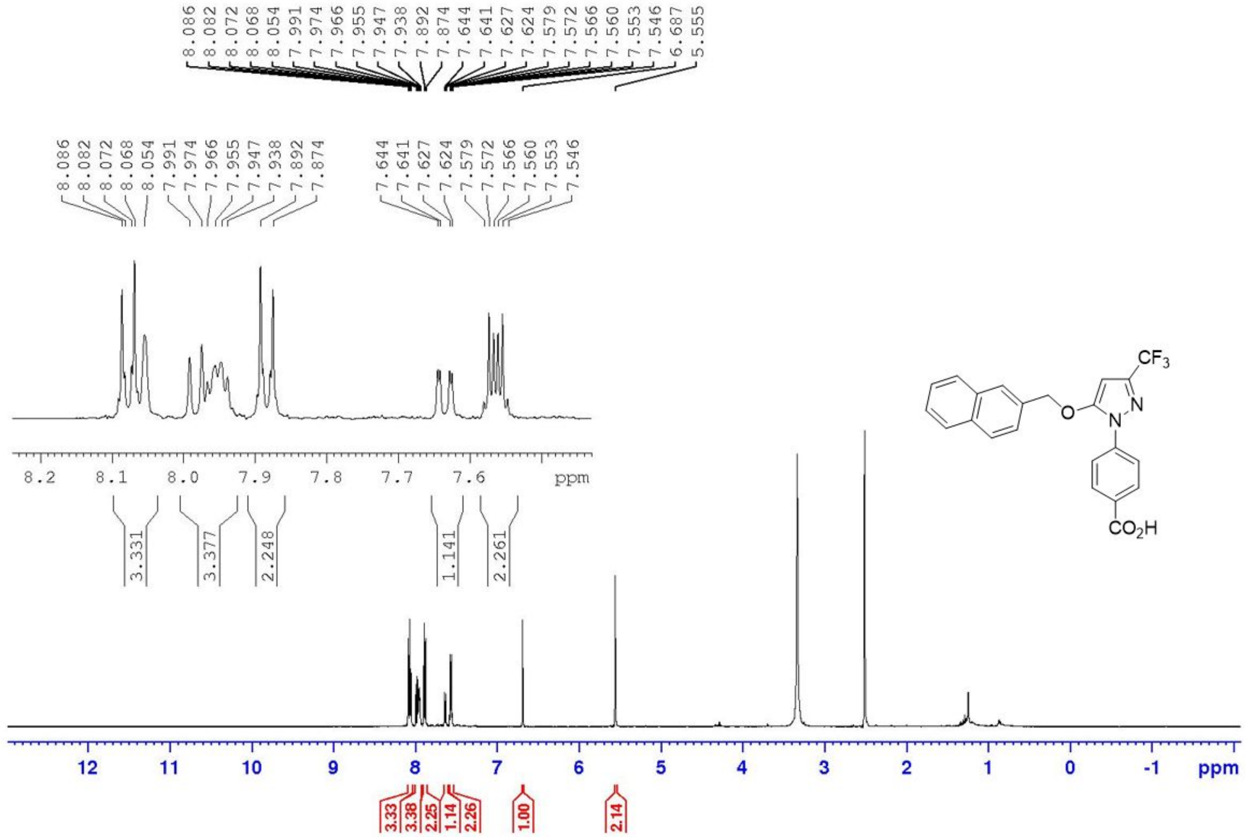

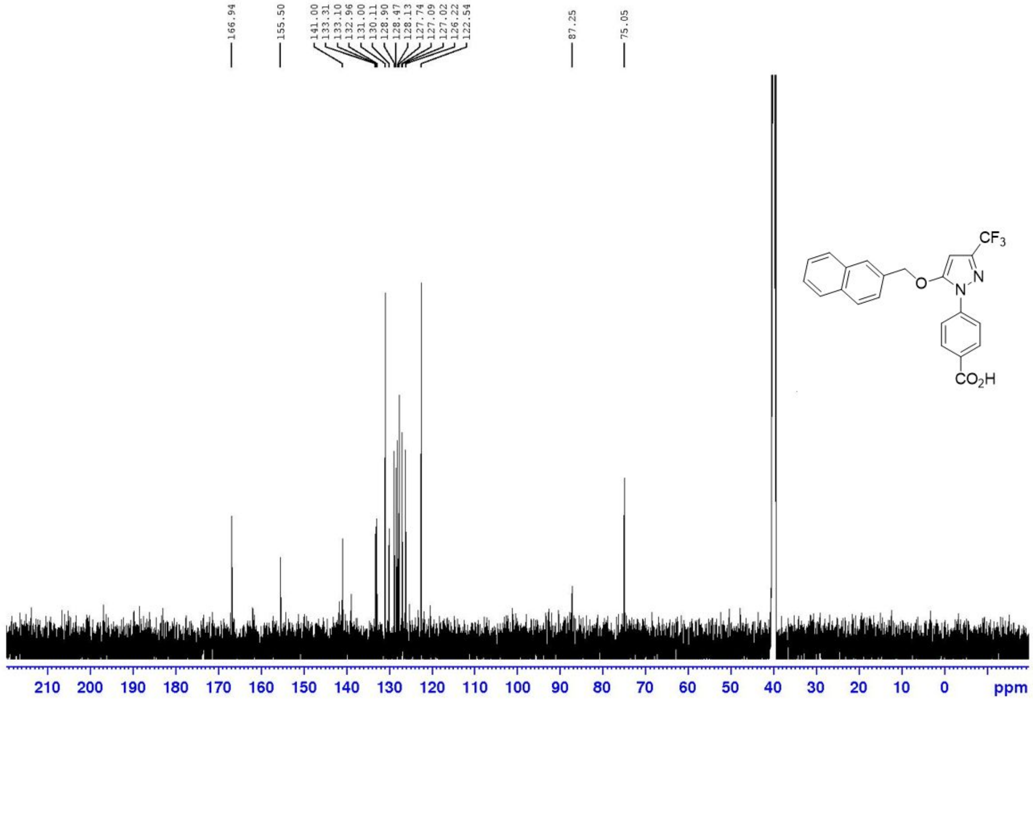

Z: 4-(5-(4-(pyridin-3-yl)phenyl)-3-(trifluoromethyl)-1H-pyrazol-1-yl)benzoic acid (23)

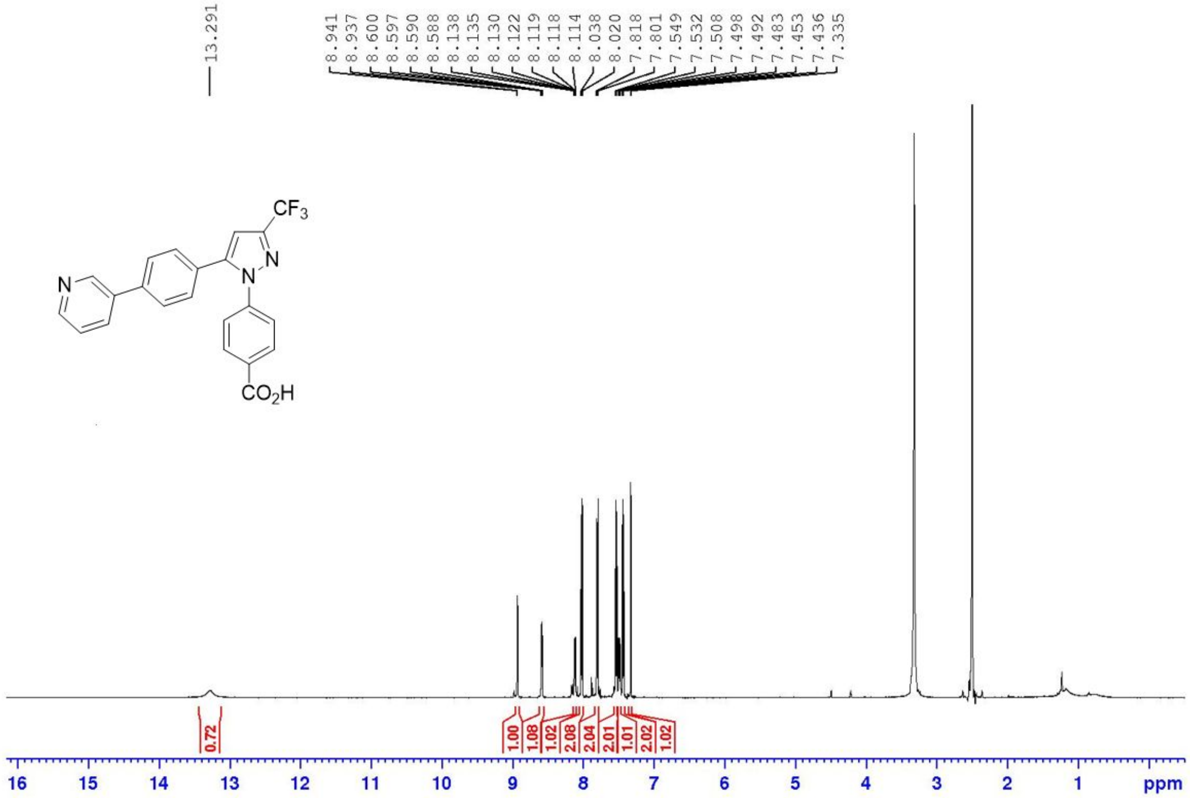

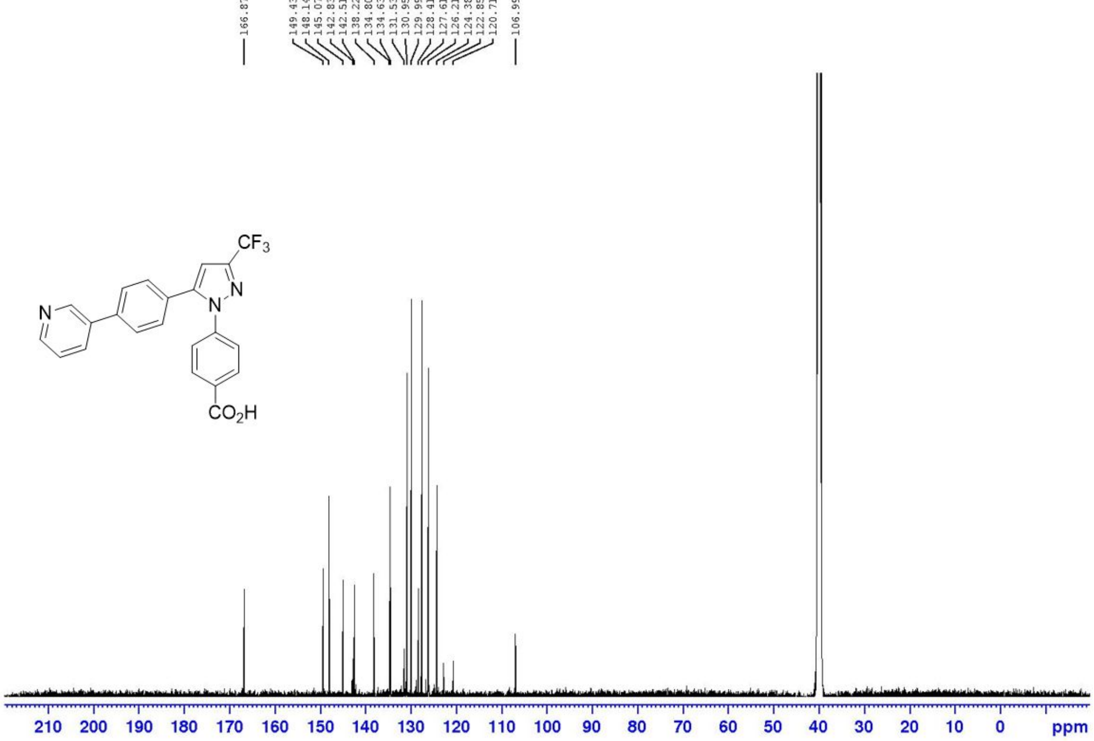

Aa: 4-(5-(4-(pyrimidin-5-yl)phenyl)-3-(trifluoromethyl)-1H-pyrazol-1-yl)benzoic acid (24)

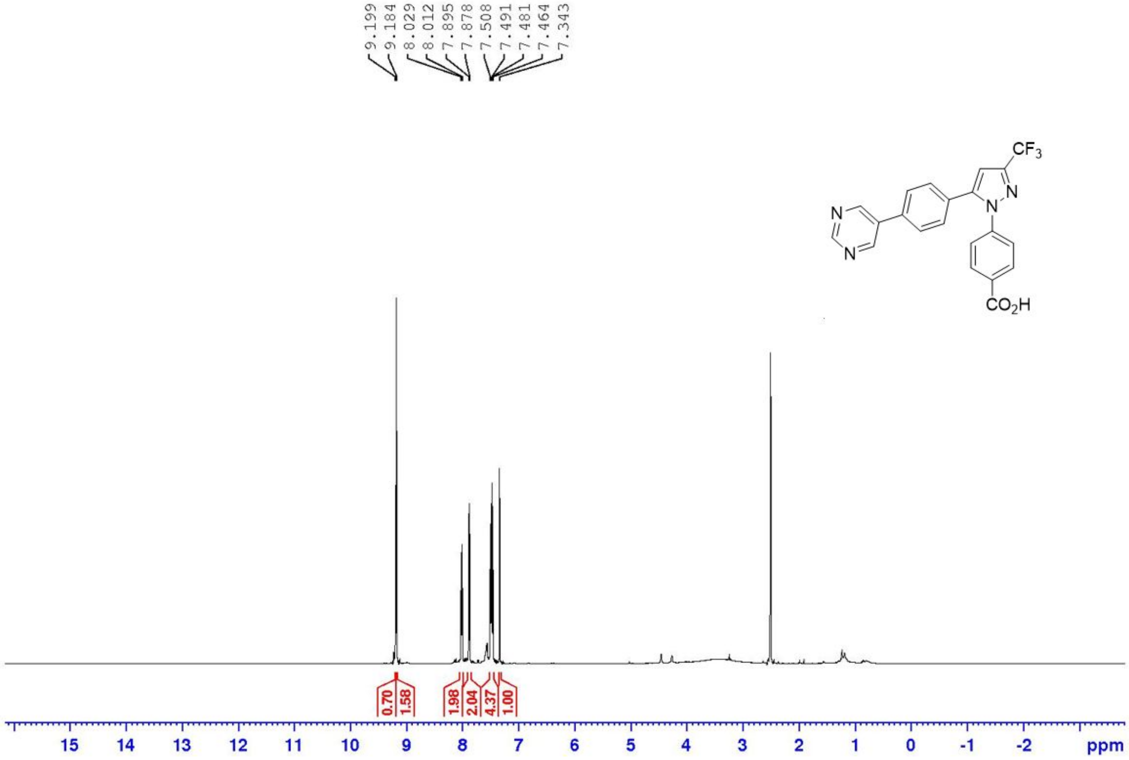

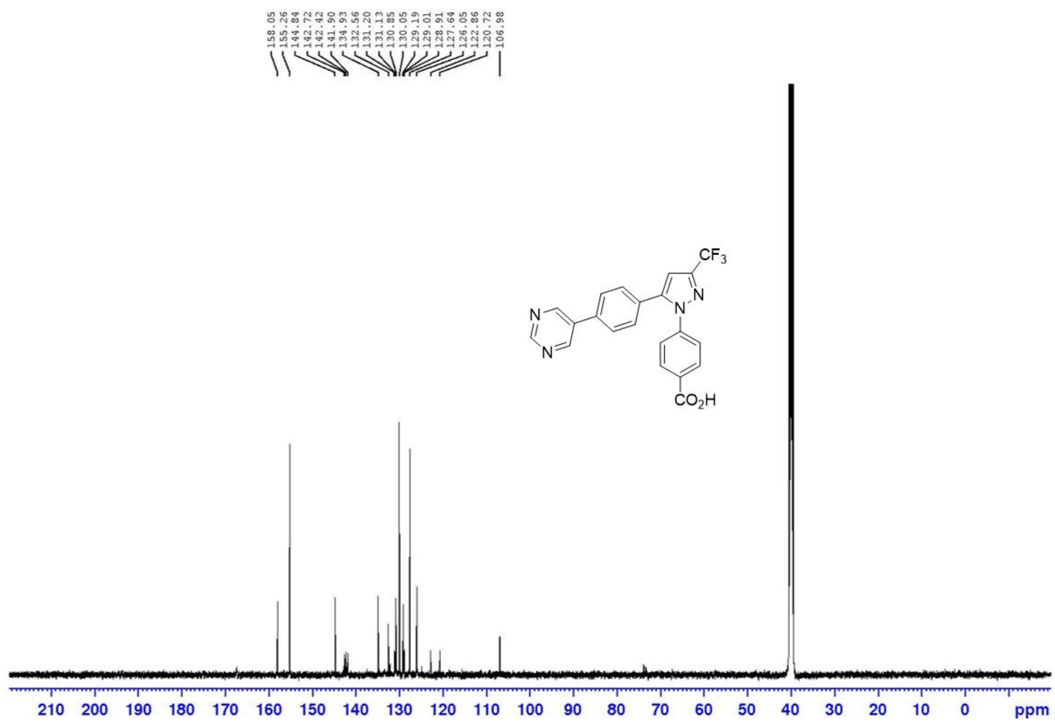

Bb: 5-(5-([1,1'-biphenyl]-4-yl)-3-(trifluoromethyl)-1H-pyrazol-1-yl)benzo[d]isothiazol-3(2H)-one 1,1-dioxide (25)

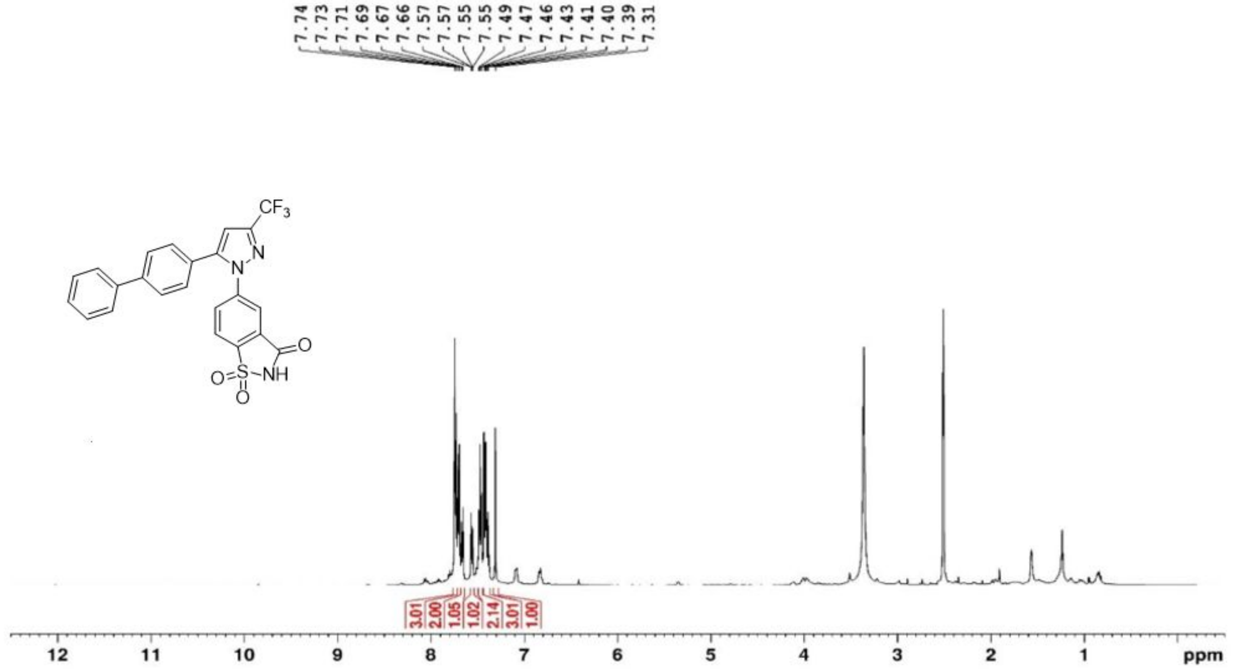

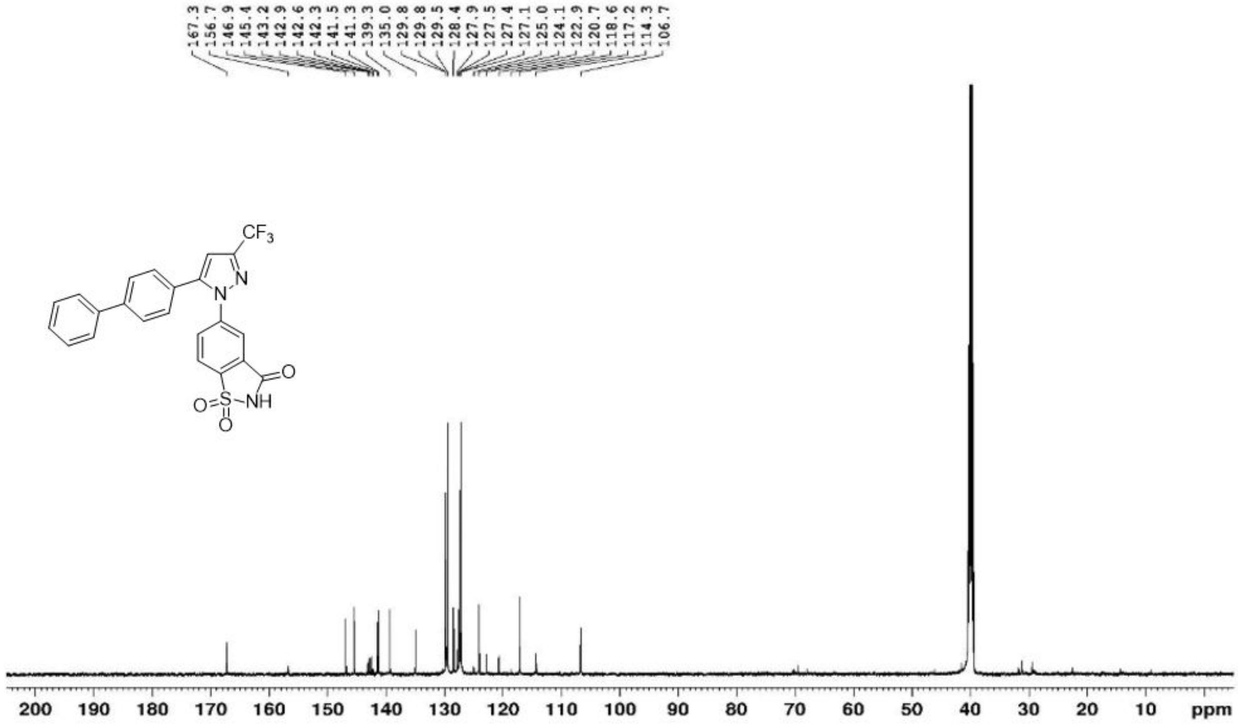

Cc: 5-(5-(naphthalen-2-yl)-3-(trifluoromethyl)-1H-pyrazol-1-yl)benzo[d]isothiazol-3(2H)-one 1,1-dioxide (26)

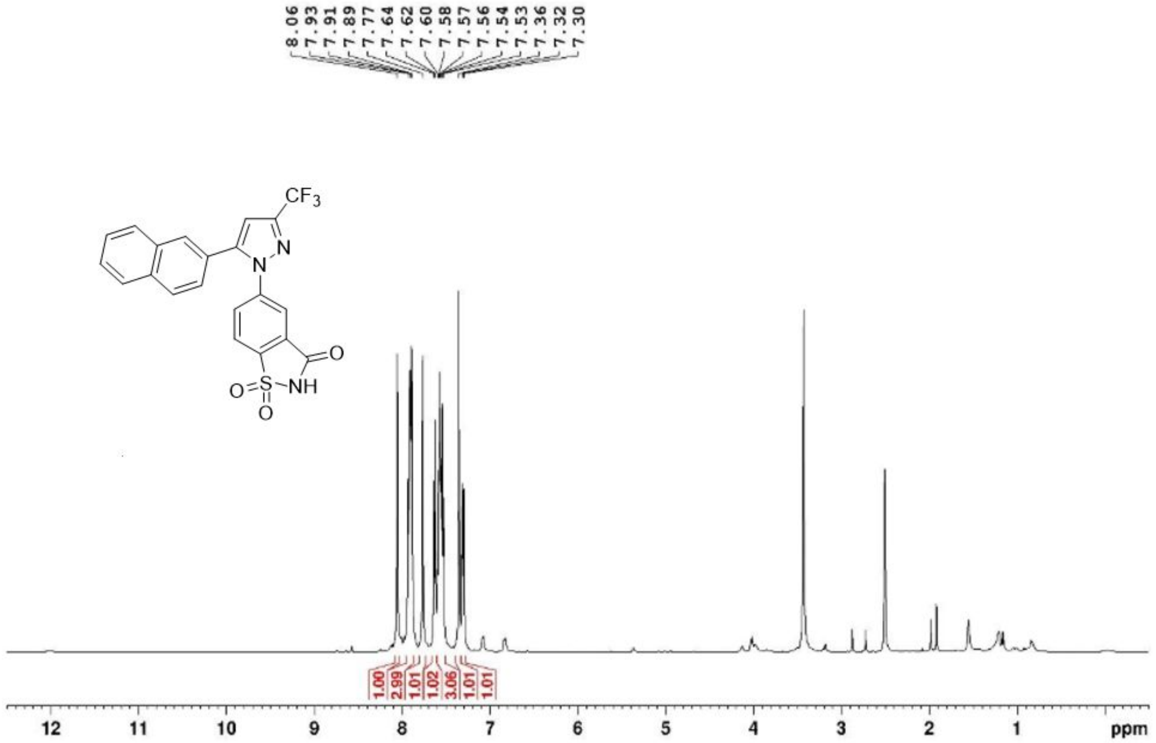

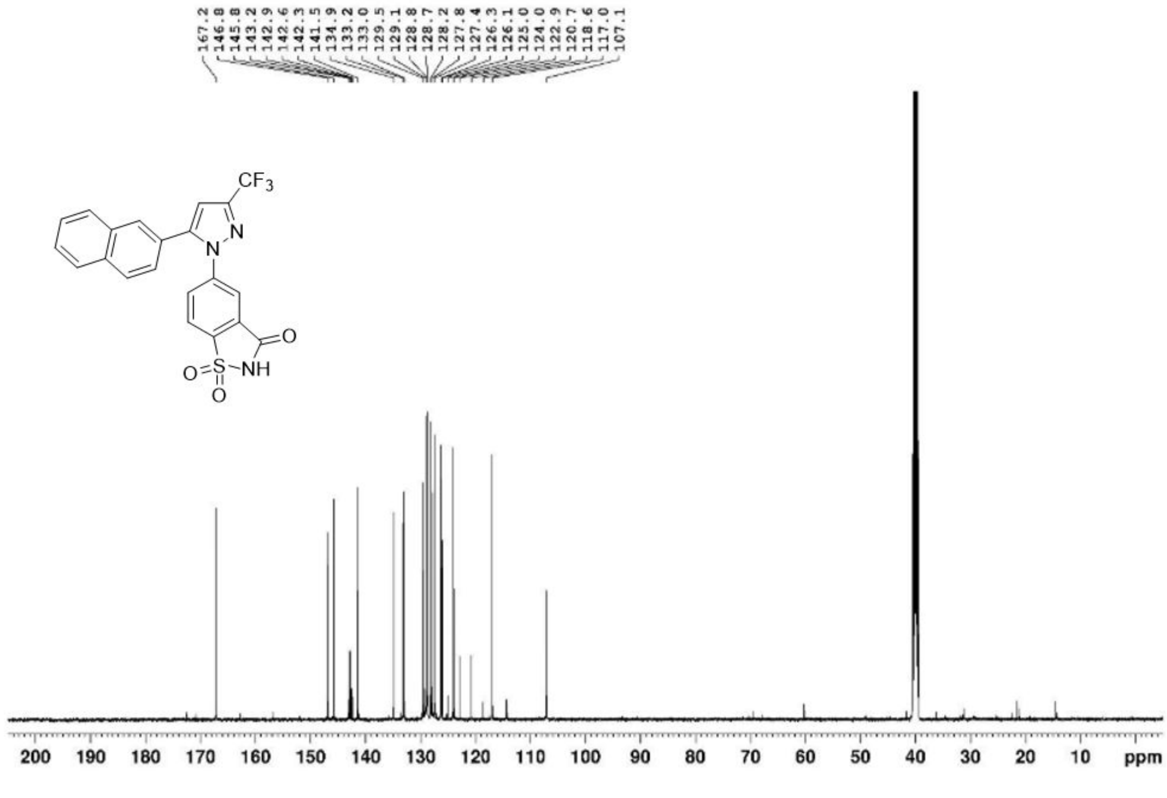

Dd: 5-(5-((4-chlorobenzyl)oxy)-3-(trifluoromethyl)-1H-pyrazol-1-yl)benzo [d]isothiazol-3(2H)-one 1,1-dioxide (27)

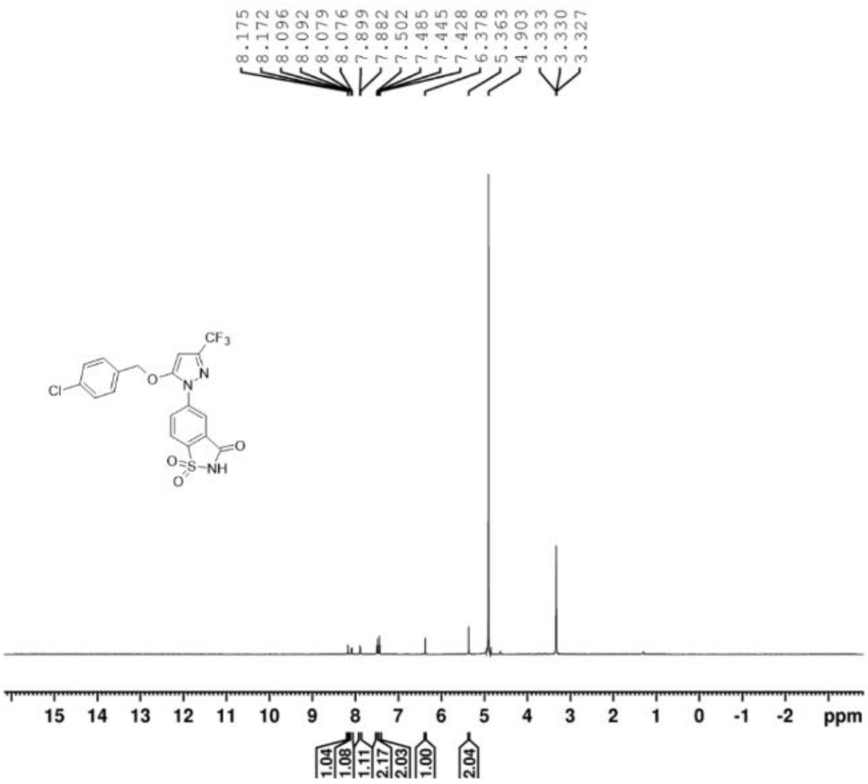

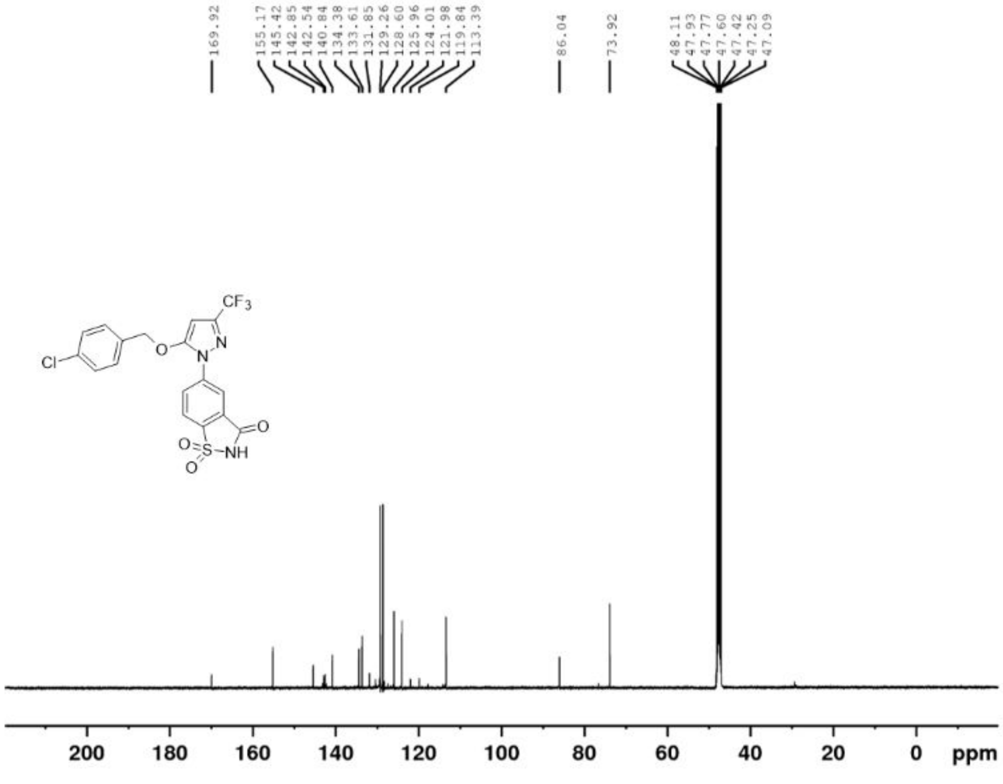

Ee: 4-(5-((4-chlorobenzyl)oxy)-3-(trifluoromethyl)-1H-pyrazol-1-yl)benzenesulfonamide (28)

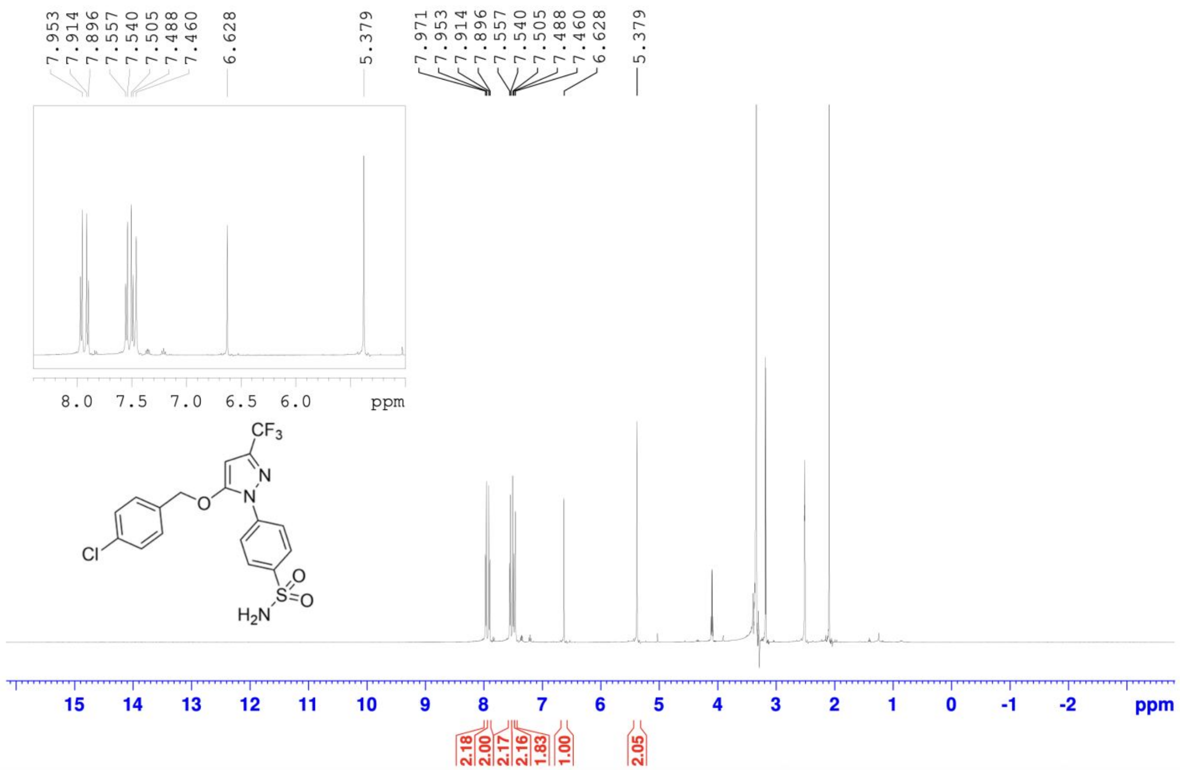

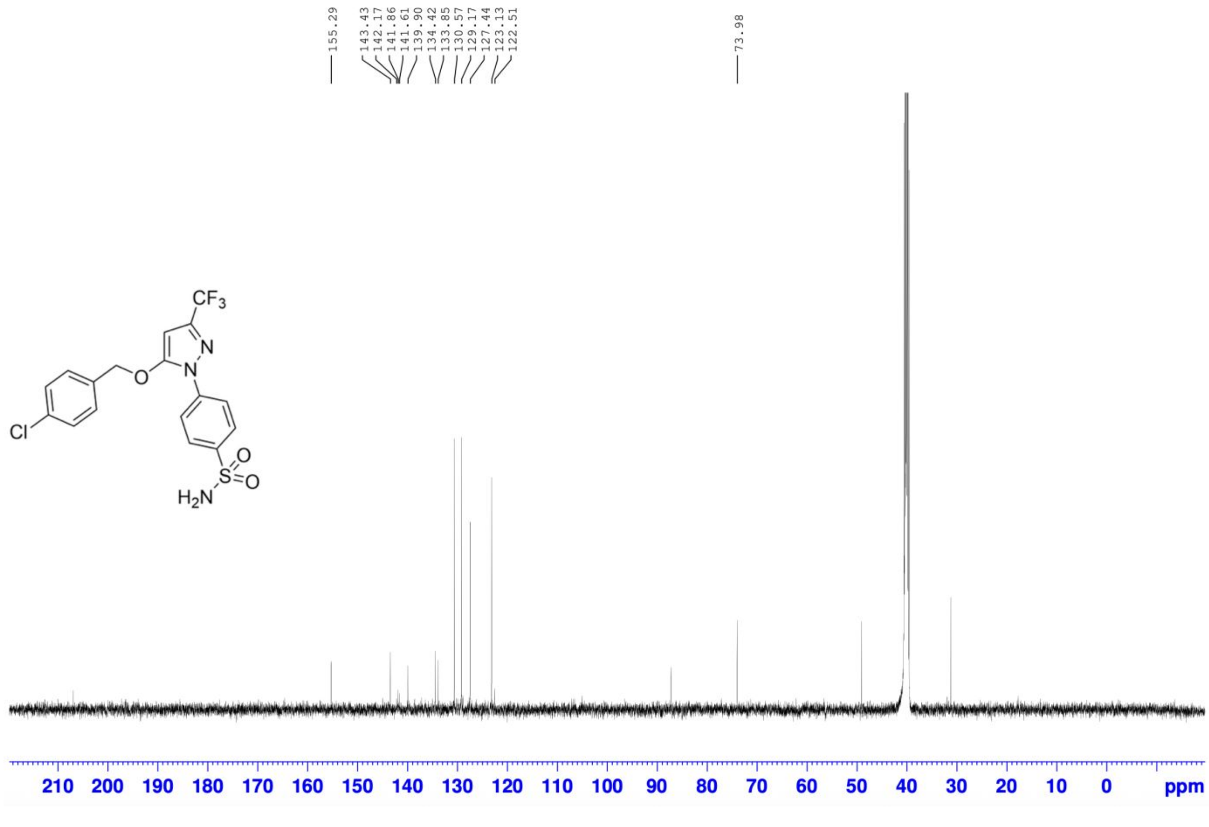

Ff: 4-(5-((4-fluorobenzyl)oxy)-3-(trifluoromethyl)-1H-pyrazol-1-yl)benzenesulfonamide (29)

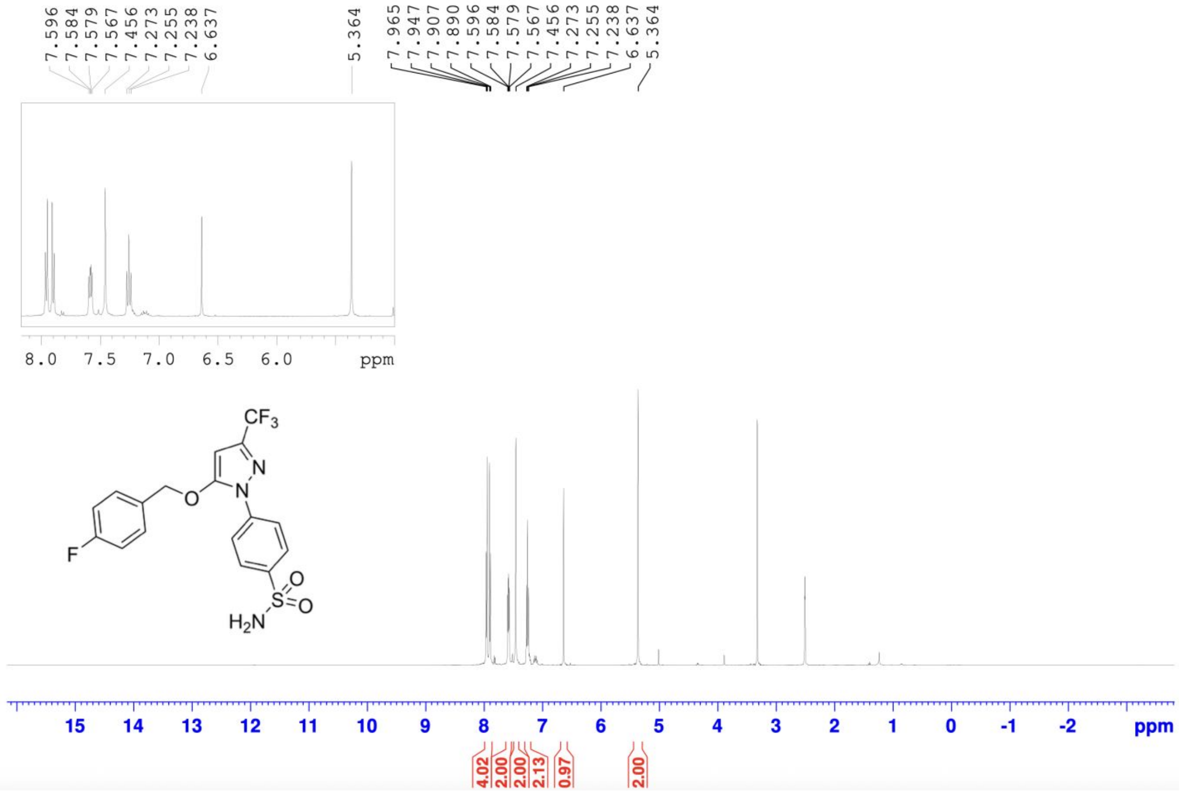

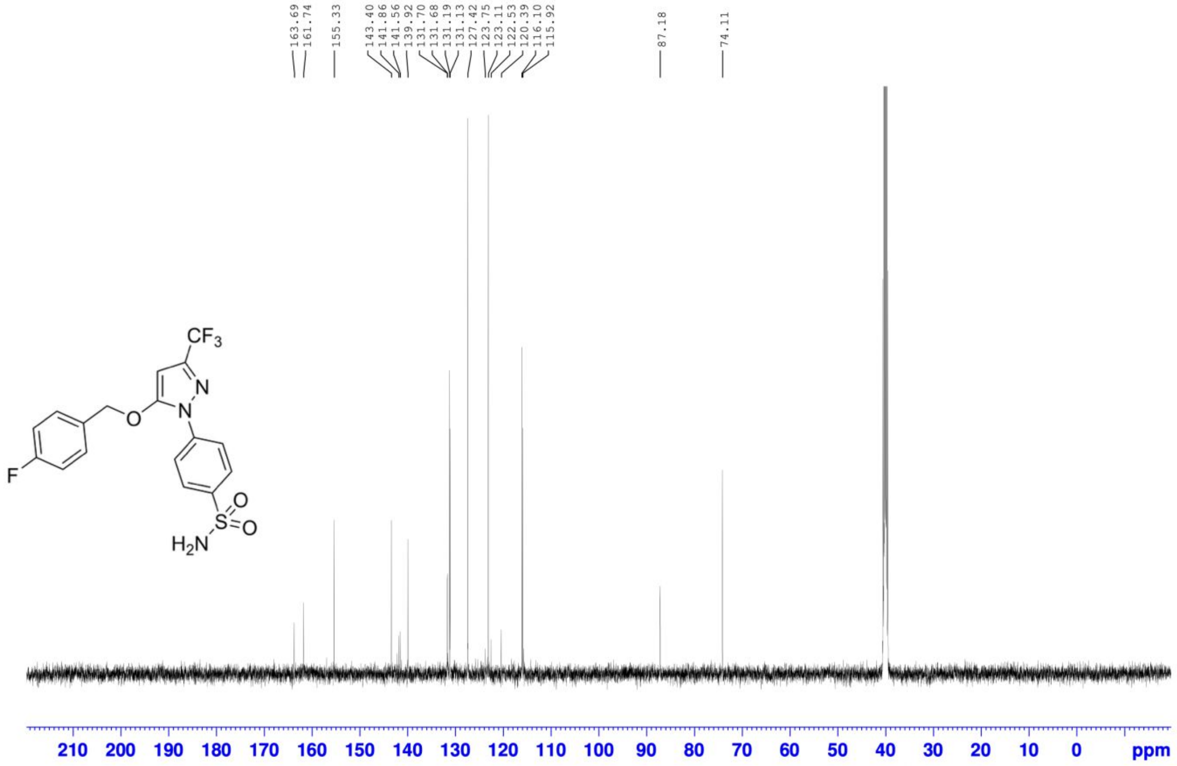

Gg: 4-(5-((4-isopropylbenzyl)oxy)-3-(trifluoromethyl)-1H-pyrazol-1-yl)benzenesulfonamide (30)

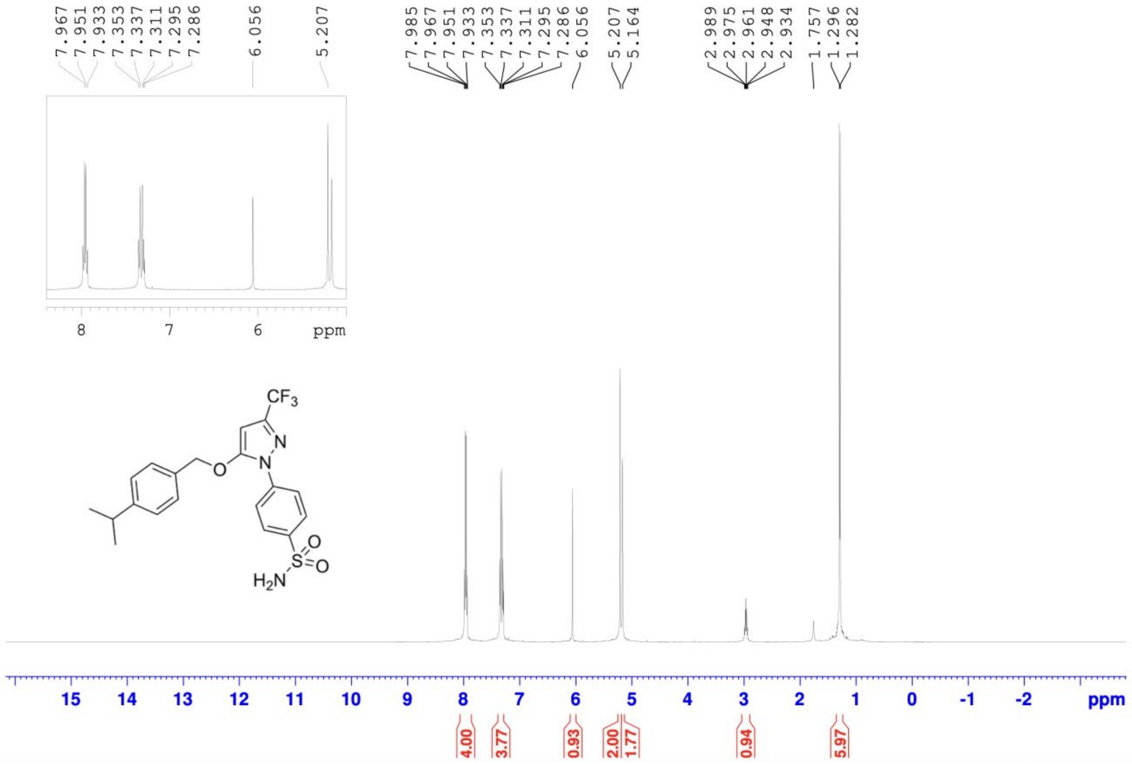

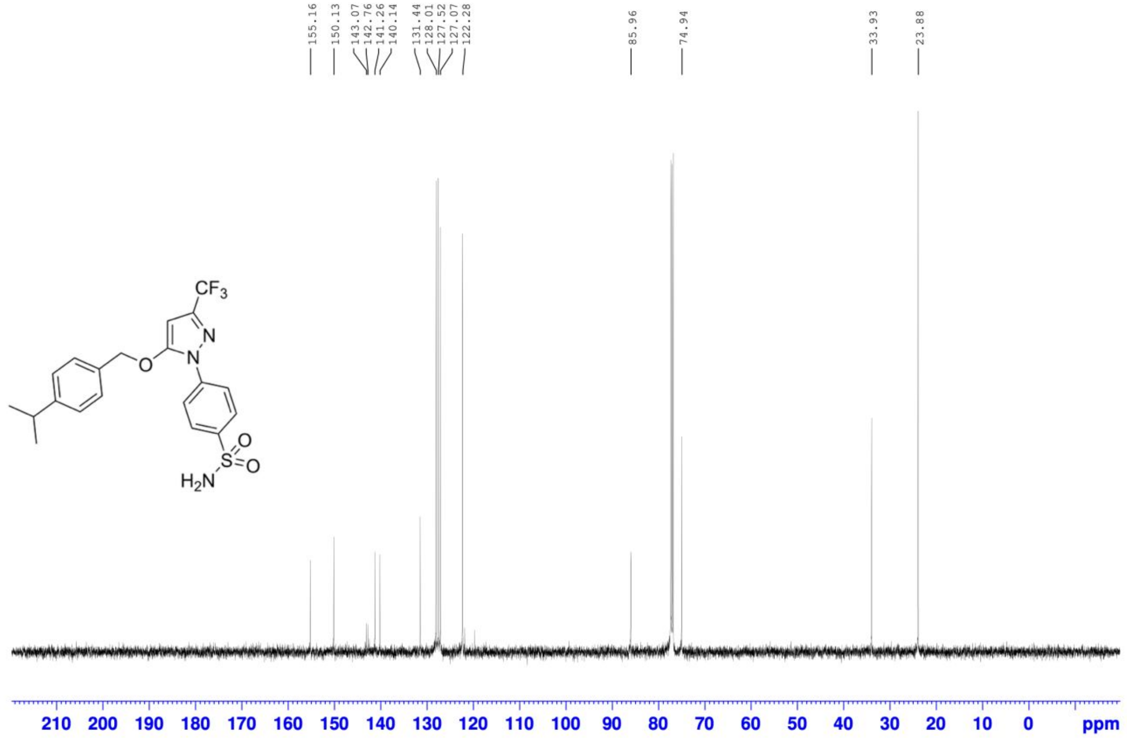

Hh: 4-(5-((4-methylbenzyl)oxy)-3-(trifluoromethyl)-1H-pyrazol-1-yl)benzenesulfonamide (31)

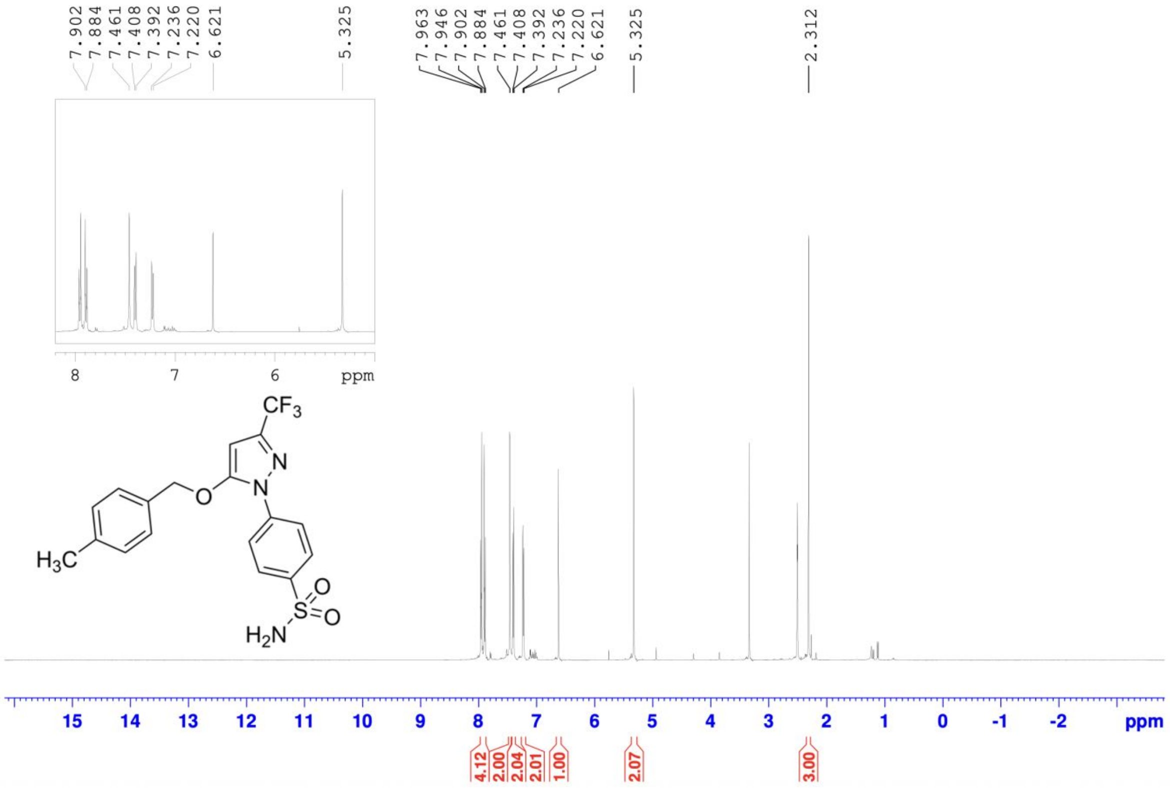

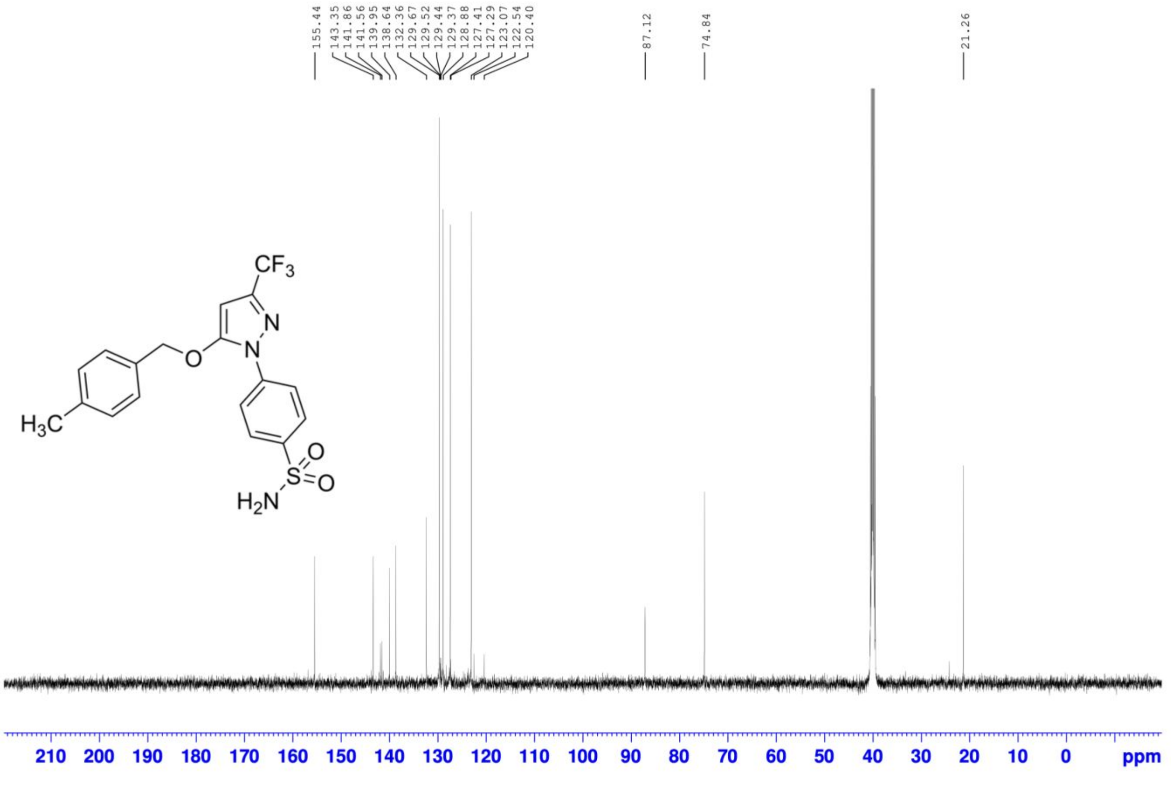

Ii: 4-(5-((4-(tert-butyl)benzyl)oxy)-3-(trifluoromethyl)-1H-pyrazol-1-yl)benzenesulfonamide (32)

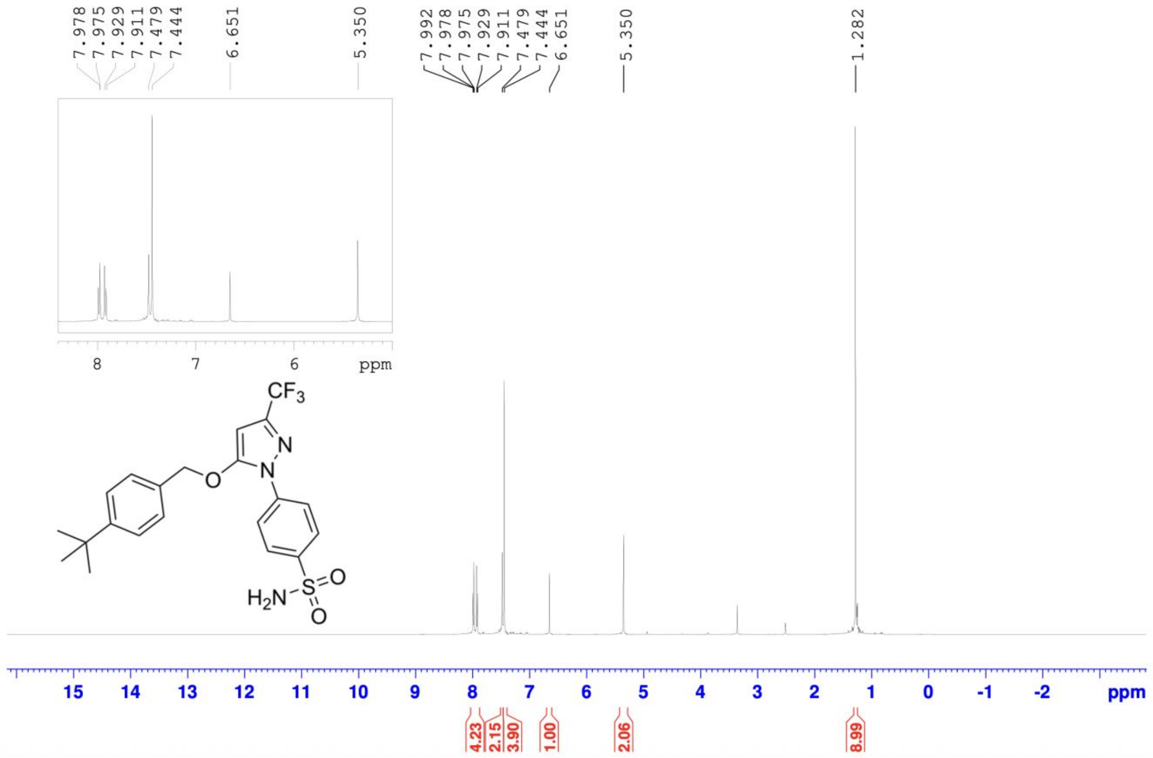

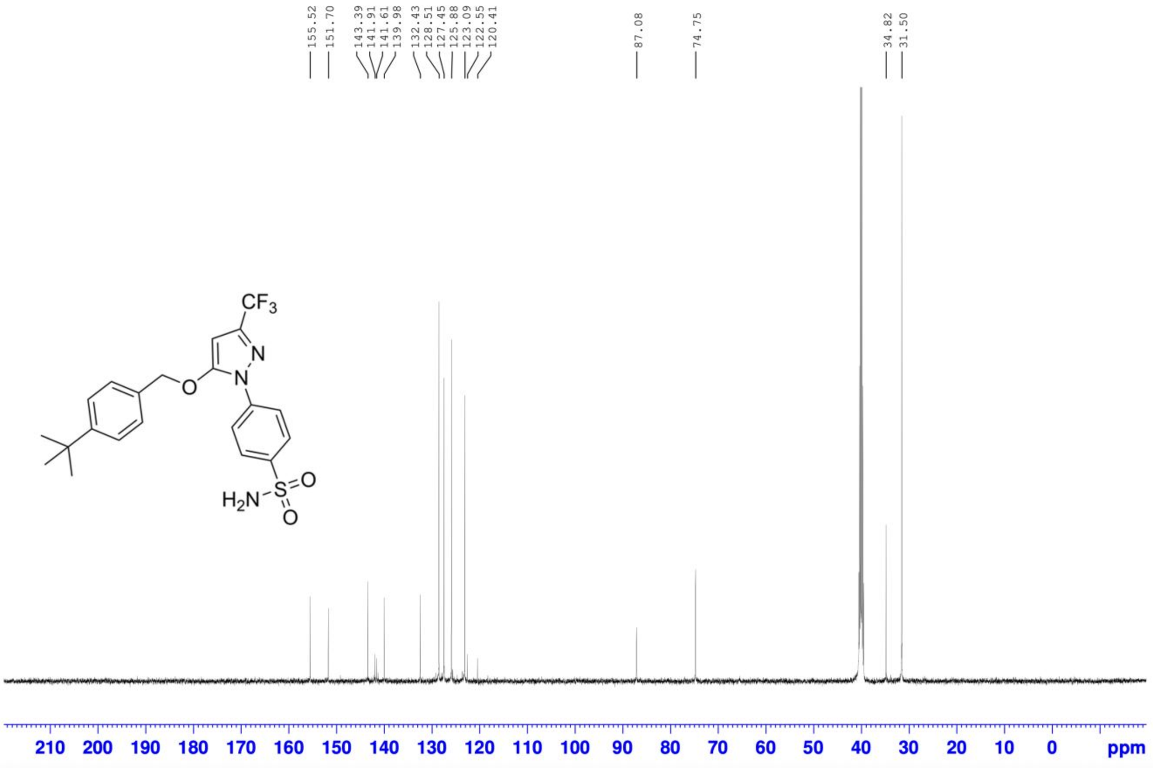

Jj: 4-(5-ethoxy-3-(trifluoromethyl)-1H-pyrazol-1-yl)benzenesulfonamide (33)

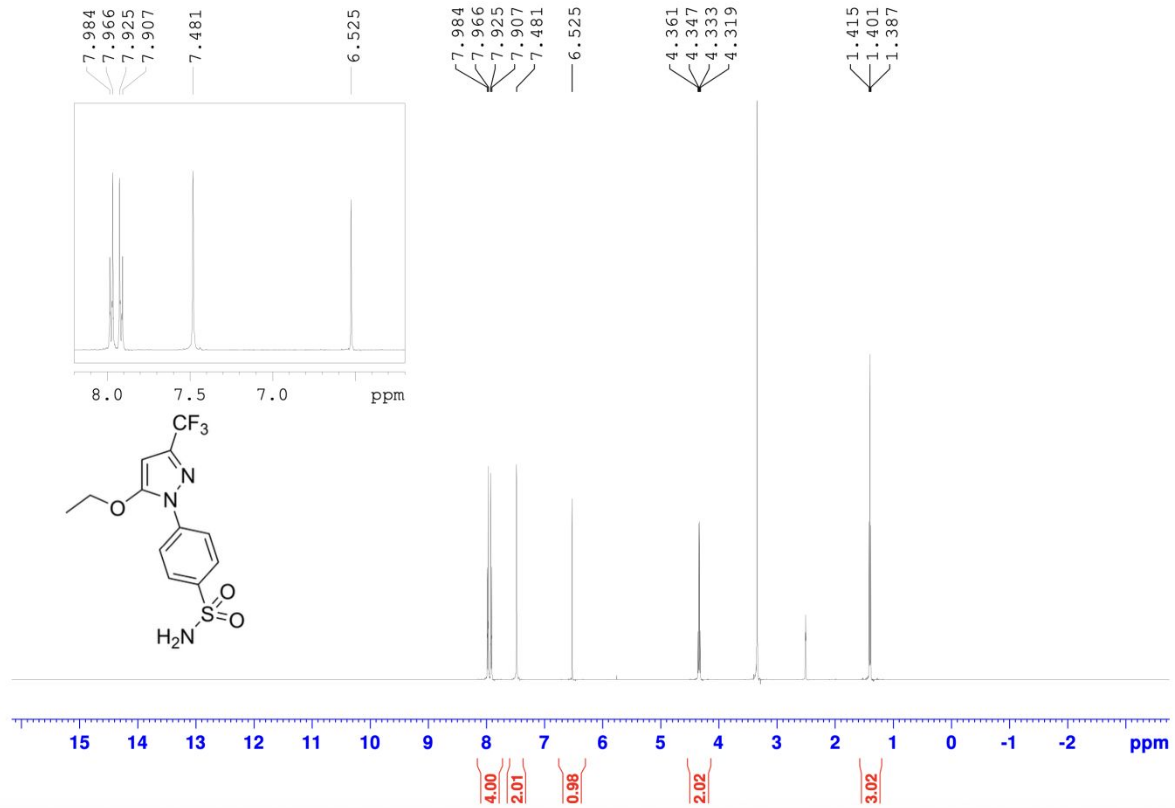

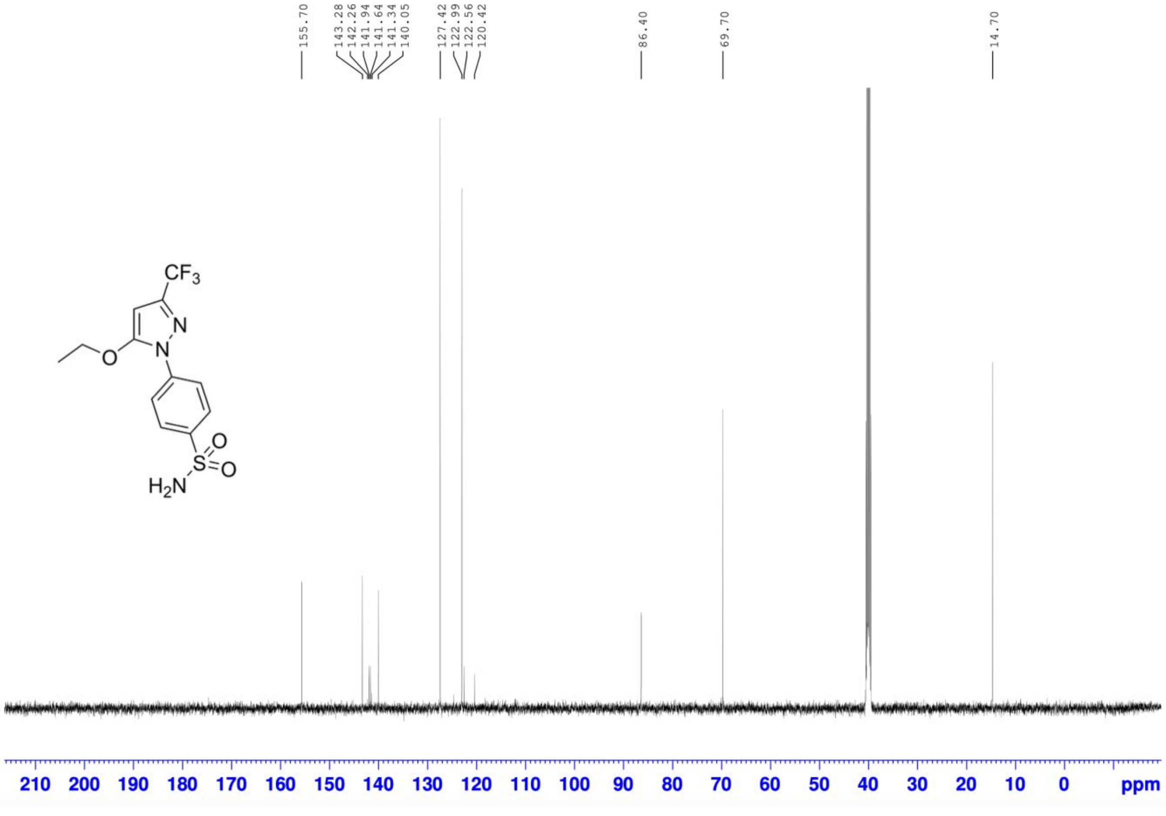

Kk: 4-(5-(benzyloxy)-3-(trifluoromethyl)-1H-pyrazol-1-yl)benzenesulfonamide (34)

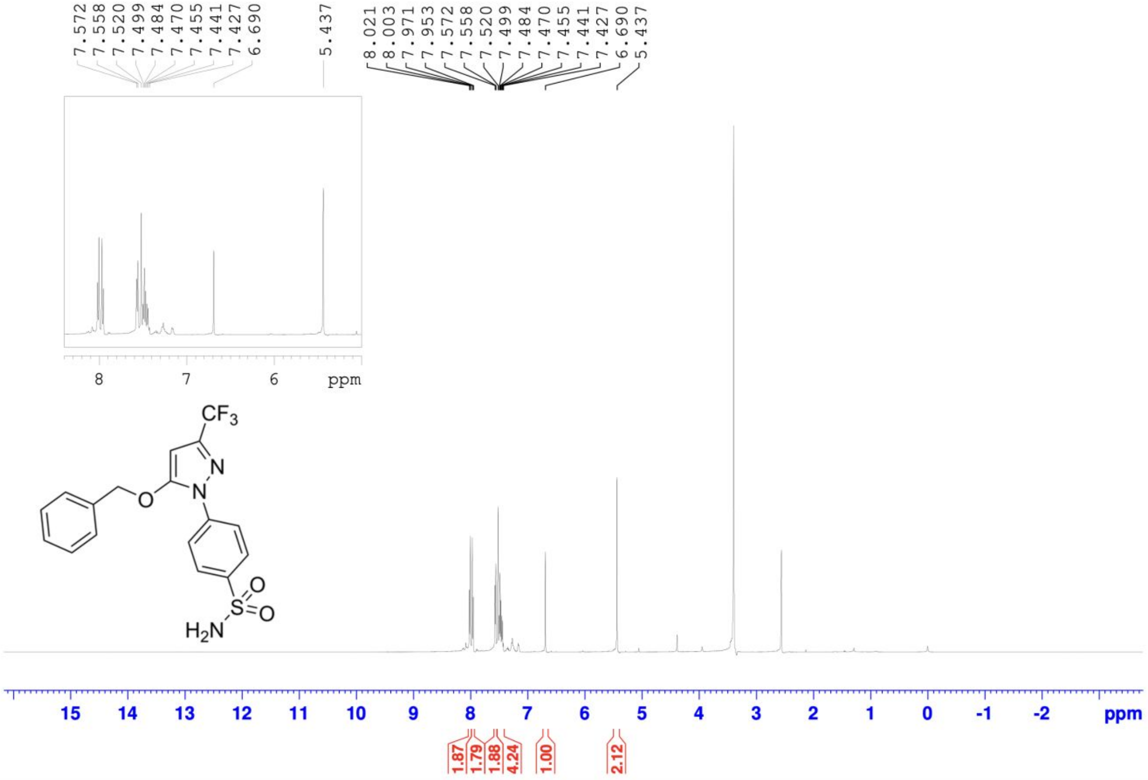

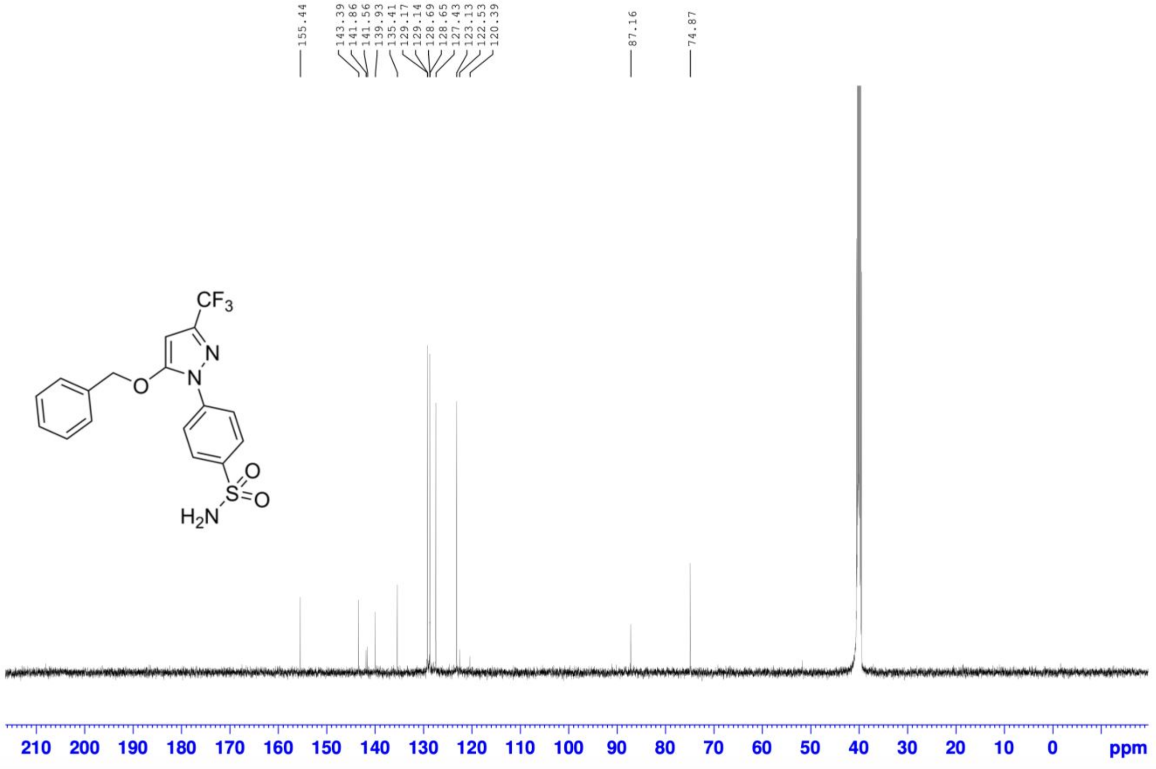

Ll: 4-(5-(naphthalen-2-ylmethoxy)-3-(trifluoromethyl)-1H-pyrazol-1-yl)benzenesulfonamide (35)

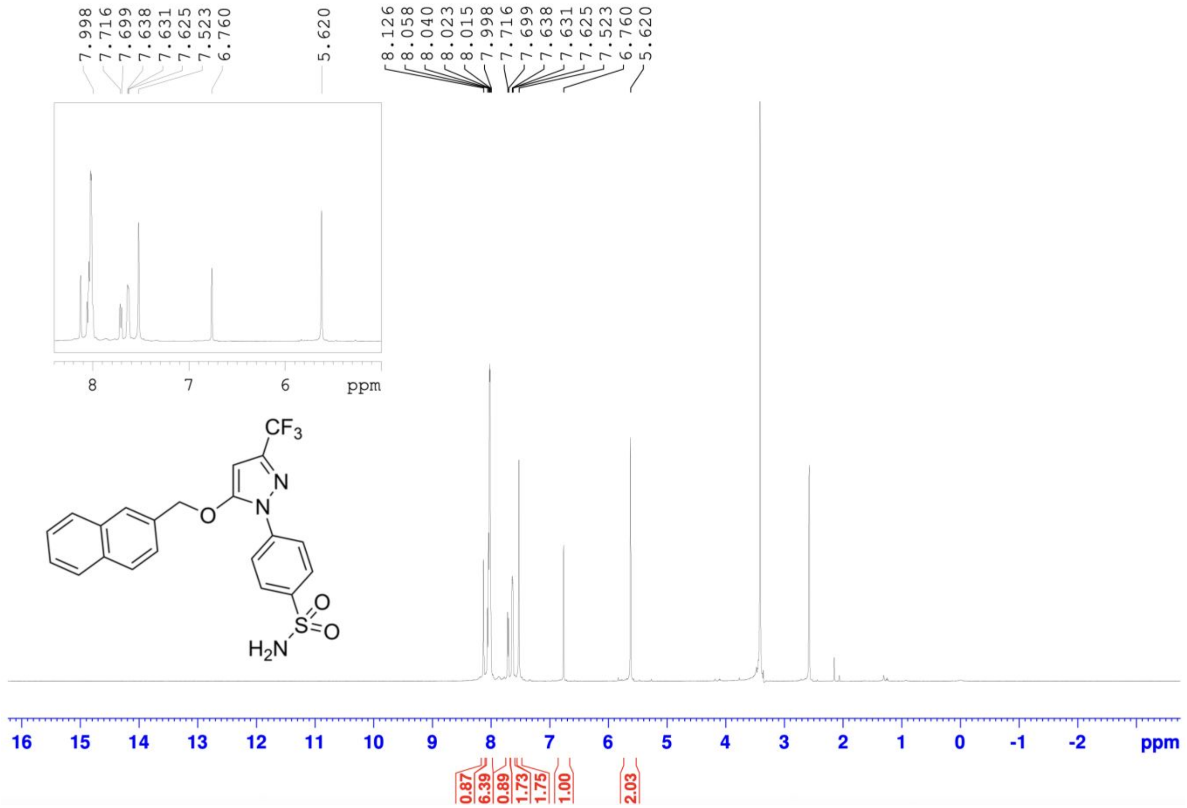

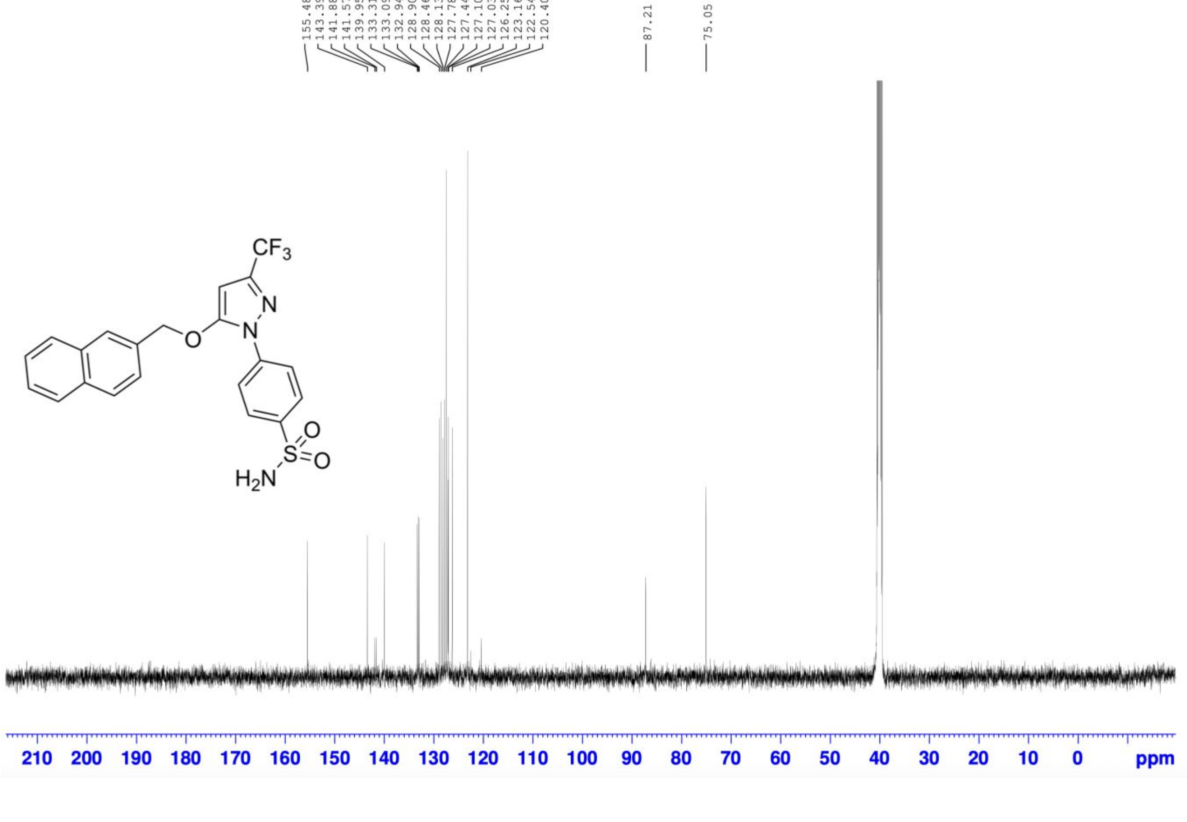

Mm: 4-(5-(naphthalen-1-ylmethoxy)-3-(trifluoromethyl)-1H-pyrazol-1-yl)benzenesulfonamide (36)

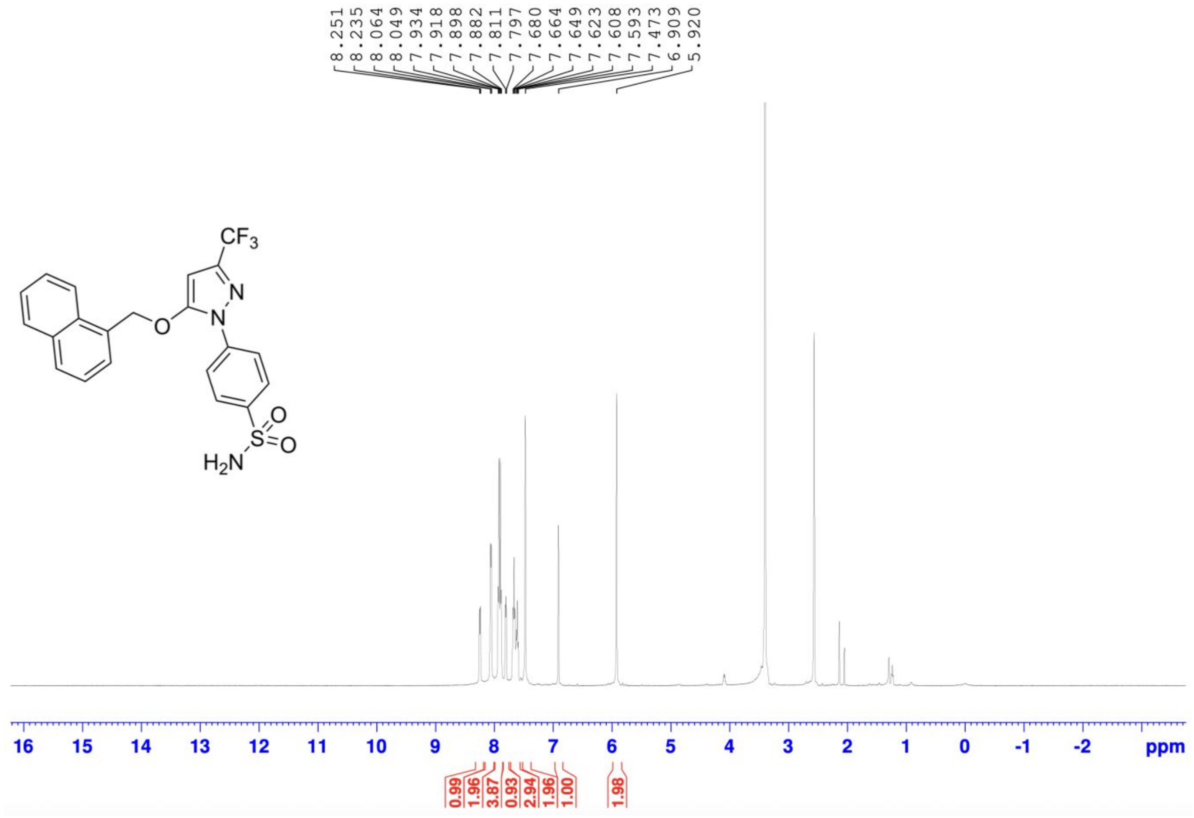

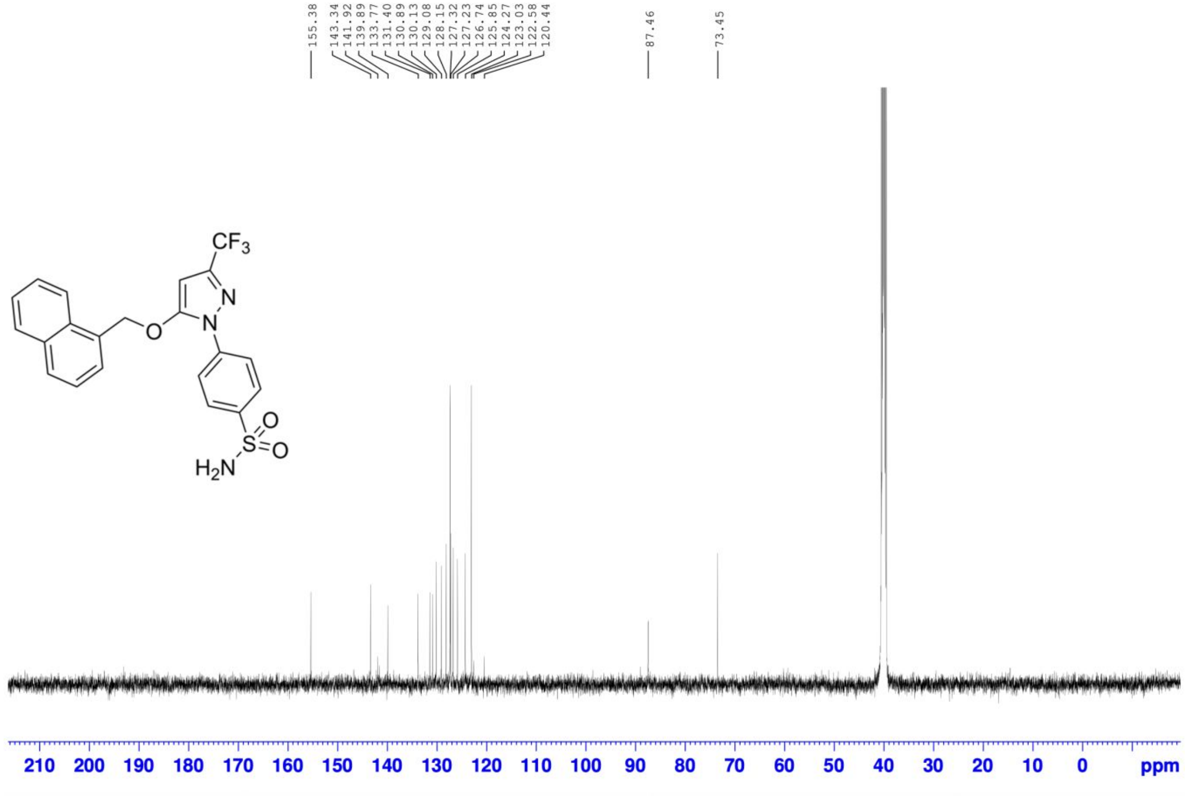

Supplement: Supplementary file 1 [file ao5c13157_si_001.pdf]
